# Targeted AAV gene therapy for neuroblastoma via direct capsid-antibody coupling

**DOI:** 10.1038/s44321-026-00520-7

**Published:** 2026-09-12

**Authors:** Mirko Luoni, Stefano Parracino, Serena G Giannelli, Francesca Morosi, Elisa Ventura, Agostina Di Pizio, Sharon Muggeo, Angelo Iannielli, Silvia Sicardi, Tamara Canu, Tommaso Russo, Antonio Esposito, Ubaldo Del Carro, Fabio Pastorino, Vania Broccoli

**Affiliations:** 1 CNR Institute of Neuroscience, Milan, Italy; 2 Institute of Experimental Neuroscience, IRCCS San Raffaele Scientific Institute, Milan, Italy; 3 Neurophysiology Service, IRCCS San Raffaele Scientific Institute, Milan, Italy; 4 Preclinical Imaging Facility, IRCCS San Raffaele Scientific Institute, Milan, Italy; 5 Experimental neuropathology Unit, IRCCS San Raffaele Scientific Institute, Milan, Italy; 6 Laboratory of Experimental Therapies in Oncology, IRCCS Istituto Giannina Gaslini, Genoa, Italy

**Keywords:** Cancer, Genetics, Gene Therapy & Genetic Disease, Neuroscience

## Abstract

Adeno-associated viral (AAV) vectors are widely used in gene therapy for their versatility and safety, but their broad tropism limits cell-specific applications such as targeting primary or metastatic tumor cells. To address this, we developed AAV-STITCH, a strategy using SpyTag technology to covalently attach polypeptides to the AAV capsid. This allows precise, dose-dependent coupling of an anti-GD2 scFv to a galactose-binding-deficient AAV9-W503A capsid, redirecting tropism specifically to GD2-expressing neuroblastoma (NB) cells. In pseudometastatic xenograft mouse models, AAV-STITCHαGD2 selectively transduced NB tumor cells without transduction of healthy liver tissue. Furthermore, delivery of a suicide gene via AAV-STITCHαGD2 significantly slowed tumor growth and extended survival in mice with subcutaneous and pseudometastatic NB xenografts. When combined with standard-of-care chemotherapy for relapsed NB, AAV-STITCHαGD2 produced robust curative effects. Collectively, these results demonstrate the feasibility of engineering AAVs with highly specific transduction properties, providing a versatile platform for targeted tumor cell suppression and advancing the development of next-generation, precision gene therapies for cancer.

## Synopsis

A modular AAV platform enables antibody-directed viral tropism through covalent capsid engineering. Selective systemic targeting of neuroblastoma was achieved, allowing efficient suicide-gene delivery with potent antitumor activity and minimal off-target transduction.

- A modular AAV-STITCH platform enables covalent coupling of scFvs to an infectivity-deficient AAV9 capsid through SpyTag/SpyCatcher chemistry.
- Systemic administration selectively transduced GD2⁺ neuroblastoma cells while largely abolishing transduction of healthy tissues.
- AAV-STITCHαGD2-TK produced potent antitumor activity, reducing metastatic tumor burden and prolonging survival after a single treatment.
- Combining AAV-STITCHαGD2-TK with TOTEM chemotherapy resulted in near-complete suppression of tumor growth, supporting complementary therapeutic mechanisms.

## Abstract (web summary)

A modular AAV platform enables antibody-directed viral tropism through covalent capsid engineering. Selective systemic targeting of neuroblastoma was achieved, allowing efficient suicide-gene delivery with potent antitumor activity and minimal off-target transduction.

## Graphical abstract

**Figure:**
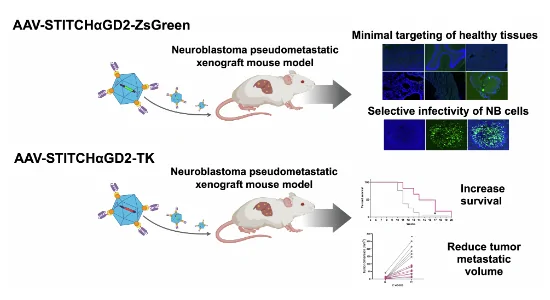

The paper explained **Problem**

High-risk neuroblastoma remains one of the deadliest childhood cancers despite multimodal treatment combining chemotherapy, surgery, radiotherapy, stem cell transplantation and anti-GD2 immunotherapy. Current targeted therapies, including anti-GD2 antibodies and CAR-T cells, have improved patient outcomes but are limited by severe toxicities, immune-related complications and reduced efficacy within the immunosuppressive tumor microenvironment. Meanwhile, adeno-associated viruses (AAVs), although widely used for gene therapy, have not been successfully exploited for cancer treatment because of their broad natural tropism and inability to selectively target tumor cells while sparing healthy tissues.

**Results**

We developed AAV-STITCH, a versatile platform that redirects AAV tropism by covalently coupling antibody fragments onto an infectivity-deficient AAV capsid through SpyTag/SpyCatcher chemistry. Using an anti-GD2 antibody fragment, we generated AAV-STITCHαGD2, which selectively transduced GD2-positive neuroblastoma cells while showing negligible infection of healthy tissues following systemic administration. Packaging a suicide gene (HSV-TK) into this targeted vector produced potent antitumor activity in multiple neuroblastoma models, including metastatic and patient-derived xenografts, without detectable systemic toxicity or neurological adverse effects. Furthermore, combining AAV-STITCHαGD2 with standard chemotherapy resulted in nearly complete suppression of tumor growth, demonstrating strong therapeutic complementarity.

**Impact**

This study introduces a generalizable strategy to convert broadly tropic AAV vectors into precision gene-delivery vehicles for cancer. Beyond providing a promising therapeutic approach for high-risk neuroblastoma, the modular AAV-STITCH platform can be readily adapted to different antibodies and disease-specific surface antigens, greatly expanding the potential of targeted gene therapy in oncology. By enabling selective systemic delivery of therapeutic genes while minimizing off-target transduction, AAV-STITCH establishes a new framework for safer and more effective precision viral therapies.

## Introduction

Neuroblastoma (NB) is the most frequent and aggressive pediatric extracranial solid tumor, responsible for 15% of childhood cancer deaths (Matthay et al, 2016). Approximately half of all NB patients are classified with high-risk (HR) disease typical with distant site metastases at diagnosis. Despite recent multimodal therapeutic regimens, including chemotherapy, radiation, surgical resection and autologous stem cell transplant, the 5-year survival still remains below 50% in the high-risk group (Qiu and Matthay, 2022). Moreover, children in whom first-line therapy fails have a very low probability of recovery with subsequent treatments, and they have a dismal prognosis with a long-term survival below 10%. Thus, more effective therapies are urgently needed to offer a better prognosis to these patients. The discovery that the disialoganglioside GD2 is highly expressed in NB and other cancers of neuroectodermal origin, while being rarely present in healthy peripheral tissues has spearheaded the development of immunotherapies for these malignancies. The introduction of GD2 monoclonal antibodies, has provided a new therapeutic option, improving the survival rate in high-risk NB patients (Yu et al, 2010). However, serious adverse events, including acute pain, have limited the development of this therapy (Mora et al, 2024). GD2 was one of the first tumor-associated antigens targeted by CAR T cells in humans, and clinical trials support the efficacy of GD2-redirected CAR T cells with encouraging clinical activity in first patients (Pule et al, 2008; Straathof et al, 2020; Del Bufalo et al, 2023). However, high cellular doses can lead to systemic inflammation posing important obstacles to be yet overcome. Moreover, some GD2 expression is detectable on neuronal cell bodies in different brain regions (Kinkhabwala et al, 2022), raising concerns for neurotoxicity, although brain-related adverse effects have not yet observed in the treated patients. More in general, high-risk NBs present limited T-cell infiltration, MHC-I downregulation and low mutational burden leading to a significant immunosuppressive tumor microenvironment which can challenge the outcome of CAR T-cell therapies (Qiu and Matthay, 2022). Thus, despite these important recent therapeutic advances that have improved the prognosis of high-risk NB patients, it remains urgent the need for additional and orthogonal medical products.

AAVs are non-pathogenic viruses that can infect both dividing and quiescent cells for long-term gene expression (Wang et al, 2024). Recombinant AAVs are produced at high yield in large-scale manufacturing and have broad tropism making them the most widely used vectors for gene therapy (Deverman et al, 2018). In fact, numerous gene therapy clinical trials worldwide are currently in development using AAVs as delivery system for a vast number of human diseases. However, AAV gene therapy applications in cancer are scarce given the general nonspecific tropism of these viruses. In fact, AAV native serotypes show broad infectivity in multiple tissues, although with some preferred cell-type tropism. Despite the success in modulating the AAV infectivity pattern by targeted capsid engineering, it is missing a convenient approach to attain cell-type specific viral transduction (Haery et al, 2019). We developed a new and powerful strategy to covalently couple antibody fragments on the AAV capsid surface in order to control viral infectivity for achieving selective transduction of NB cancer cells. These modified capsids demonstrated effective and safe anti-tumoral activity in xenograft NB mouse models, validating their strong translational therapeutic potential.

## Results

### Efficient and direct coupling of an αGD2-scFv to the AAV9 capsid by the SpyTag technology

**Figure 1:**
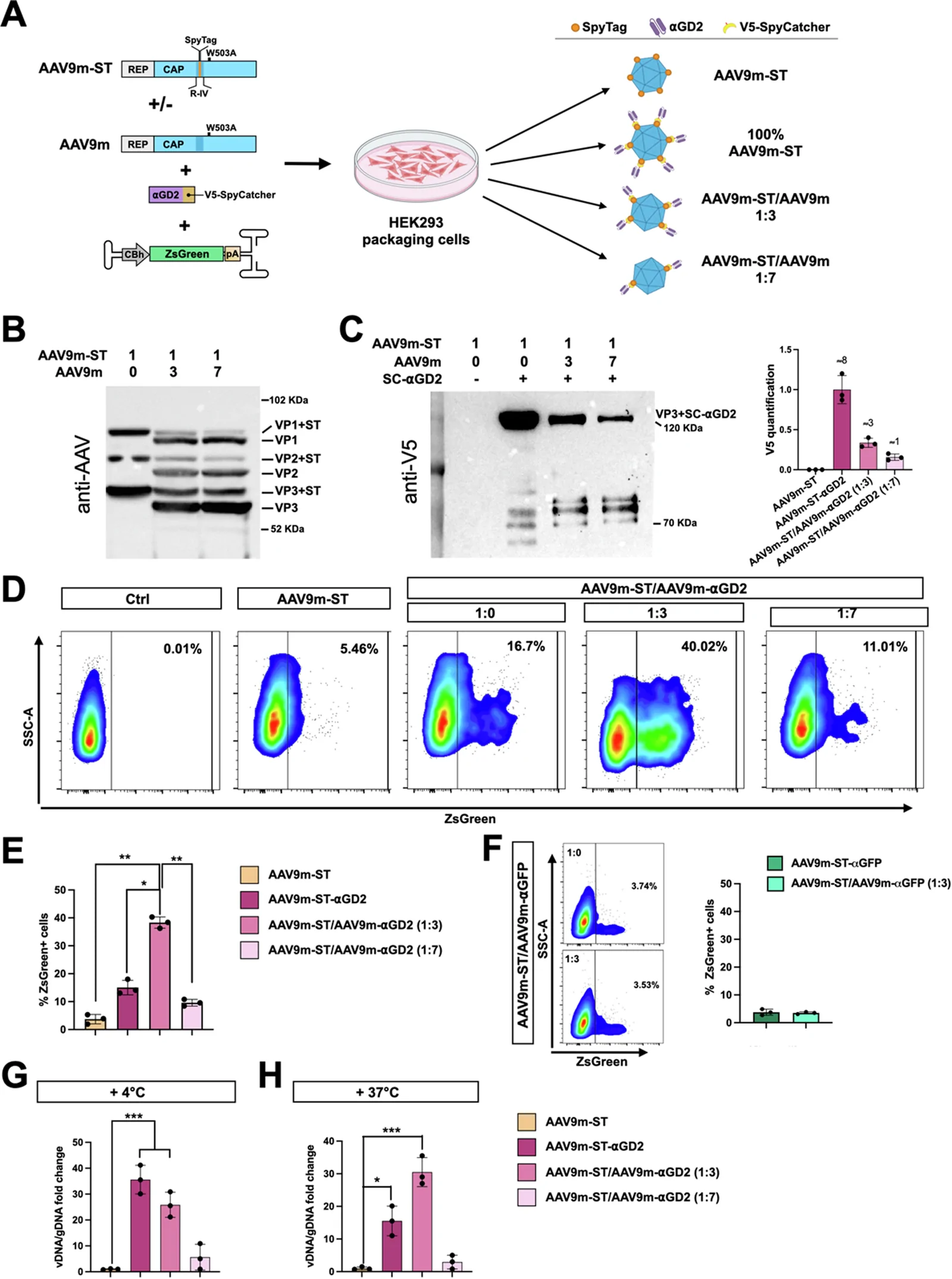
Production and functional validation of mosaic AAV-STITCHαGD2 vectors. (**A**) Schematic of the experimental protocol used for AAV-STITCH and mosaic capsid production. Plasmids encoding the AAV components were co-expressed together with a construct encoding the SpyCatcher fused to the anti-GD2 scFv tagged with V5 (αGD2-V5-SpyCatcher). In brief, all viruses were produced in HEK293 cells by packaging the CBh-ZsGreen transgene. For mosaic generation, plasmids encoding AAV9m (W503A) and AAV9m carrying the SpyTag insertion in region 4 (AAV9m-ST) were co-transfected at the following ratios: AAV9m-ST:AAV9m = 1:0, 1:3, and 1:7. (**B**) Western blot with anti-AAV antibody showing the capsid composition of SpyTag-modified and mosaic AAV preparations (VP = viral proteins; VP + ST = viral proteins carrying the SpyTag peptide). (**C**) On the left, Western blot using an anti-V5 antibody to assess the coupling rate of the αGD2-SC fusion to non-mosaic and mosaic (1:3 and 1:7; AAV9mut-ST:AAV9mut) AAV-STITCH αGD2 capsids. On the right, densitometric quantification of the bands and calculation of the average number of αGD2-SC molecules conjugated per viral capsid. *n* = 3 independent AAV productions. (**D**) Quantification by flow cytometry of ZsGreen positive SH-SY5Y cells transduced with control (untreated), AAV9m-ST and AAV9m-ST + αGD2-SC (non-mosaic 1:0 and mosaic 1:3–1:7). (**E**) Bar graph showing the quantification of ZsGreen+ cells measured by flow cytometry across the different experimental conditions. Data are presented as mean ± SD, with individual data points shown (*n* = 3 independent biological replicates). **P = 0.002: AAV9m-ST/AAV9m-αGD2 (1:3) vs AAV9m-ST; **P = 0.005: AAV9m-ST/AAV9m-αGD2 (1:3) vs AAV9m-ST/AAV9m-αGD2 (1:7); *P = 0.044: AAV9m-ST/AAV9m-αGD2 (1:3) vs AAV9m-ST-αGD2. One-way ANOVA with Tukey’s multiple comparisons correction. (**F**) On the right, quantification by flow cytometry of ZsGreen positive SH-SY5Y cells transduced with AAV9m-ST + αGFP-SC (non-mosaic 1:0 and mosaic 1:3). On the left, bar graph showing the quantification of ZsGreen+ cells measured by flow cytometry after transduction with AAV9m-ST + αGFP-SC (non-mosaic 1:0 and mosaic 1:3). Data are presented as mean ± SD, with individual data points shown (*n* = 3 independent biological replicates). (**G**) RT-qPCR quantification of cell membrane–bound viral genomes (4 °C assay) following incubation with control AAV9m-ST alone or conjugated with αGD2-SC non-mosaic 1:0 and mosaic 1:3–1:7. Data are presented as mean ± SD, with individual data points shown (*n* = 3 independent biological replicates). ***P = 0.0001: AAV9m-ST vs AAV9m-ST-αGD2; ***P = 0.0004: AAV9m-ST vs AAV9m-ST/AAV9m-αGD2 1:3. One-way ANOVA with Tukey’s multiple comparisons correction. (**H**) RT-qPCR quantification of viral genome copies in SH-SY5Y cells transduced with control AAV9mut-ST alone or conjugated with αGD2-SC non-mosaic 1:0 and mosaic 1:3–1:7. Data are presented as mean ± SD, with individual data points shown (*n* = 3 independent biological replicates). *P = 0.032: AAV9m-ST vs AAV9m-ST-αGD2; ***P = 0.0001: AAV9m-ST vs AAV9m-ST/AAV9m-αGD2 1:3. One-way ANOVA with Tukey’s multiple comparisons correction. Source data are available online for this figure.

**Figure EV1:**
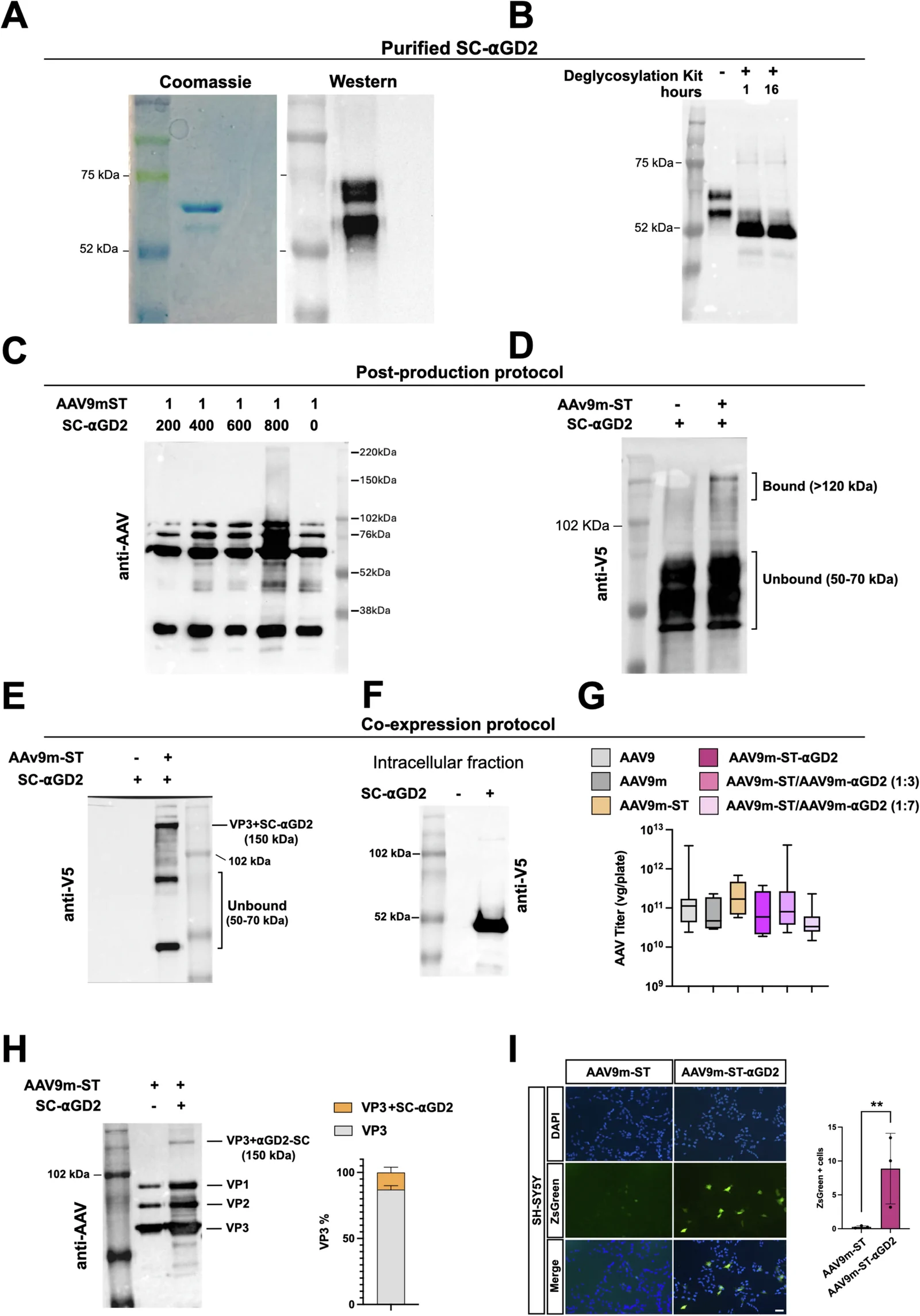
Optimization of AAV-STITCH production comparing post-production and co-expression protocols. (**A**) Representative Coomassie staining and western blot with anti-V5 antibody showing the purification of the anti-GD2 scFv protein (αGD2-SC) via His-tag affinity isolation. (**B**) Western blot with anti-V5 of purified αGD2-SC protein treated with deglycosylation enzymes for 1 and 16 h, demonstrating the effect of enzymatic deglycosylation. (**C**) Representative Western blot with anti-AAV showing the post-production coupling protocol, in which purified αGD2-SC was incubated with AAV9m-ST at different ratios (1:200, 1:400, 1:600, and 1:800). (**D**) Representative anti-V5 Western blot showing the detection of αGD2-SC fused to AAV9m-ST after post-production coupling protocol. (**E**) Western blot anti-V5 showing the detection of αGD2-SC fused to AAV9m-ST after co-expression coupling protocol. (**F**) Western blot with anti-V5 antibody showing the intracellular αGD2-SC band. (**G**) Box plot showing the viral genome titers (vg/plate), assessed by RT-qPCR, from multiple independent productions of AAV9, AAV9m, AAV9m-ST, and 1:3 and 1:7 mosaic preparations. AAV9: *n* = 12; minimum = 2.40 × 10¹⁰; Q1 (25%) = 4.12 × 10¹⁰; Median = 1.11 × 10¹¹; Q3 (75%) = 1.64 × 10¹¹; maximum = 3.91 × 10¹². AAV9m: *n* = 4; minimum = 2.89 × 10¹⁰; Q1 (25%) = 3.23 × 10¹⁰; Median = 4.71 × 10¹⁰; Q3 (75%) = 1.03 × 10¹¹; maximum = 2.30 × 10¹¹. AAV9m-ST: *n* = 4; minimum = 5.69 × 10¹⁰; Q1 (25%) = 7.33 × 10¹⁰; Median = 1.24 × 10¹¹; Q3 (75%) = 2.97 × 10¹¹; maximum = 6.82 × 10¹¹. AAV9m-STITCH: *n* = 6; minimum = 1.89 × 10¹⁰; Q1 (25%) = 2.60 × 10¹⁰; Median =  5.89 × 10¹⁰; Q3 (75%) = 1.97 × 10¹¹; maximum = 3.75 × 10¹¹. AAV9-STITCH 1:3: *n* = 18; minimum = 2.36 × 10¹⁰; Q1 (25%) = 4.08 × 10¹⁰; Median =  9.60 × 10¹⁰; Q3 (75%) = 2.39 × 10¹¹; maximum = 4.07 × 10¹². AAV9-STITCH 1:7: *n* = 7; minimum = 1.47 × 10¹⁰; Q1 (25%) = 2.69 × 10¹⁰; Median = 3.34×10¹⁰; Q3 (75%) = 6.08 × 10¹⁰; maximum = 2.30 × 10¹¹. (**H**) Left: Western blot anti-AAV showing the detection of αGD2-SC fused to AAV9m-ST after co-expression coupling protocol. Right: Densitometric quantification of the proportion of unconjugated VP3 (gray) and VP3 conjugated with αGD2-SC (orange). Data are presented as mean ± SD. (**I**) Left: immunofluorescence using anti-ZsGreen antibody on SH-SY5Y cells transduced with control AAV9m-ST alone or couple with αGD2-SC (co-expression coupling protocol). Scale bar:100um. Right: bar graph showing the quantification of ZsGreen+ cells over total cells (DAPI + ) in the different experimental conditions. Data are presented as mean ± SD, with individual data points shown (*n* = 3 independent biological replicates). **P = 0.0031 AAV9m-ST vs AAV9m-ST-αGD2-SC; *t* Test.

To establish an effective and modular approach for coupling antibody fragments to AAV capsids, we leveraged SpyTag technology. This system relies on two small reactive protein fragments, SpyTag and SpyCatcher, which spontaneously form a covalent isopeptide bond with high affinity (Zakeri et al, 2012). Given that the SpyTag fragment consists of only 13 amino acids, we hypothesized that its integration into AAV variable regions would pose a lower risk to destabilize the AAV structure. Thus, the SpyTag sequence, flanked by spacer elements, was cloned after amino acid 453 (the VP1 position) within the variable region IV of the AAV9 capsid, which has been shown to be an editable site in the capsid without altering the virus productivity and infectivity (Fig. 1A) (Chen et al, 2023). We selected the AAV9 capsid serotype for its high productivity yield. Moreover, we recently showed that the AAV-W503A mutant capsid (AAV9m), previously described to abolish its binding to galactose, is almost fully deficient in cellular infectivity (Giannelli et al, 2024). Thus, we reasoned that this AAV9m represents a perfect neutral packaging vector, whose tropism will be solely dictated by the peptides grafted onto its surface. Next, we fused the SpyCatcher domain to the GD2-specific scFv antibody fragment (14g2a) (a kind gift from Dr. M. Brenner), incorporating an optimized glycine-rich linker to provide increased structural flexibility, along with V5 and His tags for detection and purification purposes (Fig. 1A) (Pule et al, 2008). To conjugate the anti-GD2 scFv to the viral capsid, we compared two alternative production strategies. In the first approach (“post-production coupling”), AAV9m-ST (AAV9m with SpyTag) particles and the His-tag–purified SpyCatcher–αGD2 (SC-αGD2) protein were produced separately and then incubated together at different ratios. Interestingly, the SC-αGD2 protein recovered from the supernatant migrated at a higher apparent molecular weight than expected (~ 52 kDa), running between ~50–70 kDa (Fig. EV1A). This molecular weight shift was determined to result from glycosylation, as confirmed by deglycosylation assays (Fig. EV1B). However, this method proved largely inefficient: testing different ratios of SC-αGD2 to AAV, ranging from 1:200 to 1:800, revealed that coupling was detectable only at extremely high ratios (1:800), and a substantial amount of unconjugated SC-αGD2 remained even after filter purification, further highlighting the limitations of this post-production approach (Fig. EV1C,D). In the second approach (“co-expression coupling”), the plasmid encoding SC-αGD2 was co-transfected together with the AAV packaging plasmids in HEK293T cells (Fig. 1A). This strategy resulted in efficient covalent coupling and minimal unbound protein after iodixanol gradient purification (Fig. EV1E). Notably, the intracellular SC-αGD2 protein did not display glycosylation, indicating that the co-expression protocol avoids unwanted post-translational modifications associated with protein secretion (Fig. EV1F). Additionally, this approach bypasses the need for labor-intensive protein purification. Based on these results, we adopted the co-expression method for all subsequent experiments.

**Figure EV2:**
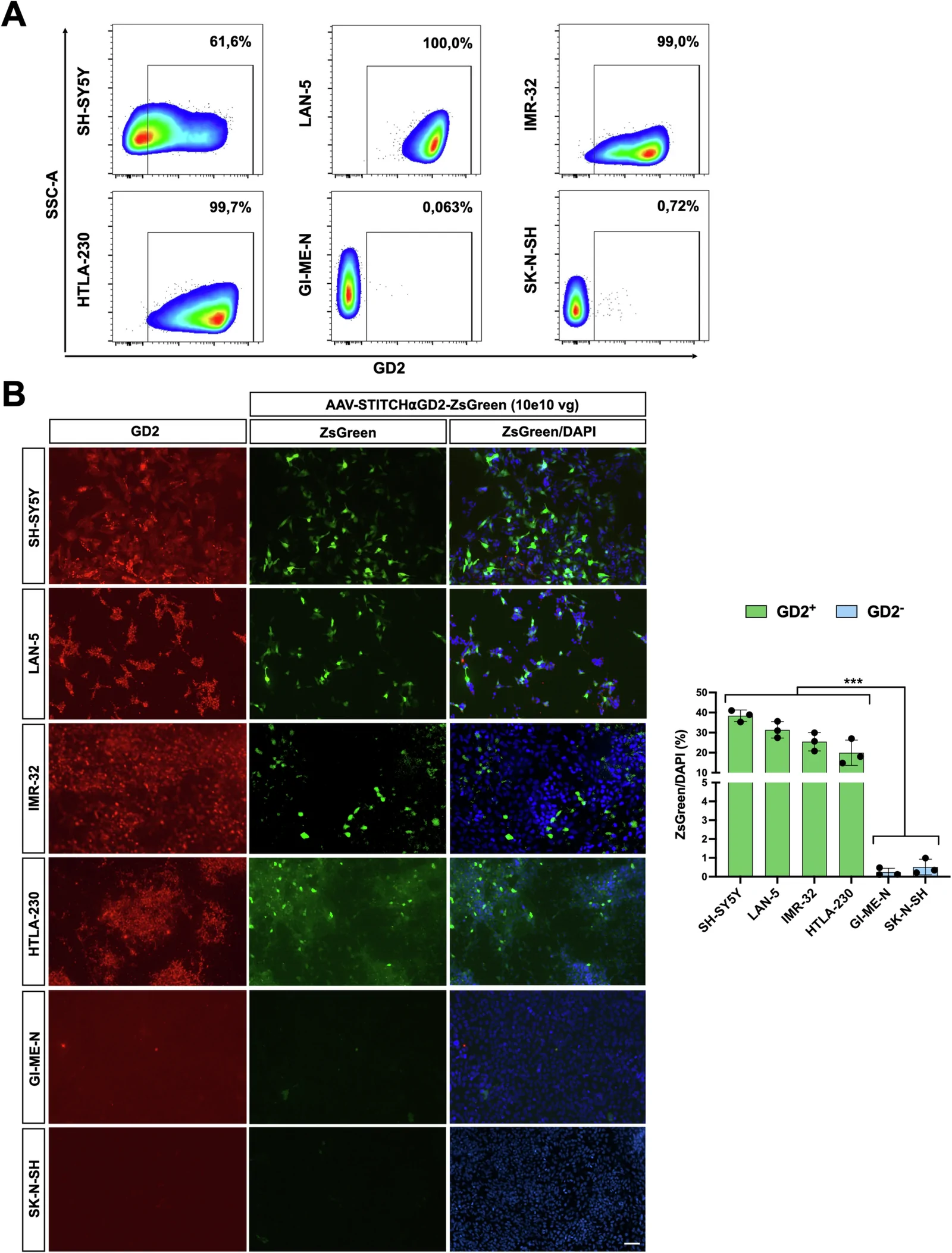
Specificity and efficiency of AAV-STITCHαGD2 transduction in neuroblastoma cell lines. (**A**) Quantification of GD2 expression in the various NB cell lines analyzed in this study by flow cytometry. (**B**) Representative immunofluorescence for anti-GD2 and anti-ZsGreen in GD2+ and GD2− NB cell lines and bar graph showing the quantification of ZsGreen+ cells over total cells in the different NB cell lines transduced with AAV-STITCHαGD2 carrying the ZsGreen transgene. Scale bar: 100 µm. Data are presented as mean ± SD, with individual data points shown (*n* = 3 independent biological replicates). ***P = 0.0001 SH-SY5Y vs GI-ME-N. ***P = 0.0001 SH-SY5Y vs SK-N-SH. ***P = 0.0001 LAN5 vs GI-ME-N. ***P = 0.0001 LAN5 vs SK-N-SH. ***P = 0.0001 IMR-32 vs GI-ME-N. ***P = 0.0001 IMR-32 vs SK-N-SH. ***P = 0.0004 HTLA-230 vs GI-ME-N. ***P = 0.0004 HTLA-230 vs SK-N-SH. One-way ANOVA with Tukey’s multiple comparisons correction.

**Figure EV3:**
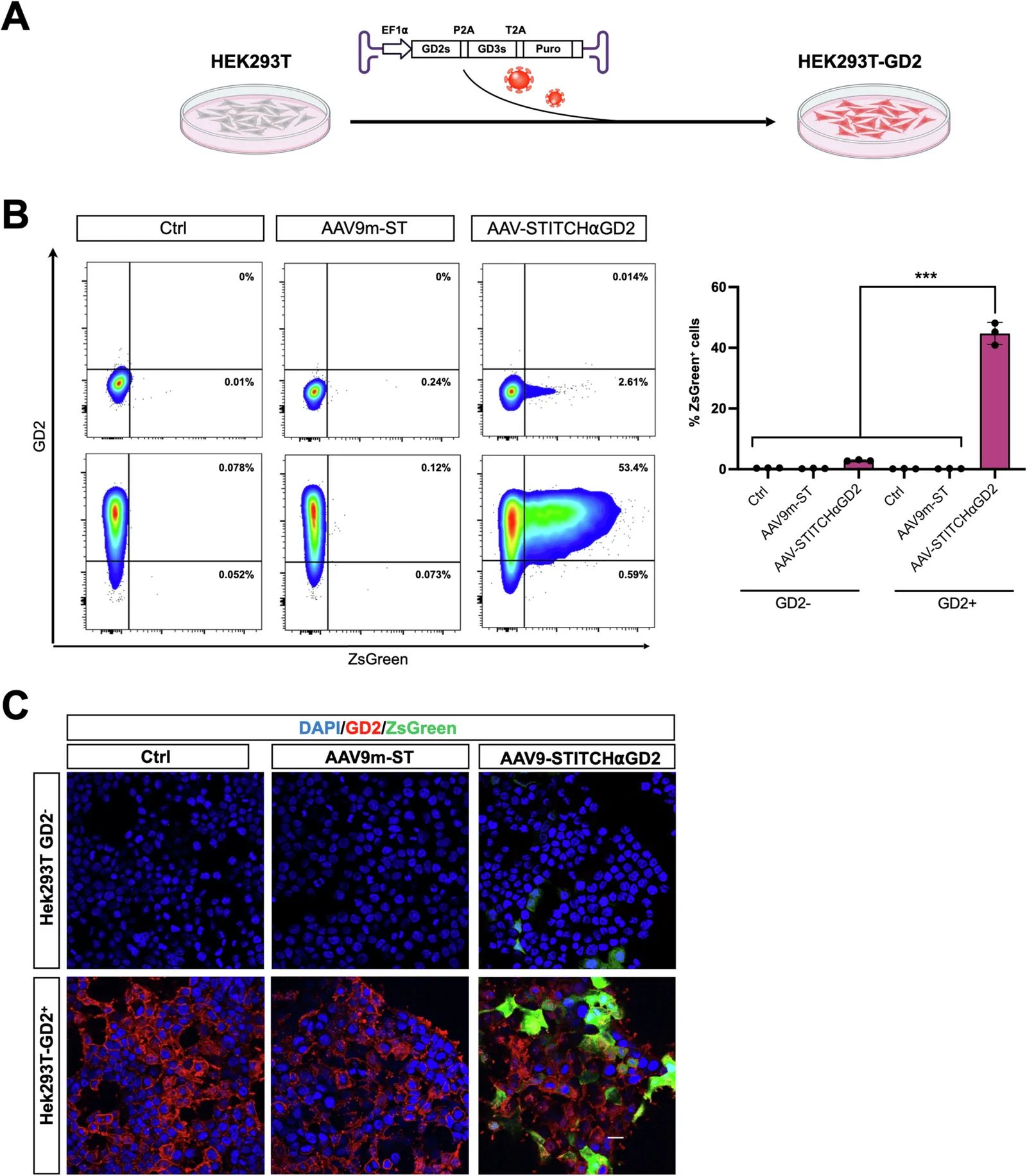
Specificity and efficiency of AAV-STITCHαGD2 transduction in HEK293T cells engineered to express GD2. (**A**) Illustration of the experimental protocol. HEK293T cells were transduced with a multicistronic lentiviral vector containing the coding sequences (CDS) of GD2 synthase (GD2S), GD3 synthase (GD3S), and a puromycin resistance gene for selection, for generating HEK293-GD2+ cells. (**B**) Left: Flow cytometry analysis of ZsGreen+ (*X* axis) and GD2+ (*Y* axis) cells in HEK293-GD2− and HEK293-GD2+ cells following transduction with AAV-STITCHαGD2 or AAV9m-ST carrying the ZsGreen transgene. Right: Bar graph showing the percentage of ZsGreen+ cells over total cells in HEK293-GD2− and HEK293-GD2+ after transduction. Data are presented as mean ± SD, with individual data points shown (*n* = 3 independent biological replicates). ***P = 0.0001 GD2 + AAV-STITCHαGD2 vs GD2- Ctrl; ***P = 0.0001 GD2 + AAV-STITCHαGD2 vs GD2- AAV9m-ST; ***P = 0.0001 GD2 + AAV-STITCHαGD2 vs GD2- AAV-STITCHαGD2; ***P = 0.0001 GD2 + AAV-STITCHαGD2 vs GD2+ Ctrl; ***P = 0.0001 GD2 + AAV-STITCHαGD2 vs GD2 + AAV9m-ST. One-way ANOVA with Tukey’s multiple comparisons correction. (**C**) Representative immunofluorescence images using anti-ZsGreen and anti-GD2 antibodies in HEK293-GD2− and HEK293-GD2+ cells transduced with AAV-STITCHαGD2 or AAV9m-ST. Scale bar: 100 μm.

**Figure EV4:**
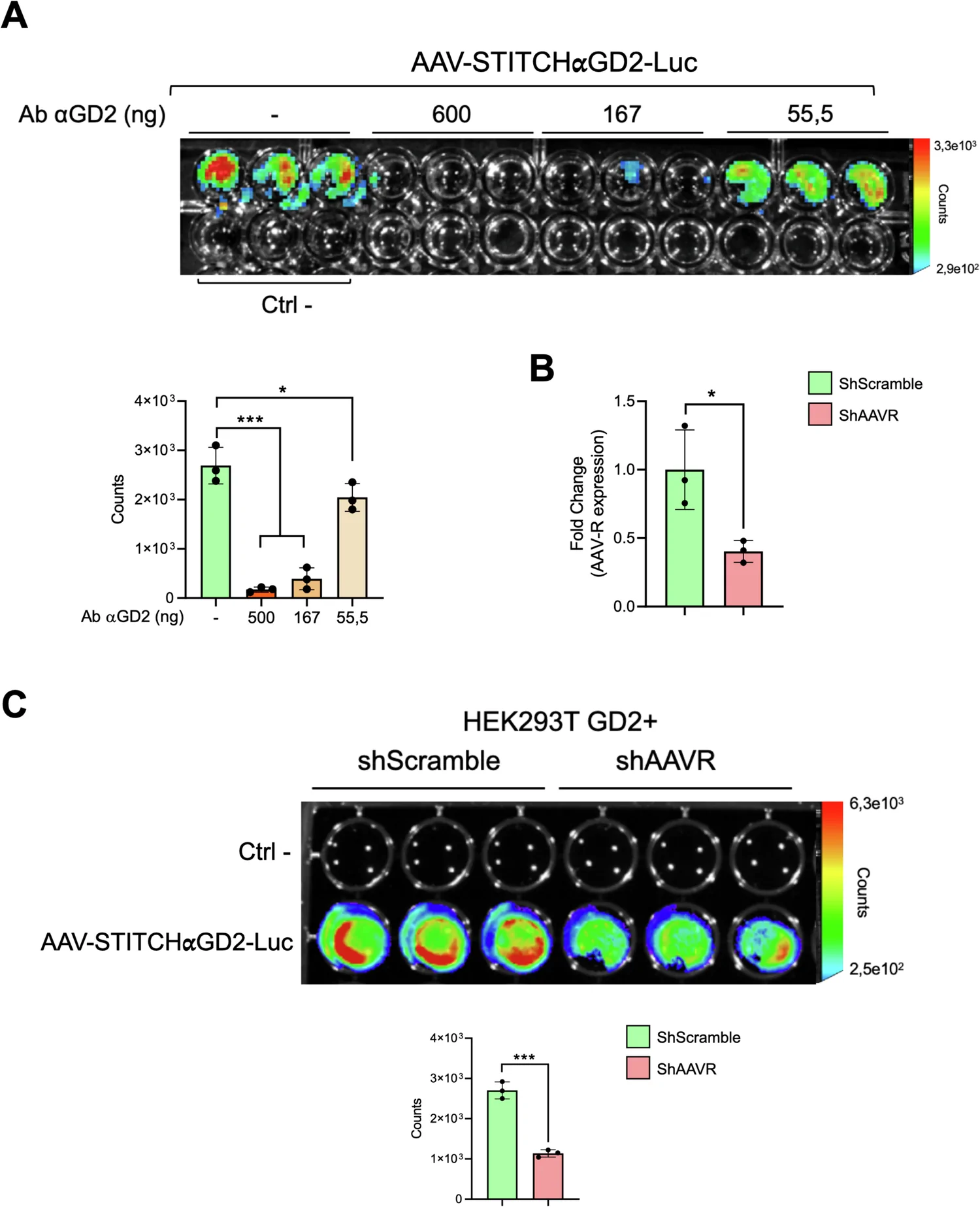
Anti-GD2 competition assay and AAVR knockdown analysis. (**A**) Representative IVIS images of HEK293-GD2+ cells transduced with AAV-STITCHαGD2 carrying a CMV-Luc reporter cassette following pre-incubation with anti-GD2 antibody at 5 µg/mL, 1.67 µg/mL, or 0.56 µg/mL. The lower row shows non-transduced control cells. Left, bar graph showing quantification of luminescence signal (counts) in cells pre-incubated or not with anti-GD2 antibody. Data are presented as mean ± SD, with individual data points shown (*n* = 3 independent biological replicates). ***P = 0.0001 – vs 500; ***P = 0.0001 – vs 167; *P = 0.0379 – vs 55.5. One-way ANOVA with Tukey’s multiple comparisons correction. (**B**) qRT-PCR analysis showing AAVR silencing in HEK293-GD2+ cells transfected with an shRNA targeting AAVR compared with scramble shRNA control. Gene expression levels were normalized to 18S rRNA. Data are presented as mean ± SD, with individual data points shown (*n* = 3 independent biological replicates). *P = 0.0265 ShScramble vs ShAAVR; *t* Test. (**C**) Representative IVIS images of HEK293-GD2+ cells transduced with AAV-STITCHαGD2 carrying a CMV-Luc reporter cassette following transfection with either anti-AAVR shRNA or scramble control. The upper row shows non-transduced control cells. Right, bar graph showing quantification of luminescence signal (counts) in cells treated with anti-AAVR shRNA compared with scramble control. Data are presented as mean ± SD, with individual data points shown (*n* = 3 independent biological replicates). ***P = 0.0003 ShScramble vs ShAAVR; *t* Test.

We opted to produce self-complementary AAVs since they assure a faster and higher transgene expression which is crucial when targeting dividing cancer cells (McCarty 2008). Multiple independent virus productions revealed that the viral titer remained consistent, indicating that the co-expression protocol does not compromise virus yield (Fig. EV1G). To investigate the incorporation of the SC-αGD2 into the capsid, we performed Western blot analysis using an antibody specific for AAV capsid proteins. We observed that SC-αGD2 predominantly bound to the VP3 protein (Fig. 1B,C). Densitometric quantification of the Western blot signals revealed that approximately 13 ± 3% of VP3 subunits were conjugated to SC-αGD2. Given that each AAV capsid contains ~50 VP3 molecules, we estimated that an average of 7–8 SC-αGD2 molecules were conjugated per capsid (Fig. EV1H). When testing transduction efficiency in GD2 + SHSY-5Y NB cells, we found that the unconjugated AAV9m-ST was unable to transduce the cells (Fig. EV1I). In contrast, the antibody-conjugated AAV was able to mediate transduction, albeit with an efficiency below 10%. (Fig. EV1H). We hypothesized that the high density of SC-αGD2 molecules on the viral surface might create steric hindrance, interfering with the interaction between the AAV capsid and essential co-receptors required for cellular entry. To test this hypothesis, we applied the same production protocol to generate mosaic capsids by mixing Rep-Cap plasmids containing or lacking the SpyTag sequence at different ratios (1:3 and 1:7) (Fig. 1A,B). This approach effectively reduced the number of SC-αGD2 molecules conjugated to VP3 subunits from ~7–8 in the non-mosaic condition to 2–3 in the 1:3 ratio, and to fewer than 1 in the 1:7 ratio, based on Western blot quantification for V5 (Fig. 1C). To determine these values, we first estimated the number of SC-αGD2 molecules in the non-mosaic condition (set as ~7 copies) (Fig. EV1H) and then normalized the band intensities in mosaic conditions accordingly (Fig. 1C). We then tested the transduction efficiency of the mosaic capsids in vitro and found that the 1:3 condition resulted in the highest transduction rate with more than 6-fold increase compared to the non-mosaic capsids (Fig. 1D,E). Importantly, this effect was highly specific, as replacing the targeting GD2-scFv with an unrelated anti-GFP scFv completely abolished transduction of SH-SY5Y cells (Fig. 1F). To assess viral binding independently of internalization, we performed a binding assay at 4 °C, a condition under which viral entry is blocked, and observed that mosaic capsids exhibited reduced binding to the membrane of SH-SY5Y cells (Fig. 1G). However, when analyzing viral vector copy number in cells following transduction at 37 °C, we found that the 1:3 mosaic capsids resulted in a higher number of viral genomes per cell (Fig. 1H), consistent with the increased expression of the ZsGreen reporter observed by immunofluorescence. Based on these findings, we selected the 1:3 mosaic capsid configuration and designated it as AAV-STITCHαGD2. We then expanded its characterization to further evaluate its specificity and transduction efficiency across different NB cell lines, that were validated for GD2 expression by flow cytometry (Fig. EV2A). Notably, the AAV-STITCHαGD2 efficiently transduced GD2 + NB cancer cell lines, such as SH-SY5Y, LAN-5, IMR-42 and HTLA-230, as shown by expression of the ZsGreen viral transgene (Fig. EV2B). Conversely, GI-ME-N and SK-N-SH NB tumor cells, which are negative for GD2, resulted resistant to AAV-STITCHαGD2 transduction (Fig. EV2B). To further validate the specificity of this capsid conjugation, we decided to test the AAV-STITCHαGD2 transduction profile in two populations of the same cell type, with or without GD2. For this scope, GD2S and GD3S, the two enzymes for the GD2 synthesis, were stably expressed by lentiviral transduction in HEK293T cells that normally have minimal or no content of this glycolipid (Fig. EV3A). Notably, AAV-STITCHαGD2 was able to transduce only GD2 + HEK293T cells (Fig. EV3B,C), confirming its selective dependency by this antigen. Moreover, we exploited this highly controlled cellular model to further corroborate specificity through an antibody competition assay using the AAV-STITCHαGD2 vector encoding luciferase. In this assay, antibody-mediated competition of GD2 binding was evaluated by pre-incubating GD2-expressing cells with increasing amounts of an anti-GD2 antibody. This treatment significantly reduced transduction in a dose-dependent manner, as measured by luciferase activity, further confirming the antigen-dependent mechanism of cell targeting (Fig. EV4A). We then investigated the role of AAV-R in viral entry and demonstrated that this host receptor is essential for efficient transduction. Indeed, shRNA-mediated knockdown of AAV-R, which resulted in a ~ 60% reduction in transcript levels, as confirmed by qRT-PCR (Fig. EV4B), markedly impaired transduction by the GD2-targeting AAV-STITCH vector (Fig. EV4C). These findings indicate that, while GD2 mediates initial cell attachment, AAV-R is required for productive viral internalization.

**Figure EV5:**
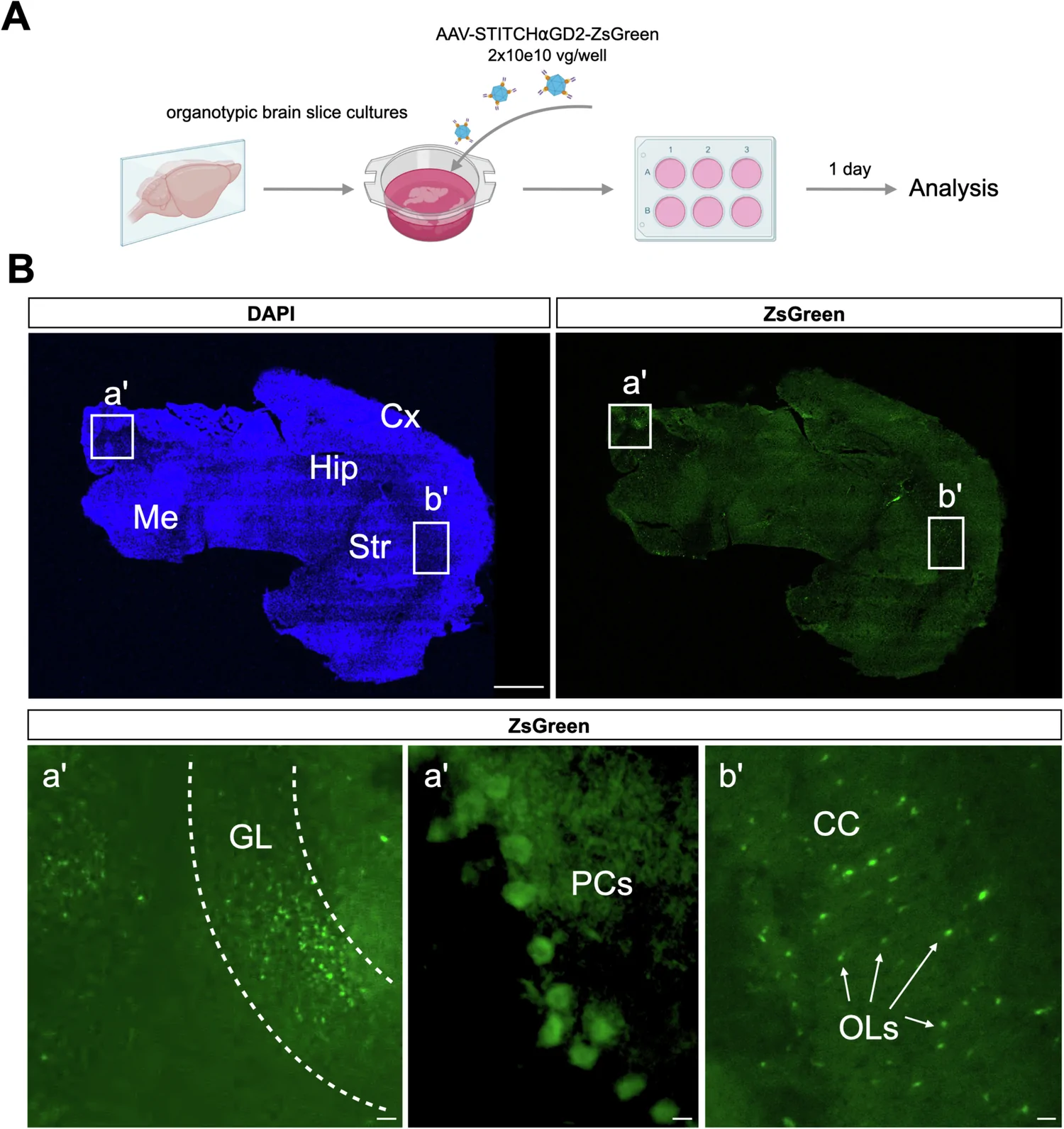
Transduction profile of AAV-STITCHαGD2 in organotypic murine brain slices. (**A**) Illustration of the experimental protocol. Murine organotypic brain slices were infected with 2 × 10¹⁰ vg/well of AAV-STITCHαGD2 carrying the ZsGreen transgene. The following day, slices were processed and the transduction profile was analyzed by immunofluorescence staining. (**B**) Representative immunofluorescence images of a sagittal brain section transduced with AAV-STITCHαGD2, showing DAPI staining on the left and anti-ZsGreen staining on the right. Scale bar: 1000 µm. (**a′**) Left: Representative higher magnification image of the cerebellum, showing transduced cells in the granular layer (GL). Scale bar: 100 µm. Right: Higher magnification image of cerebellar Purkinje cells (PCs). Scale bar: 100 µm. (**b′**) Representative higher magnification image showing transduced oligodendrocytes (OLs) in the corpus callosum (CC). Scale bar: 100 µm.

**Figure EV6:**
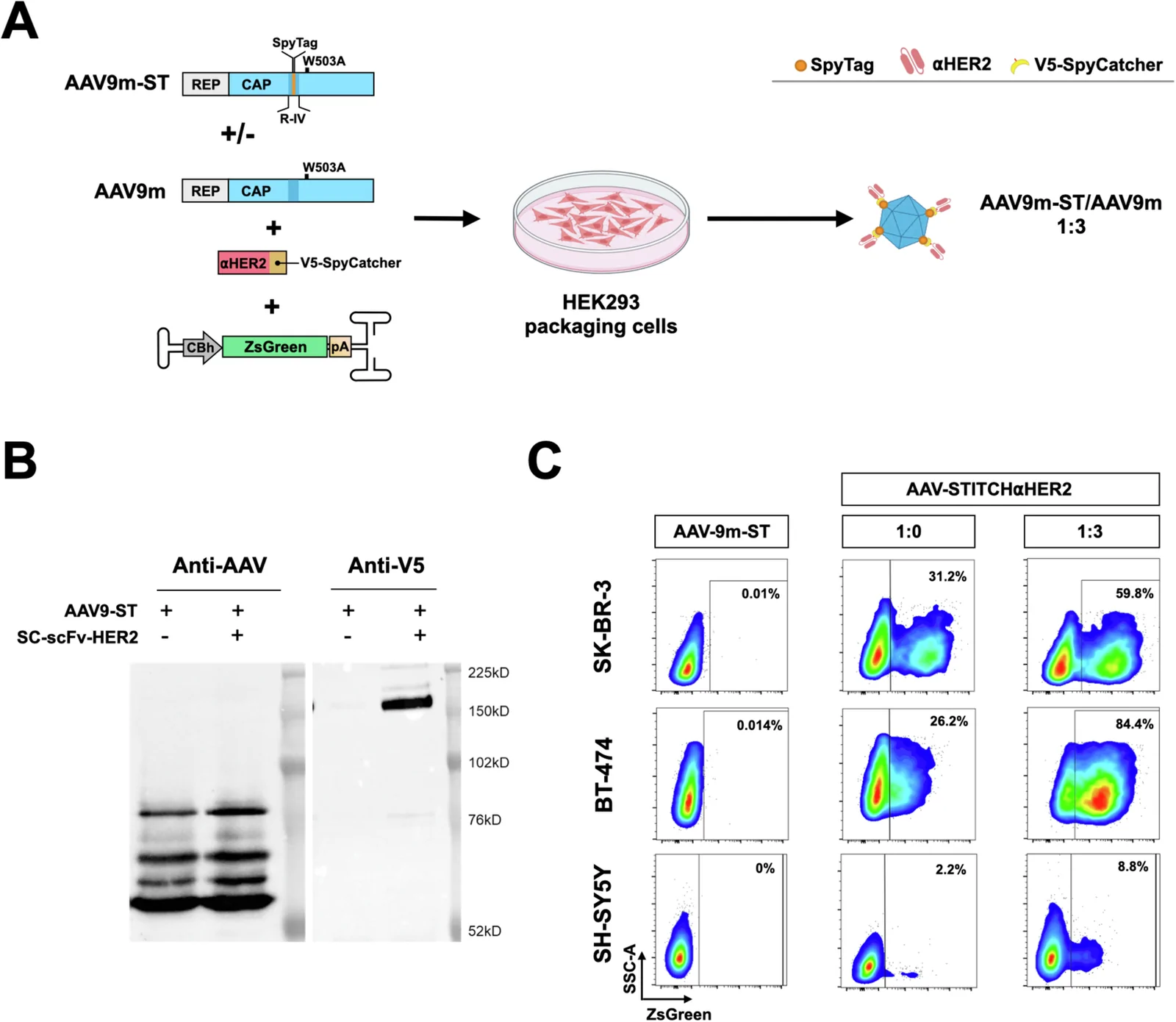
Production and functional validation of AAV-STITCHαHER2 vectors. (**A**) Schematic of the experimental protocol used for AAV-STITCHαHER2 capsid production. Plasmids encoding the AAV components were co-expressed together with a construct encoding the SpyCatcher fused to the anti-HER2 scFv tagged with V5 (αHER2-V5-SpyCatcher). In brief, AAV particles were produced in HEK293 cells by packaging the CBh-ZsGreen transgene and by mixing the AAV9m-ST and AAV9m with ratio: 1:3. (**B**) Western blot with anti-AAV and anti-V5 antibodies showing the capsid composition of AAV-STITCHαHER2 comparing to AAV9m-ST mosaic capsid. (**C**) Quantification by flow cytometry of ZsGreen positive SH-SY5Y (HER2-), SK-BR-3 (HER2 + ) and BT-474 (HER2 + ) cell lines after transduction with AAV-STITCHαHER2 1:0 and 1:3 with respect the untreated control.

We assumed that beyond its therapeutic potential, the AAV-STITCH system can also serve as a powerful tool to map the spatial distribution of antigens that are difficult to detect by conventional immunohistochemistry, such as GD2. In fact, despite its clinical relevance, GD2 expression remains poorly characterized, due to the lack of suitable antibodies for in vivo studies, with only limited evidence on its presence in select cell types within the adult brain (Lammie et al, 1993; Marconi et al, 2005). To address this gap, we leveraged the ZsGreen-expressing AAV-STITCHαGD2 system to trace GD2 distribution in mouse brain organotypic slice cultures, as the mutant AAV9 vector is unable to cross the blood–brain barrier (Fig. EV5A). Viral transduction was observed in discrete neural cell types, including the granular and Purkinje cells of the cerebellum, as well as oligodendrocytes in the corpus callosum, cell populations previously suggested to express GD2 (Fig. EV5B) (Lammie et al, 1993; Marconi et al, 2005). These findings provide the first evidence that the αGD2 moiety is sufficient to redirect viral tropism, enabling specific transduction of both healthy and malignant GD2⁺ neural cells. To confirm the versatility and broad applicability of our approach, we generated an AAV-STITCH using a selective scFv against HER2. HER2 (human epidermal growth factor receptor 2) is often amplified with resulting overexpression in a variety of solid malignancies including breast, stomach, and esophageal cancers and is the target of effective anti-cancer drugs (Swain et al, 2023). We confirmed that the HER2 scFv can be easily coupled to the capsid through the SpyTag/Catcher elements (Fig. EV6A,B). Importantly, we applied the same 1:3 mosaic configuration previously used for αGD2, thereby limiting the number of anchor sites on the capsid and reducing antibody binding. AAV-STITCHαHER2 mediated robust gene transfer in the breast cancer cell lines BT-474 and SK-BR-3, both characterized by high HER2 expression (Tobin et al, 2018), whereas the non-mosaic (1:0) configuration consistently showed lower transduction efficiency, further supporting that increasing the degree of coupling enhances overall gene transfer efficiency (Fig. EV6C).

In contrast, SH-SY5Y that do not express HER2 were resistant to the AAV-STITCHαHER2 infection (Fig. EV6C) (Ambrosetti et al, 2021). These findings clearly outline that the AAV-STITCH is a versatile platform that can easily allow the coupling of antibodies or other biologics to tailor viral tropism toward a desired cellular target.

### AAV-STITCHαGD2 show selective infectivity of NB tumor cells in vivo

**Figure 2:**
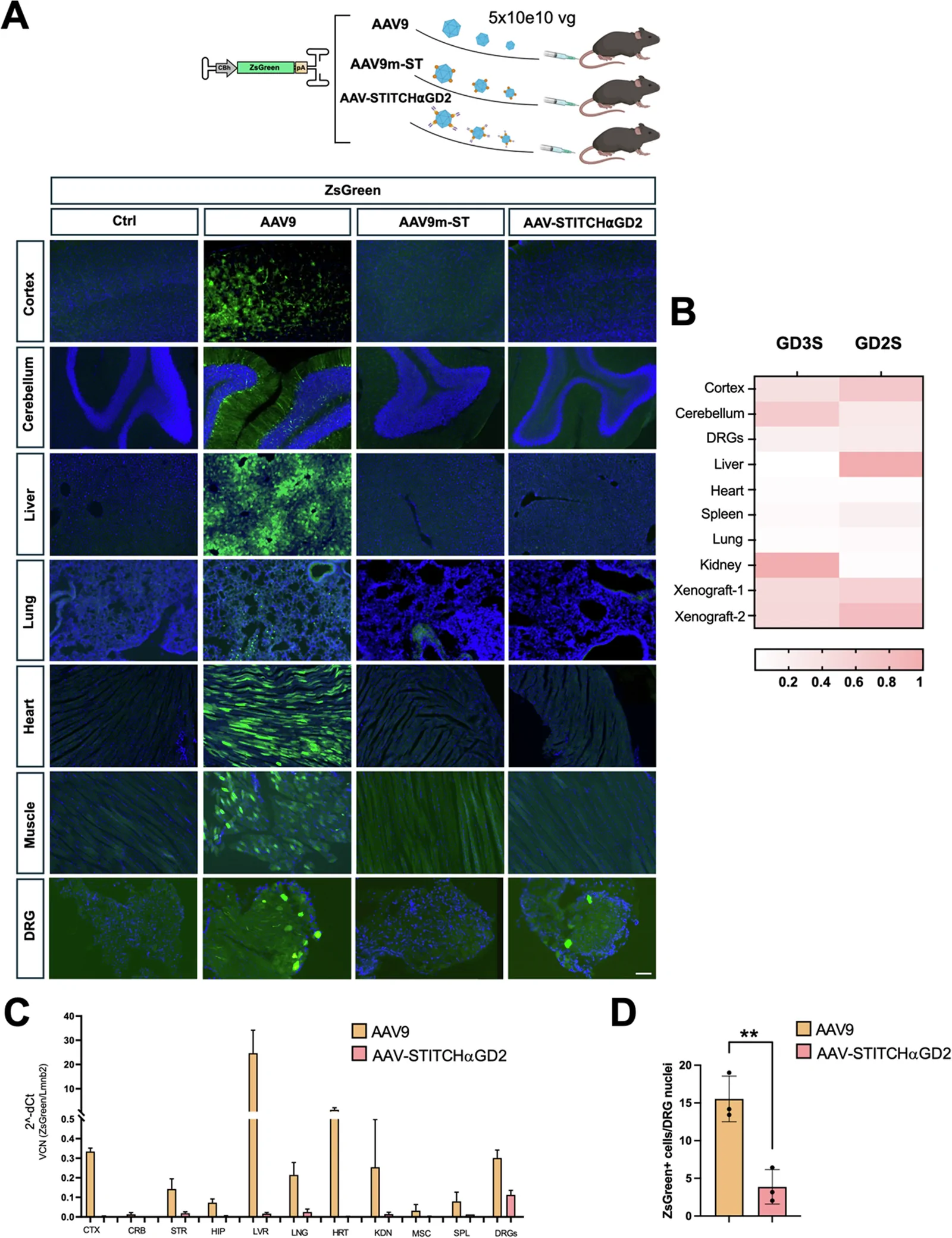
Biodistribution of AAV-STITCHαGD2 following systemic delivery in mice. (**A**) Top: Schematic representation of the experimental protocol. Briefly, biodistribution of AAV-STITCHαGD2 was assessed by ZsGreen transgene visualization following systemic injection in C57BL/6 mice, Control groups include non-injected mice or inoculated with wild-type AAV9 and the AAV9m-ST. Bottom: Representative immunofluorescence images showing ZsGreen expression, detected with an anti-ZsGreen antibody, in various organs to evaluate AAV-mediated transduction. Scale bar: 100 µm. *n* = 3 mice per group. (**B**) Heatmap showing relative expression levels of GD2S and GD3S across various organs and tissues. Gene expression was quantified by qRT-PCR and normalized using the 2^-ΔCt method. For each gene, values were further normalized by setting the highest expression value to 1, and scaling all other values accordingly, enabling comparative visualization of expression patterns. Color intensity reflects the relative normalized expression level of each enzyme in the respective tissue. (**C**) Quantification of the viral DNA transgene in different tissues by qPCR. Data are plotted as 2^ΔCt values comparing AAV9 and AAV-STITCHαGD2. Data are presented as mean ± SD (*n* = 3 mice per group). (**D**) Bar graph showing the quantification of ZsGreen+ cells on total DRG nuclei across the different experimental conditions. Data are presented as mean ± SD, with individual data points shown (*n* = 3 mice per group). **P = 0.006 AAV9 vs AAV-STITCHaGD2. *t* Test. Source data are available online for this figure.

**Figure 3:**
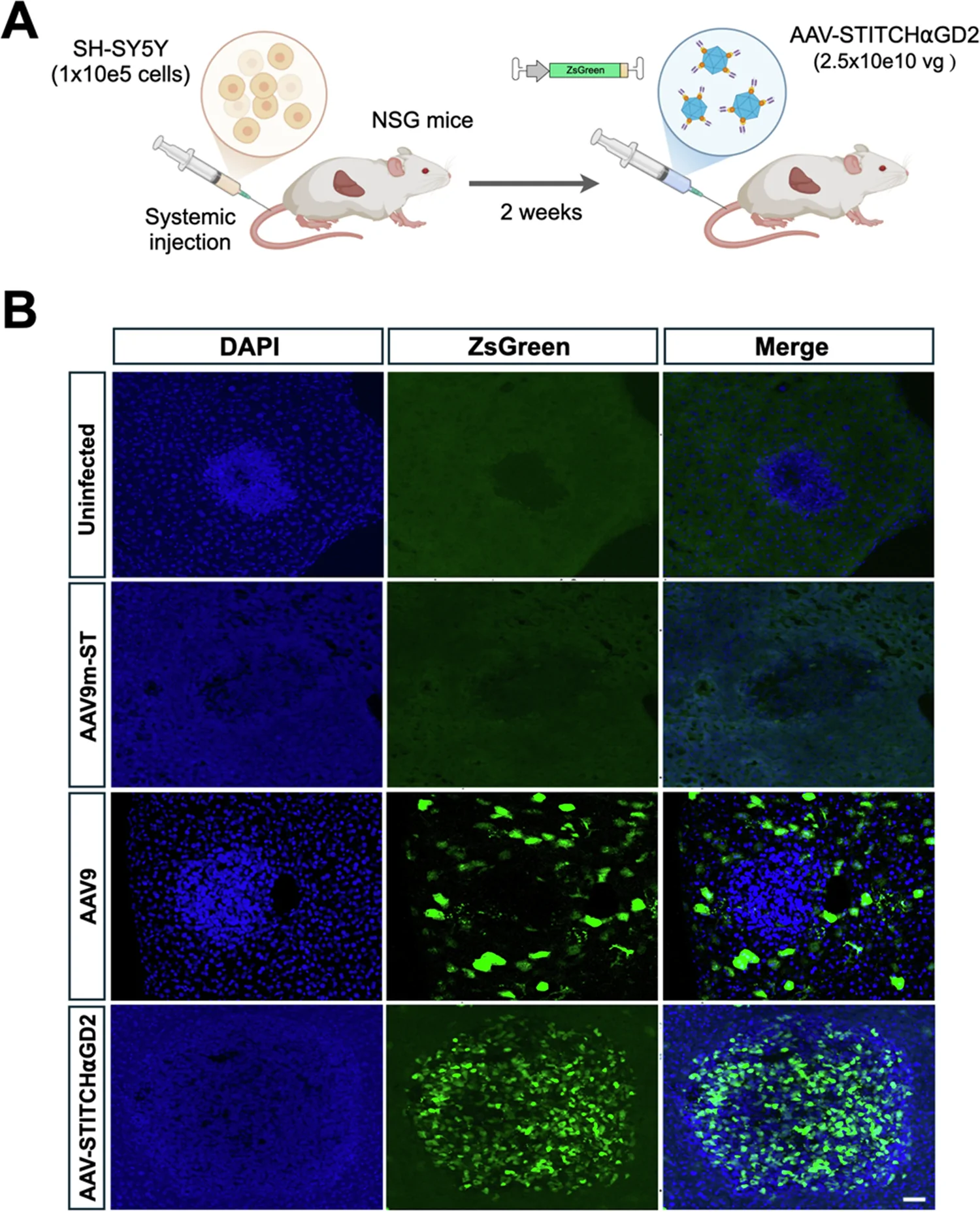
AAV-STITCH αGD2 targets liver metastasis in pseudometastatic neuroblastoma mouse model. (**A**) Illustration of the experimental protocol. Adult NGS mice were injected with 2 × 10^5^ SH-SY5Y cells through the tail vein (pseudometastatic model). Two weeks later, AAV-STITCHαGD2 or AAV9m-ST as control carrying ZsGreen transgene were inoculated in the tail vein using 5 × 10^10^ vg/mouse. Finally, treated mice were sacrificed three days after viral injection. (**B**) Anti-ZsGreen antibody immunostainings in liver of pseudometastatic mice untreated or treated with AV-STITCHαGD2, AAV-ST or AAV9 expressing ZsGreen transgene. Scale bar: 100 µm. *n* = 3 mice per group.

To stringently assess the transduction profile of the AAV-STITCHαGD2 in healthy tissues, we inoculated a viral dose of 5 × 10e10 vg in adult wild-type C57BL/6 mice and analyzed the expression of the viral gene in many tissues normally infected by the native AAV9 serotype. Notably, ZsGreen fluorescence was undetectable in all tissues that normally are efficiently transduced by AAV9 including liver, heart, muscles, brain and lungs (Fig. 2A). Failed transduction was also observed with the AAV9m-ST capsid, confirming the impaired infectivity resulting from the targeted AAV-W503A mutation, and αGD2 coupling did not mitigate this effect (Fig. 2A). The only notable exception was the dorsal root ganglia (DRG) neurons, which were partially transduced by AAV-STITCHαGD2, a finding consistent with previous reports describing GD2 expression levels in this neuronal population (Fig. 2A) (Lammie et al, 1993; Marconi et al, 2005). Furthermore, among peripheral tissues, only the DRG express both GD2-synthesizing enzymes, corroborating the in vivo specificity of the AAV-STITCHαGD2 transduction pattern. (Fig. 2B). ZsGreen immunofluorescence data were corroborated by qPCR-based viral genome copy analysis, that detected viral DNA only in DRG in animals inoculated with the AAV-STITCHαGD2, although at lower levels compared to AAV9 (Fig. 2C). Consistently, DRG neurons transduced by AAV-STITCHαGD2 were approximately one-third of those targeted with standard AAV9 at the same viral dose (16% ± 5% and 6% ± 2% for AAV9 and AAV-STITCHαGD2, respectively) (Fig. 2D). Next, we established a pseudometastatic NB xenograft mouse model, by intravascular inoculation of GD2 + SH-SY5Y tumor cells in NSG mice (Fig. 3A). This model rapidly developed NB metastases, with a notable enrichment in the liver within a few weeks (Pastorino et al, 2010). To evaluate the specific targeting efficiency of AAV-STITCHαGD2 in neuroblastoma (NB) tumor cells, NSG immunodeficient mice were first inoculated with SH-SY5Y cells. Two weeks after tumor cell injection, when the presence of visible, but small metastatic lesions was confirmed by magnetic resonance imaging, mice were administered either with AAV-STITCHαGD2 or control vectors, including the non-mutated AAV9m-ST and natural AAV9. Four days later, livers were then isolated and analyzed for the viral fluorescence signal. As shown in Fig. 3B, metastatic tissue was strongly positive to viral ZsGreen fluorescence. Notably, single NB tumor cells were successfully targeted by AAV-STITCHαGD2, while the surrounding healthy hepatic parenchyma remained almost completely untransduced (Fig. 3B). In contrast, wild-type AAV9 preferentially transduced hepatocytes within the liver parenchyma, with minimal or no detectable signal in metastatic foci. While the control AAV9m-ST vector failed to show detectable transduction in either tumor or healthy tissue (Fig. 3B). Thus, AAV-STITCHαGD2 acquired a very specific tropism for NB tumor cells sparing the infection of healthy tissue in the liver.

### TK-expressing AAV-STITCHαGD2 has strong anti-tumoral effects with undetectable adverse effects

**Figure EV7:**
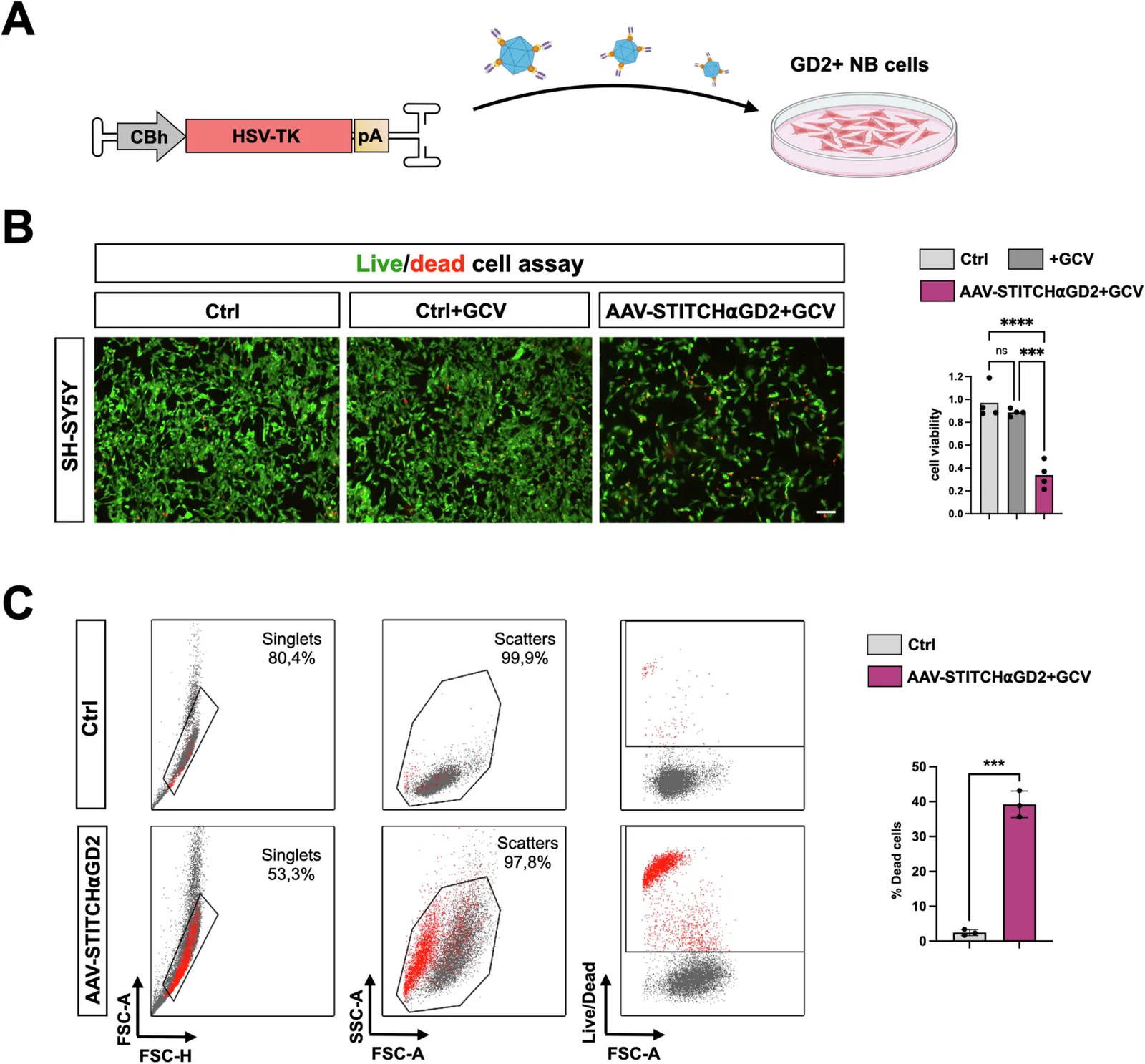
Evaluation of HSV-TK/GCV-mediated cytotoxicity in GD2+ neuroblastoma cells. (**A**) Schematic of the experimental protocol used to assess HSV-TK activity in GD2 + SH-SY5Y NB cells. (**B**) Left: Immunofluorescence images using the Live/Dead Assay Kit to evaluate cell death in SH-SY5Y cells transduced with AAV-STITCHαGD2 and treated with Ganciclovir (GCV), compared to non-infected controls with or without GCV treatment. Scale bar: 100 µm. Right: Bar graph showing quantification of cell viability across the three experimental conditions. Data are presented as mean ± SD, with individual data points shown (*n* = 4 independent biological replicates). ***P = 0.0001 Ctrl vs AAV-STITCHαGD2 + GCV; ***P = 0.0008 + GCV vs AAV-STITCHαGD2 + GCV. One-way ANOVA with Tukey’s multiple comparisons correction. (**C**) Left: Flow cytometry analysis of cell populations in control (Ctrl) and AAV-STITCHαGD2-treated samples. Each row represents a different condition: Ctrl (top) and AAV-STITCHαGD2 (bottom). Right: Bar graph showing the percentage of dead cells over total after transduction with AAV-STITCHαGD2 + GCV with respect the untreated control. Data are presented as mean ± SD, with individual data points shown (*n* = 3 independent biological replicates). ***P = 0.0001 Ctrl vs AAV-STITCHαGD2 + GCV. One-way ANOVA with Tukey’s multiple comparisons correction.

**Figure 4:**
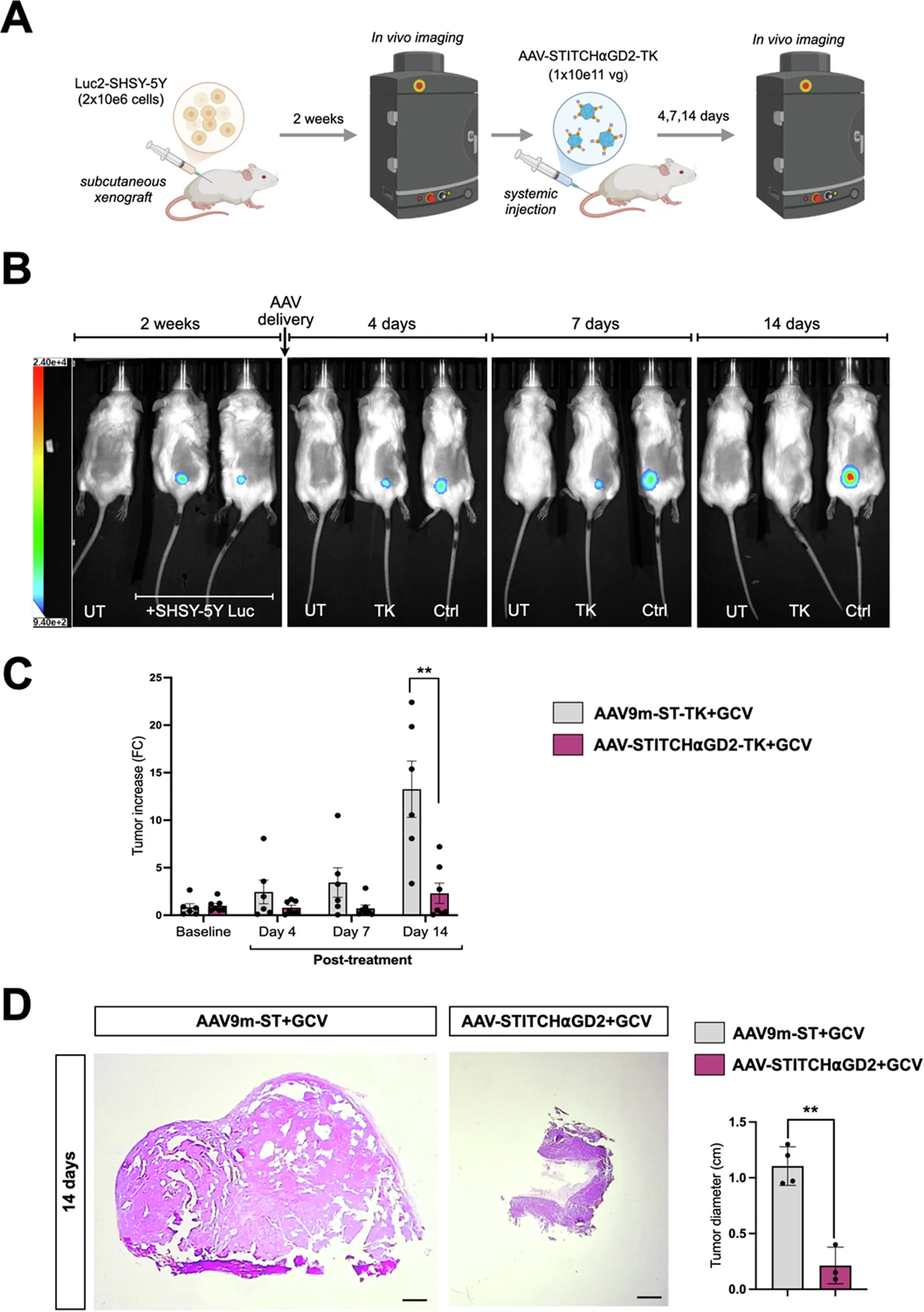
TK-expressing AAV-STITCHαGD2 inhibits the growth of subcutaneous tumor in xenograft mouse model. (**A**) Scheme of the experimental protocol. Adult NGS mice were subcutaneously injected with 1 × 10^6^ SH-SY5Y Luc+ cells (xenograft model). Two weeks later, tumoral masses were determined by IVIS scan and AAV-STITCHαGD2 or AAV9m-ST (negative control) carrying HSV-TK transgene were inoculated in the tail vein using 5 × 10^10^ vg/mouse. The following week, mice were daily injected with GCV and tumor growth was monitored by IVIS scan for 14 days. (**B**) Representative bioluminescence images taken with IVIS Lumina of control and TK treated xenograft mice acquired at different experimental time-points. (**C**) Bar plot showing tumor growth quantified by bioluminescence imaging, measured as the signal within a region of interest (ROI) drawn over the tumor, at baseline and 4, 7, and 14 days post-treatment in mice treated with AAV-STITCHαGD2 + GCV (*n* = 7 mice, purple) or AAV9m-ST + GCV (*n* = 6 mice, gray). Data were normalized to baseline and are presented as mean ± SD, with individual data points shown. **P = 0.0078 Day 14 AAV9m-ST-TK + GCV vs AAV-STITCHαGD2-TK + GCV. Two-way ANOVA with Sidak’s multiple comparisons correction. (**D**) Representative hematoxylin and eosin (H&E) staining of subcutaneous tumors from control (AAV-ST) and AAV-STITCHαGD2 mice with relative quantification. Scale bar: 1 mm. *n* = 4 independent biological replicates for AAV9m-ST + GCV and *n* = 3 independent biological replicates for AAV-STITCHαGD2 + GCV. Data are presented as mean ± SD, with individual data points shown. **P = 0.008 AAV9m-ST + GCV vs AAV-STITCHαGD2 + GCV. *t* Test. Source data are available online for this figure.

We then packaged a modified Herpes Simplex Virus thymidine kinase gene (HSV-TK A168H, hereafter referred to as TK), previously shown to enhance the efficacy of the HSV-TK/GCV suicide system (Oishi et al, 2022), into AAV-STITCHαGD2 to induce irreversible apoptotic cell death through the conversion of ganciclovir (GCV) into a toxic metabolite selectively in proliferating cells (Fig. EV7A). High cell death rate was confirmed on in vitro assays with transduced SH-SY5Y cells as revealed by both immunohistochemical and flow cytometry assays (Fig. EV7B,C). To enable convenient monitoring of AAV-STITCHαGD2-TK effects, we generated SH-SY5Y cells stably expressing Luc2, allowing longitudinal intravital bioluminescence imaging of subcutaneous xenografts (Fig. 4A). Next, we transplanted 2 × 10e6 Luc2-SH-SY5Y cells into the flank of NSG mice and 2 weeks later, animals were selected for comparable luciferase signal and divided into two groups which received a viral dose of 1 × 10e11 vg of either AAV-STITCHαGD2-TK or the control vector AAV9m-ST-TK (Fig. 4A). From the day following viral administration, mice were treated with ganciclovir (GCV). Notably, AAV-STITCHαGD2-TK robustly suppressed the growth of subcutaneous xenografts compared to the mock condition, as demonstrated by bioluminescence signal measurements over the 14-day observation period (Fig. 4B,C).

**Figure EV8:**
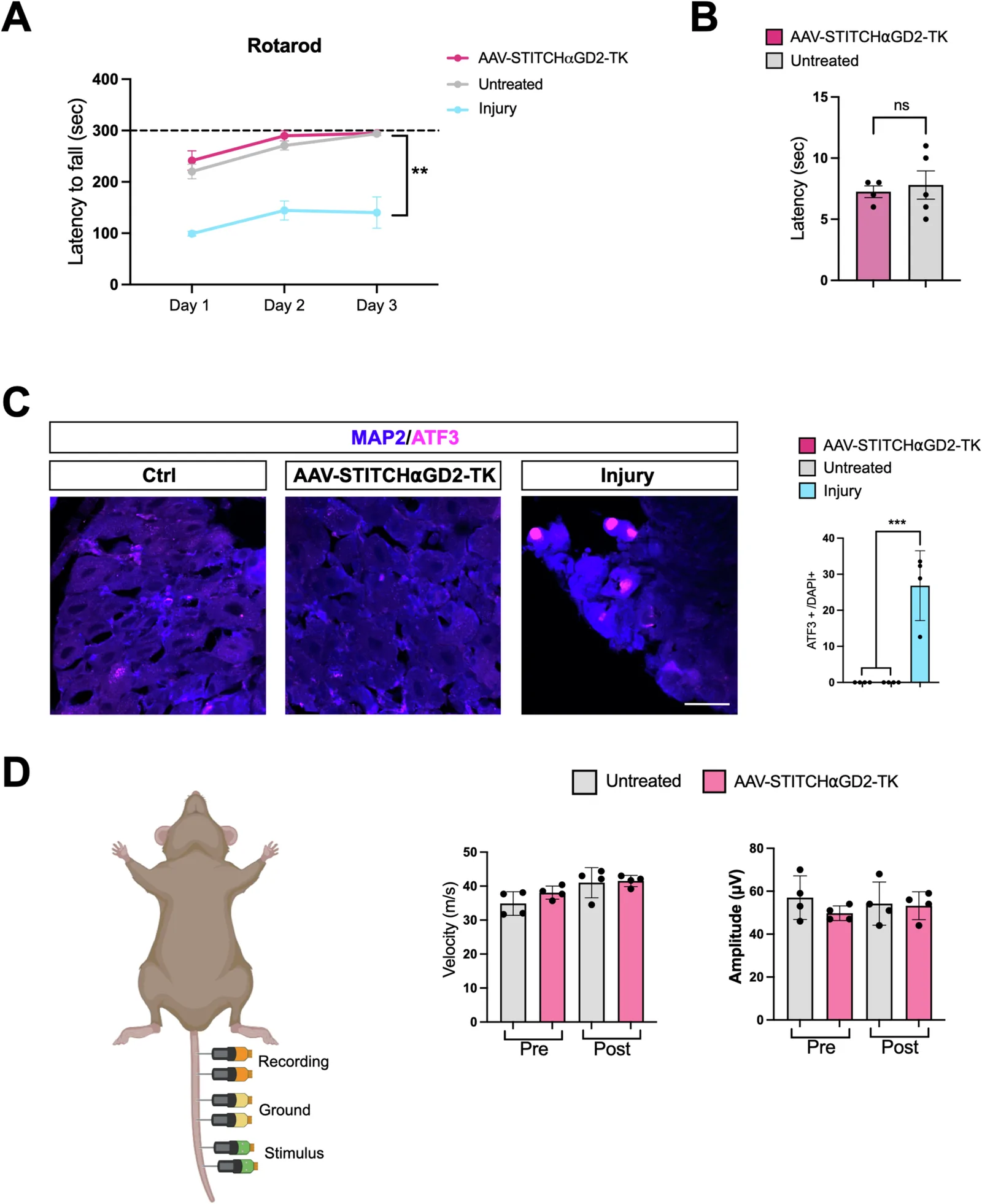
Toxicity assessment in dorsal root ganglia and peripheral nerves following AAV-STITCHαGD2 injection. (**A**) Evaluation of the latency to fall from the Rotarod analysis comparing WT untreated mice (gray line), sciatic nerve injured mice (Injury, light blue line) and AAV-STITCHαGD2 carrying HSV-TK (Purple line). *n* = 4 mice per group. Error bars represent SD. **P = 0.0083 AAV-STITCHαGD2-TK vs Injury; **P = 0.0069 Untreated vs Injury. Two-way ANOVA with Sidak’s multiple comparisons correction. (**B**) Evaluation of tactile sensitivity through hot plate test at 52 °C comparing WT untreated mice and treated with AAV-STITCHαGD2 carrying HSV-TK. Data are presented as mean ± SD, with individual data points shown (*n* = 4 mice per group). ns *P* > 0.05. *T* Test. (**C**) Left: immunofluorescence using anti-MAP2 and anti-ATF3 antibodies on DRGs derived from WT untreated mice (ctrl), injury (injured sciatic-nerve) and AAV-STITCHαGD2 carrying HSV-TK. Scale bar: 50 µm. Right: bar graph showing the quantification of ATF3+ cells over total cells (DAPI + ) in the three groups of DRGs. Data are presented as mean ± SD, with individual data points shown (*n* = 4 mice per group). ***P = 0.0001 AAV-STITCHαGD2-TK vs Injury; ***P = 0.0001 Untreated vs Injury. One-way ANOVA with Tukey’s multiple comparisons correction. (**D**) Left, schematic representation of tail sensory nerve conduction recordings. Sensory nerve action potentials (SNAPs) were recorded using stimulating and recording electrodes positioned along the tail nerve. Right, quantification of sensory nerve conduction velocity (m/s) and SNAP amplitude (µV) before (Pre) and after (Post) treatment in untreated and AAV-STITCHαGD2-TK + GCV treated mice. Data are presented as mean ± SD, with individual data points shown (*n* = 4 mice per group). Two-way repeated-measures ANOVA with Sidak’s multiple comparisons.

For ethical reasons, control mice were sacrificed once their tumors reached a diameter of approximately 1 cm, and at the same time point part of the treated cohort was also sacrificed to allow direct comparison of tumor burden. Histological inspection and volumetric measurements of the excised masses confirmed a marked reduction in tumor size in the AAV-STITCHαGD2-TK group relative to controls (Fig. 4D). Then, given DRG transduction, we performed behavioral, morphometric, and functional analyses, including electrophysiological assessment of tail nerve sensory responses, to evaluate potential DRG toxicity following viral treatment. C57BL/6 wild-type mice were injected with AAV-STITCHαGD2 carrying the TK gene and subsequently treated with GCV for 1 week, as done in NB-bearing mice. Animals subjected to sciatic nerve injury served as positive controls. Motor coordination was assessed using the accelerating rotarod task, and nociceptive sensitivity was evaluated with the hot plate test, two complementary measures reflecting DRG neuronal function (Fig. EV8A,B) (Hao et al, 2023). Remarkably, AAV-treated mice performed comparably to untreated controls, indicating no detectable impairment of DRG neuronal function (Fig. EV8A,B). Consistently, expression of ATF3, a stress-induced marker of DRG injury, remained undetectable after treatment, confirming the maintenance of a healthy, homeostatic state within DRGs (Fig. EV8C). In contrast, mice subjected to sciatic nerve injury exhibited clear behavioral deficits and robust ATF3 induction, validating the sensitivity and reliability of our experimental paradigm (Fig. EV8A–C). Finally, to assess potential peripheral sensory neuropathy, sensory nerve action potentials (SNAPs) were recorded from tail nerves before and after treatment. Measurements of sensory nerve conduction velocity and SNAP amplitude revealed no detectable functional impairment in AAV-STITCHαGD2-TK-treated mice compared to untreated controls (Fig. EV8D). Moreover, systemic analyses revealed no alterations in blood cell counts or liver enzyme levels at either short (3 days) or long (60 days) time points after viral treatment, further supporting the overall safety of this approach (Table EV1).

**Figure 5:**
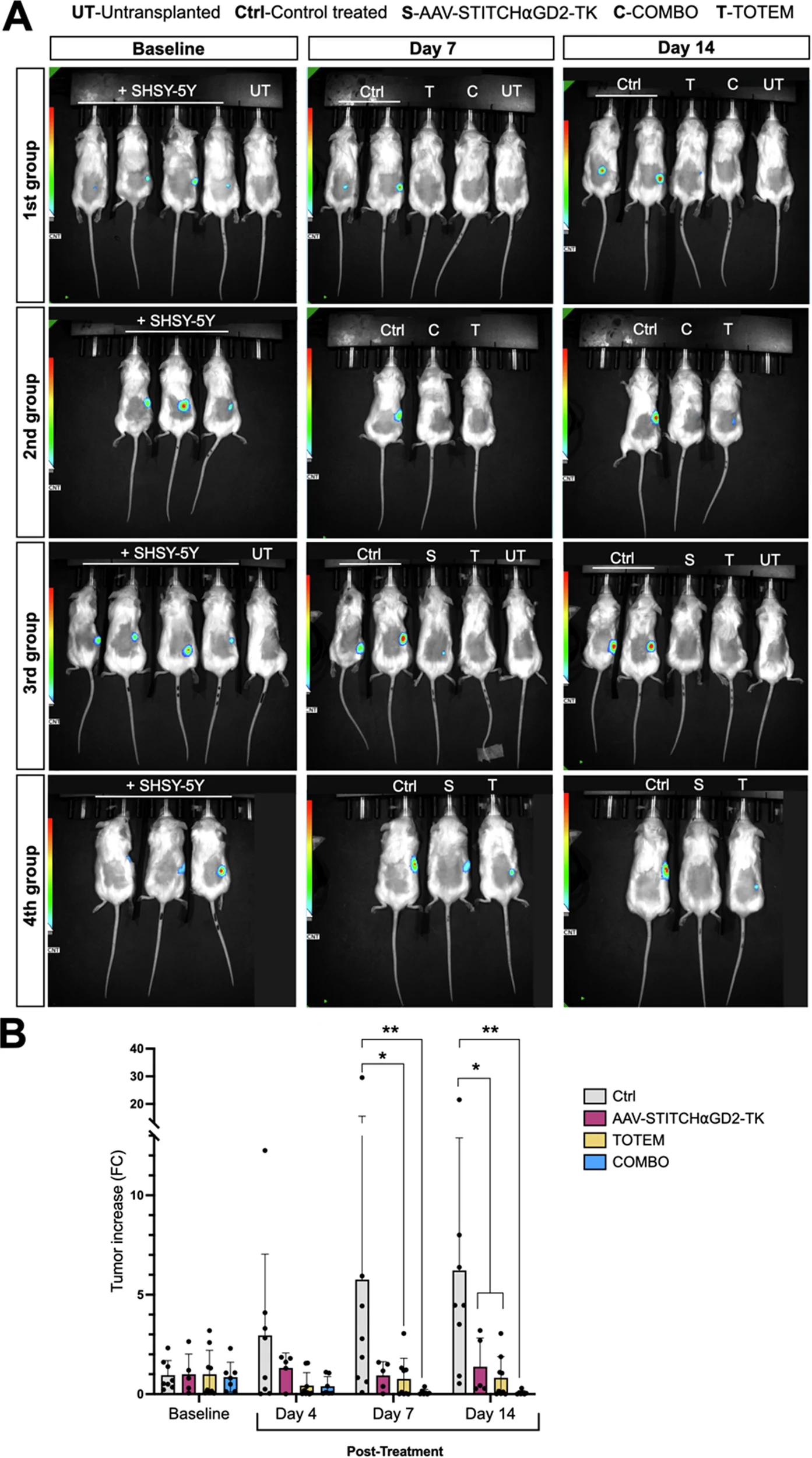
The combination of AAV-STITCHαGD2 and TOTEM exerts an additive antitumor effect in subcutaneous xenograft mouse model. (**A**) Representative bioluminescence images acquired with the IVIS Lumina system from three independent groups of mice: uninoculated (−) or inoculated subcutaneously with 2 × 10⁵ SH-SY5Y cells to generate xenograft tumors. Images were taken at three time points: baseline (pre-treatment), day 7, and day 14. For each condition, xenografted mice were divided into four groups: untreated controls (Ctrl), treated with Topotecan and Temozolomide (TOTEM group, T), treated with AAV-STITCHαGD2 + GCV (group S) or treated with TOTEM in combination with AAV-STITCHαGD2 + GCV (combination group, C). (**B**) Bar plot showing tumor growth quantified by bioluminescence imaging, measured as the signal within a region of interest (ROI) drawn over the tumor, at baseline and 4, 7, and 14 days post-treatment in mice untreated (Ctrl, *n* = 8 mice) or treated with AAV-STITCHαGD2 + GCV (*n* = 5 mice), TOTEM (*n* = 9 mice), or with TOTEM in combination with AAV-STITCHαGD2 + GCV (Combo, *n* = 7 mice). Data were normalized to baseline and are presented as mean ± SD, with individual data points shown. Day 7: *P = 0.0169 Ctrl vs TOTEM; **P = 0.0094 Ctrl vs COMBO. Day 14: *P = 0.0112 Ctrl vs TOTEM; *P = 0.0140 Ctrl vs STITCH; **P = 0.0040 Ctrl vs COMBO. Two-way ANOVA with Sidak’s multiple comparisons correction. Source data are available online for this figure.

To determine the real therapeutic applicability of this approach and to assess its potential compatibility with current clinical treatments, we carried out a direct comparison with the standard-of-care chemotherapy regimen combining TOpotecan and TEMozolomide (TOTEM), which is routinely used for relapsed NB in patients (Brignole et al, 2023; Rubie et al, 2010). Thus, NSG mice with established subcutaneous xenografts with Luc2-SH-SY5Y cells were randomly assigned to three experimental groups to receive TOTEM, TOTEM + AAV-STITCHαGD2-TK or saline (control) (Fig. 5A). TOTEM alone significantly slowed the tumor growth in the following 14 days with a similar magnitude as previously described for AAV-STITCHαGD2-TK (Fig. 5A,B). However, the combined treatment showed the strongest antitumor effect, reducing tumor burden to almost undetectable levels by bioluminescence for the following 14 days, with a greater reduction compared to control than either monotherapy alone (tumor reduction vs control after 14 days: AAV-STITCHαGD2-TK = 62% ±  22%; TOTEM = 73% ± 5%; AAV-STITCHαGD2-TK + TOTEM = 97% ± 1.6%) (Fig. 5A,B). These results demonstrate the additive effects of the two approaches, which act through complementary mechanisms on tumor cells, thereby providing a promising best-in-class therapeutic strategy.

### AAV-STITCHαGD2-TK dampens the detrimental effects of metastatic NB and inhibits patient-derived xenograft growth

**Figure 6:**
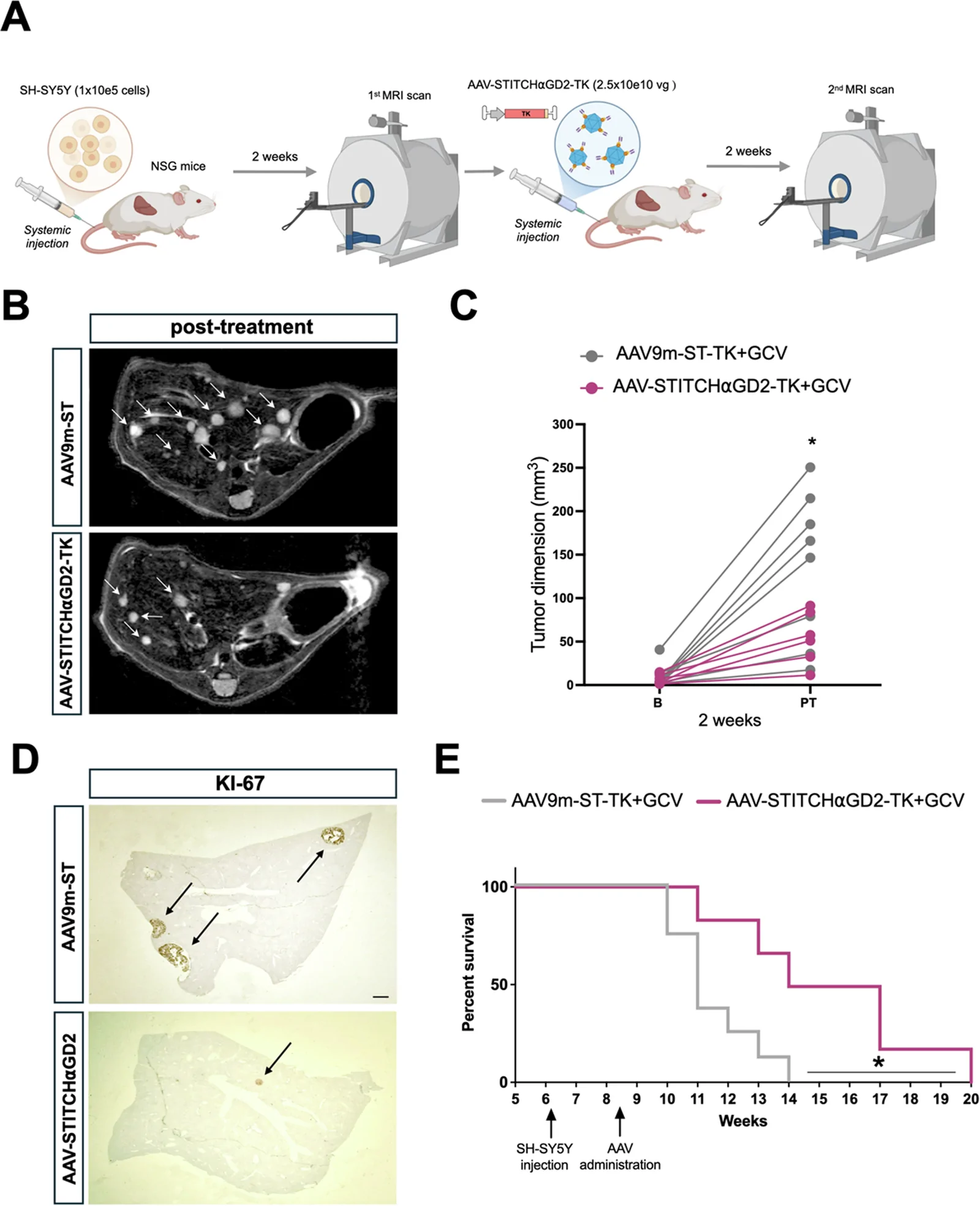
TK-expressing AAV-STITCHαGD2 inhibits neuroblastoma hepatic metastasis growth and significantly extends animal survival. (**A**) Scheme of the experimental protocol. In brief, 2 × 10^5^ SH-SY5Y cells were systemically injected into adult NGS mice (pseudometastatic model). Two weeks later, metastasis volume was determined by MRI and AAV-STITCHαGD2 or AAV9m-ST (negative control) carrying HSV-TK transgene were inoculated in the tail vein using 5 × 10^10^ vg/mouse. The following week, mice were daily injected with GCV and after 10 days a second MRI scan was performed. (**B**) Representative MRI images of liver metastasis after treatment comparing control (AAV9m-ST + GCV) and treated (AAV-STITCHαGD2 + GCV) livers (arrows indicate metastases). (**C**) Plot representing the quantification of the volume of liver metastasis at the first (baseline) and at the second (post-treatment) scan in mice treated with AAV-STITCHαGD2 + GCV (*n* = 6 mice, purple) or AAV9m-ST + GCV (*n* = 8 mice, gray). PT: *P = 0.039 AAV9m-ST + GCV vs AAV-STITCHαGD2 + GCV. Two-way ANOVA with Sidak’s multiple comparisons correction. (**D**) Representative IHC staining for Ki67 in liver metastases from control mice (AAV9m-ST) and mice treated with AAV-STITCHαGD2, showing smaller and fewer metastatic lesions in the treated group. Scale bar: 500 µm. (**E**) Survival plot of mice treated with AAV-STITCHαGD2 (purple) or AAV9m-ST (gray) expressing HSV-TK + GCV. Statistical significance was determined using the Mantel–Cox (log-rank) test (*P = 0.041). Source data are available online for this figure.

Next, we ascertained the effects of the TK-expressing AAV-STITCHαGD2 in the pseudometastatic xenograft NB mouse model. Based on our previous validation that small hepatic metastatic lesions become detectable by MRI 2 weeks after intravascular administration of SH-SY5Y NB tumor cells, this time point was selected to initiate treatment. Animals were then imaged to identify those with comparable numbers of detectable liver metastases and randomized into two groups to receive either AAV-STITCHαGD2-TK or the control (AAV9m-ST-TK) viral treatment (2.5 × 10e10 vg), followed by GCV administration (Fig. 6A). Two weeks after viral infusion, a second MRI scan revealed a marked reduction in both the number and size of NB hepatic metastases in animals treated with AAV-STITCHαGD2-TK compared to controls (53.78 ± 6.34% of mean tumor growth inhibition) (Fig. 6B,C). The marked inhibition of metastatic growth was further confirmed by immunohistochemical visualization of Ki67+ proliferating cells in AAV-STITCHαGD2-TK treated liver tissues (Fig. 6D). Additionally, AAV-STITCHαGD2-TK-treated mice showed a significant extension in survival compared to animals treated with the control vector (Fig. 6E).

**Figure EV9:**
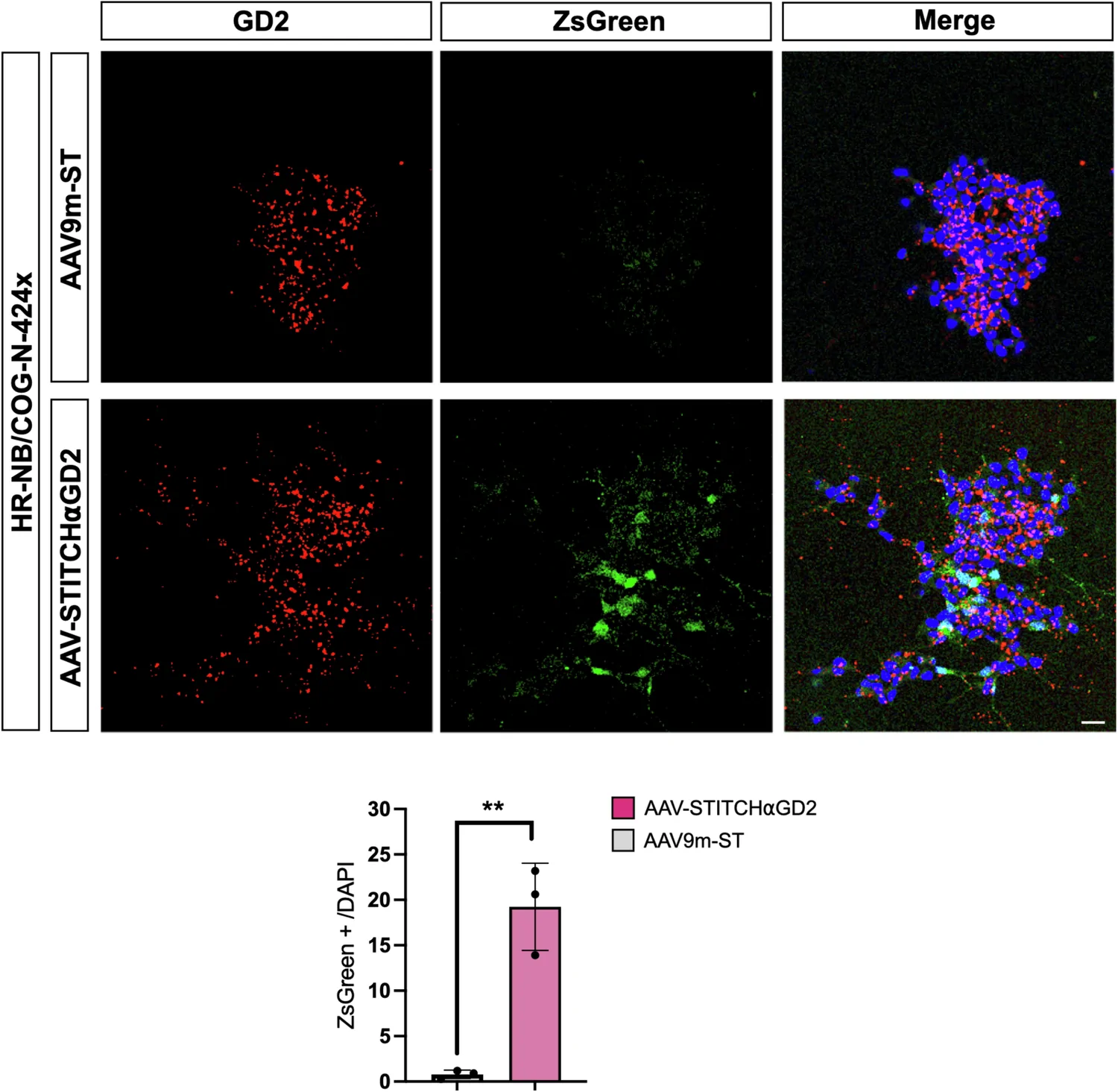
Evaluation of AAV-STITCH⍺GD2 transduction efficiency in patient-derived cancer cells. Representative immunostaining using anti-ZsGreen and anti-GD2 antibodies on patient-derived cancer cells transduced with AAV-STITCH⍺GD2 or AAV9m-ST as control to vehicle ZsGreen transgene and bar graph showing the percentage of ZsGreen+ cells over total (DAPI+) after transduction with AAV-STITCH⍺GD2 with respect the control AAV9m-ST. Scale bar: 100 µm. Data are presented as mean ± SD, with individual data points shown (*n* = 3 independent biological replicates). ***P = 0.0009, *t* Test.

**Figure 7:**
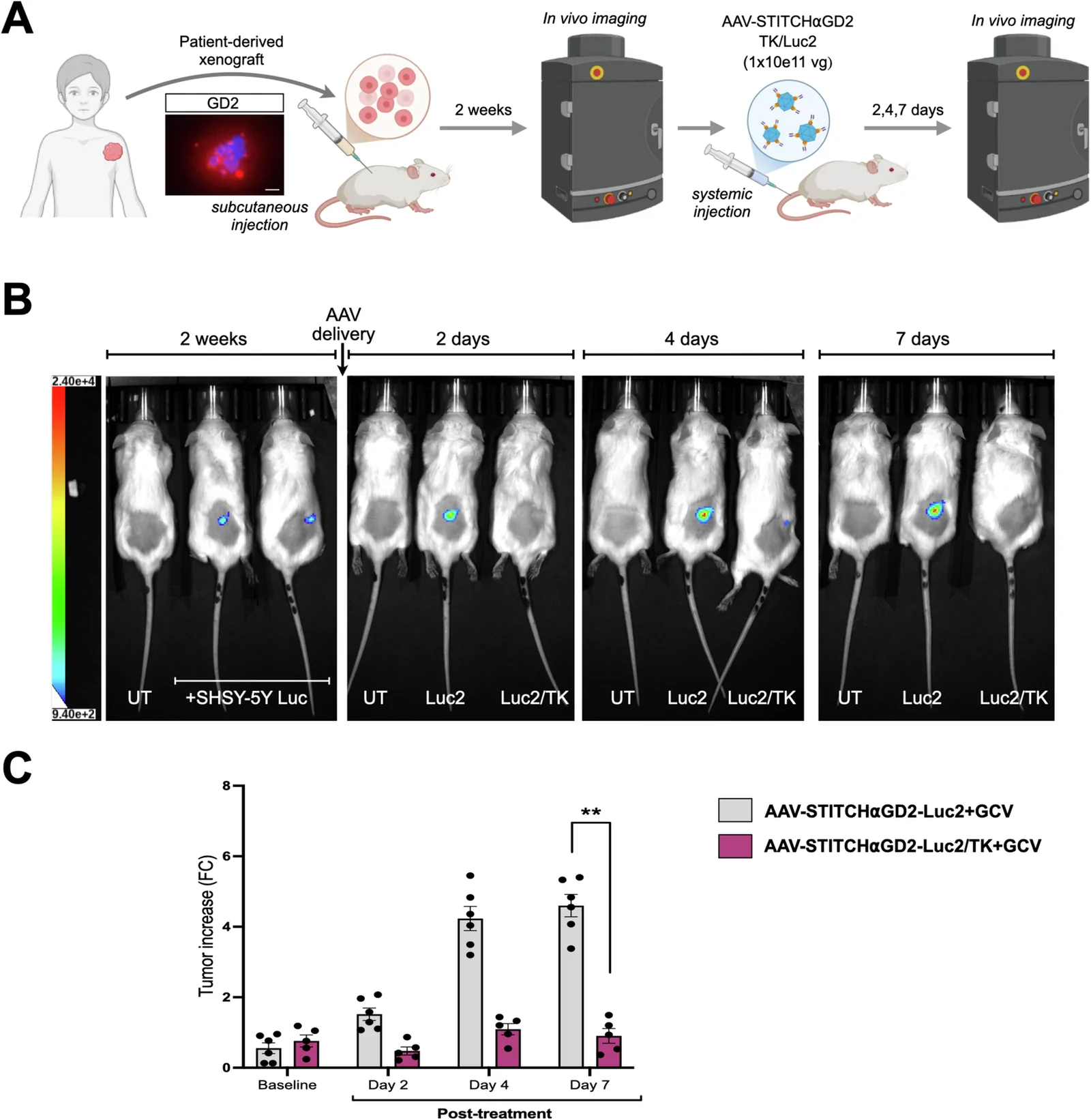
TK-expressing AAV-STITCHαGD2 inhibits the growth of subcutaneous tumor in PDX mouse model. (**A**) Scheme of the experimental protocol. Adult NGS mice were subcutaneously injected with GD2+ patient-derived cancer cells (Scale bar: 25 µm) (PDX model). Two weeks later, mice were injected with AAV-STITCHαGD2 or AAV9m-ST (negative control), each administered as two separate vectors carrying the HSV-TK and luciferase transgenes, via tail vein injection at a dose of 5 × 10¹⁰ vg/mouse for each viral vector. Five days later, baseline tumor burden was assessed by IVIS imaging. Starting the following week, mice received daily injections of GCV, and tumor growth was monitored by IVIS imaging over 2, 4 and 7 days. (**B**) Representative bioluminescence images acquired at different time points (baseline, 2, 4 and 7 days) using the IVIS Lumina system from PDX mice treated with luciferase alone + GCV (AAV-STITCHαGD2-Luc2+GCV; Luc2 group) or luciferase + HSV-TK + GCV (AAV-STITCHαGD2-Luc2/TK + GCV; Luc2/Tk group), shown at different experimental time points. (**C**) Bar plot showing tumor growth quantified by bioluminescence imaging, measured as the signal within a region of interest (ROI) drawn over the tumor, at baseline and 2, 4, and 7 days post-treatment in mice treated with AAV-STITCHαGD2-Luc2+GCV (*n* = 6 mice, gray) or AAV-STITCHαGD2-Luc2/TK + GCV (*n* = 5 mice, purple). Data were normalized to baseline and are presented as mean ± SD, with individual data points shown. Day 7: **P = 0.0016 AAV-STITCHαGD2-Luc2+GCV vs AAV-STITCHαGD2-Luc2/TK + GCV. Two-way ANOVA with Sidak’s multiple comparisons correction. Source data are available online for this figure.

Finally, we tested the anti-tumoral activity of AAV-STITCHαGD2-TK in a patient-derived xenograft (PDX) mouse model. MYCN-amplified tumor explants from one patient with high-risk NB (HR-NB/COG-N-424x) were obtained from the Childhood Cancer Repository (Texas, USA) and showed to express GD2 and be permissive to AAV-STITCHαGD2 transduction in vitro (Fig. EV9). NSG mice were grafted subcutaneously and 2 weeks later, they received intravascular injections of AAV-STITCHαGD2-TK vectors expressing either Luc2 and TK, or Luc2 alone as a control (1 × 10e11 vg for each vector), allowing longitudinal intravital imaging of tumor growth following treatment (Fig. 7A). Longitudinal imaging at days 4 and 7 after treatment revealed a marked suppression of tumor growth in the AAV-STITCHαGD2-TK group compared to controls (Fig. 7B,C). In control mice, Luc2 signal progressively increased from day 0 to day 4, then plateaued by day 7, likely reflecting continued dilution of viral genomes in the proliferating tumor cells over time. In contrast, the Luc2 signal in the TK-treated group did not increase, indicating that luciferase-expressing tumor cells were efficiently eliminated following activation of the suicide gene (Fig. 7B,C). These findings confirm the potent anti-tumoral effects of AAV-STITCHαGD2-TK in suppressing malignant growth of primary human NB tissue, highlighting its strong translational potential.

## Discussion

Tailored capsid modifications have driven the successful development of engineered AAV capsids with desirable new properties. Nevertheless, the broad, multi-tissue tropism of native AAV capsids remains a significant barrier to the development of safer clinical applications. Previous strategies to modify the capsid surface have included the conjugation of molecules via peptide fusions with the viral VP2 or VP3 proteins (Münch et al, 2013, 2015). However, it is yet unpredictable a priori whether any incorporated peptide can compromise capsid assembly or viral yield. More recently, site-specific incorporation of unnatural amino acids into the AAV capsid has enabled the covalent attachment of molecules through click chemistry (Kelemen et al, 2018; Puzzo et al, 2023). While promising, this method is limited by reduced AAV production titers and a labor-intensive, multi-step workflow. However, a direct comparison with these strategies would be of interest to further define their respective advantages and limitations.

Herein, we have developed a novel conjugation system that leverages the high affinity of the SpyTag/SpyCatcher interaction to enable efficient and specific covalent coupling of polypeptides to the surface of the AAV capsid. Similar SpyTag/SpyCatcher-based strategies for ligand conjugation onto AAV particles have recently been reported (Chen et al, 2025; Armbruster et al, 2026). However, these approaches rely on post-production conjugation steps and/or the incorporation of the SpyCatcher into the capsid, which can limit vector yield and increase experimental complexity. In addition, the use of a conventional AAV9 capsid preserves its natural peripheral tropism, thereby significantly compromising targeting specificity and increasing the risk of off-target transduction–related adverse effects. In contrast, our co-expression strategy enables the *in cellulo* formation of the SpyTag–SpyCatcher bond during viral production, generating functional antibody–AAV conjugates in a single step without the need for additional purification. This streamlined workflow not only simplifies and accelerates vector generation but also enhances conjugation efficiency, yield, and reproducibility, providing a more versatile and scalable platform for targeted AAV engineering.

By incorporating the SpyTag into a permissive region of the infectivity-deficient AAV9-W503A vector, we created a capsid whose tropism can be reprogrammed by displaying different targeting molecules on its surface. Thus, this system serves as a flexible and modular platform that can be readily exploited by fusing any tropism-guiding molecule to the SpyCatcher. Importantly, we further corroborate that the W503A mutation completely abolishes the infectivity of AAV9, generating a capsid devoid of residual off-target transduction. We also validated the conjugation with two distinct scFv molecules, obtaining consistent results in terms of antigen specificity and transduction efficiency. Therefore, we surmise that this approach has broad applicability for expanding the use of AAV vectors in cell type-specific applications.

Surprisingly, we found that the high coupling of the SC-αGD2 onto the capsid is reducing the viral infectivity of GD2 + NB cell lines, an effect similarly observed with the HER2-targeting construct. In vitro analyses suggest that this effect was not due to impaired viral binding to the cell surface, but rather to defects occurring at subsequent steps of the viral transduction process. This impairment aligns with previous observations showing that an excessive coupling of FITC molecules onto the AAV2 capsid reduced its infectivity in HEK293 cells (Katrekar et al, 2018). It is likely that excessive molecule display masks the recognition of AAV co-receptors on the membrane surface or along the intracellular trafficking route. Consistently, we have shown that interaction with AAV-R is essential for efficient transduction, as its knockdown markedly impairs viral infection. More studies are warranted to define which step of the AAV transduction cycle is affected by excessive scFv loading on the capsid surface. In line with this, the 1:3 ratio used in our protocol yielded the best overall performance with both scFv constructs tested. Although this ratio cannot be considered universally optimal, it provides a robust starting point for further optimization across different targeting modalities. A deeper understanding of the mechanisms governing AAV transduction will be essential to rationally refine capsid engineering strategies and improve their efficiency and specificity.

We prioritize the coupling of αGD2 moieties to develop a valuable anti-tumor agent targeting NB. GD2 is a highly and consistently expressed antigen in this tumor type, a feature that has already been successfully exploited in antibody-based and CAR T cell therapies (Pule et al, 2008; Straathof et al, 2020; Del Bufalo et al, 2023). However, these immunotherapies face limitations due to the immunosuppressive tumor microenvironment and potential off-target effects (Kankeu Fonkoua et al, 2022). Importantly, the tropism and therapeutic efficacy of our viral approach are not affected by the tumor’s immune status. Although GD2 is also expressed in DRG neurons, these cells exhibited only limited transduction, and no signs of peripheral toxicity were detected in treated mice at the histological, functional or behavioral levels. The STITCHαGD2-TK system has a significant lower tropism for these neurons respect to the native AAV9 capsid increasing its safety profile. Moreover, the AAV-STITCHαGD2-TK system poses an even reduced risk to these cells, as the TK/GCV suicide gene system is selectively active in proliferating cells. We showed that the STITCHαGD2-TK system. Nevertheless, this aspect should be carefully considered, and future efforts should aim to implement additional detargeting strategies to further minimize potential off-target effects in peripheral tissues. Furthermore, the vector design offers the flexibility to incorporate additional regulatory elements, such as cell cycle–specific promoters or cell type-specific microRNA target sequences, to further enhance the precision of TK expression. The strong anti-tumoral effects of TK/GCV treatment relies also by its significant bystander effect since the phosphorylated GCV is passively transferred to surrounding TK-non-expressing tumor cells inducing their cell death (Oishi et al, 2022). Moreover, TK/GCV-mediated cell death might trigger inflammation and T cell recruitment harnessing the immune response against the tumor (Umegaki et al, 2023). We demonstrated that AAV-STITCHαGD2-TK achieves tumor inhibition comparable to standard-of-care chemotherapy. Moreover, the combination of both treatments produced superior antitumor efficacy compared with either treatment alone in a 14-day study using subcutaneous xenografted mice. Although not directly tested in this study, these therapeutic approaches could also be explored in a sequential rather than concomitant treatment setting. These encouraging results warrant further investigation in additional preclinical models to accelerate validation for clinical application.

A limitation of AAV-based therapies for proliferating tumor cells is the progressive dilution of the episomal viral vector, resulting in a gradual loss of therapeutic efficacy. This inherent characteristic represents an obstacle to the development of AAV-based gene therapy clinical protocol as a stand-alone treatment for highly proliferative malignancies, where sustained and long-term transgene expression is often required to achieve durable tumor control. However, we demonstrated that a single treatment can provide long-lasting survival benefits in an aggressive pseudometastatic xenograft mouse model, suggesting a significant impact on tumor evolution. Importantly, AAVs can efficiently infect non-dividing cells and may therefore target quiescent cancer stem cells, which are thought to promote resistance to standard therapies and tumor recurrence in NB (Veschi et al, 2019; Walton et al, 2004). Thus, this viral therapy may represent a suitable adjuvant to conventional anti-tumor treatments, given its ease of implementation and independent mechanism of action. AAV-based therapies can typically be administered only once, and only to seronegative individuals. However, since NB primarily affects children, a population with a lower prevalence of pre-existing AAV immunity, it is estimated that ~80% of patients would be eligible (Calcedo et al, 2011). Nevertheless, treated individuals are expected to develop neutralizing antibodies against the viral capsid, thereby preventing repeated administration and representing a major limitation of current AAV-based approaches. In addition, immunogenic responses may also arise against the SpyTag/SpyCatcher components, potentially further restricting redosing. Despite these challenges, recent advances in the development of engineered AAV capsids with reduced immunogenicity, together with emerging clinical strategies to overcome pre-existing immunity, are expected to enable repeated vector administration in the near future (Chan et al, 2021; Mietzsch et al, 2025; Meliani et al, 2018). These include recently developed immune-evasion approaches such as plasmapheresis (Potter et al, 2024) or the use of engineered IgM- and IgG-cleaving enzymes (Leborgne et al, 2020; Smith et al, 2024). Moreover, the availability of multiple orthogonal SpyTag/SpyCatcher variants provides an additional opportunity to circumvent immune recognition, allowing sequential dosing strategies without cross-reactivity (Veggiani et al, 2016).

In summary, we have developed AAV-STITCH, a rapid and versatile conjugation system that enables the targeted coupling of polypeptides to the surface of an infectivity-deficient AAV9 capsid, facilitating cell-type-specific viral transduction. This platform addresses longstanding challenges in the field by improving AAV selectivity and gene transduction efficiency, thereby expanding the potential of AAV-based therapies in clinical applications. As a compelling proof-of-concept, we engineered the AAV-STITCHαGD2 system, which functions as a potent anti-tumor agent capable of inhibiting the growth of NB xenografts derived from both established tumor cell lines and primary tumor tissues. This study introduces and validates a new viral anti-tumor strategy with unique, complementary advantages over existing therapeutic options. Consequently, this approach may represent a promising adjuvant therapeutic strategy with the potential to broaden and strengthen the therapeutic options available for patients with NB.

## Methods

**Table:** Reagents and tools table

Reagent/resource	Reference or source	Identifier or catalog number
**Experimental models**		
C57BL/6NCrl	Charles River Laboratories	https://www.criver.com/products-services/find-model/c57bl6-mouse?region=27
HEK293T cells	ATCC	Cat#CRL-3216
SH-SY5Y	ATCC	Cat#CRL-2266
NSG mice (NOD.Cg-*Prkdc*^*scid*^ *Il2rg*^*tm1WjI*^/SzJ)	Charles River Laboratories	https://www.criver.com/products-services/find-model/jax-nsg-mice?region=27
PDX samples	Childhood Cancer Repository	N/A
HTLA-230 (STR certified)	Dondero et al, 2015	N/A
SK-N-SH (STR certified)	Dondero et al, 2015	N/A
GI-ME-N (STR certified)	Paret et al, 2025	N/A
LAN-5 (STR certified)	Paret et al, 2025	N/A
IMR-32 (STR certified)	Brignole et al, 2021	N/A
**Recombinant DNA**		
pHelper	Agilent	Cat#240071
14G2a scFv	Gargett et al, 2016, Synthesized, Azenta Genewiz	N/A
SpyCatcher peptide	Synthesized, Azenta Genewiz	N/A
scFv αHER2	Addgene	Cat#183249
scFv-αGFP	Addgene	Cat#199429
pcDN/A3.1 plasmid	Addgene	Cat#2023
scAAV-CMV-GFP	Addgene	Cat#32396
HSV-TK A168H	Oishi et al (2022), Synthesized Azenta Genewiz	N/A
Luciferase CDS	Addgene	Cat#17477
ZsGreen CDS	Addgene	Cat#124302
Lv-Ef1a-IRES-Puro vector	Addgene	Cat#85132
TetO-Ngn2-P2A-EGFP-T2A-Puro vector	Rubio et al (2016)	N/A
GDS2-P2A-GD3S CDS	Synthetized, Azenta Genewiz	N/A
AAV9-W503A capsid	Giannelli et al (2024)	N/A
AAV9-W503A-ST capsid	This paper	N/A
14G2a scFv-SpyCatcher	This paper	N/A
VSV-G Envelope plasmid	Addgene	Cat#12259
pSUPER plasmid	MERK	Cat#VEC-PBS-0002
**Antibodies**		
Living Colors® ZsGreen polyclonal antibody	Takara Bio	Cat#632474
anti-V5 antibody	Thermo Scientific	Cat #R960-25
anti-AAV VP1/VP2/VP3 mouse monoclonal antibody	Progen	Cat# 690058
HRP-conjugated secondary antibody anti-mouse	Dako	Cat#P0447
Anti-Ganglioside GD2 antibody [14.G2a]	Abcam	Cat# ab68456
PE Mouse Anti-Human Disialoganglioside GD2	BD Biosciences	Cat#562100
Alexa Fluor 594	Thermo Fisher Scientific	Cat#A-11032
Anti-Ki67 antibody	Abcam	Cat# ab16667
Alexa Fluor™ 488 Goat Anti-Rabbit IgG	Thermo Fisher Scientific	Cat#A-11008
MAP2 monoclonal antibody	Thermo Fisher Scientific	Cat#13-1500
ATF3 monoclonal antibody	Abcam	Cat#ab207434
**Oligonucleotides and other sequence-based reagents**		
**SpyTag-OEP-R** cttgtaggcgtccaccatcacgatgtgggcgccgctcaggccgctgccgctgctcaggccaccgttaatagtTCtAgagagatagtacaa	Zakeri et al (2012)	N/A
**SpyTag-OEP-F** tcgtgatggtggacgcctacaagccgacgaagggcctgagcggcggcagcggcctgagcggctctggacagaatcaacaaacgctaaaat	Zakeri et al (2012)	N/A
**ZsG _RTprimer_F** TCCAAGCACGGCCTGACCA AGGAGA	This paper	N/A
**ZsG _RTprimer_R** GGCAAGGCGGAGCCGGAGGCGATG	This paper	N/A
**AAVR shRN/A** GCTGAGATGGTAGCATAGAAA	This paper	N/A
**Scramble shRN/A** CCTAAGGTTAAGTCGCCCTCG	This paper	N/A
**AAVR _RTprimer_F** TGGCTCCCTCCTACAGTTATG	This paper	N/A
**AAVR _RTprimer_R** GGCAGGGTTATCTGGACACT	This paper	N/A
**18srRN/A _RTprimer_F** GTAACCCGTTGAACCCCATT	This paper	N/A
**18srRN/A _RTprimer_R** CCATCCAATCGGTAGTAGCG	This paper	N/A
**Chemicals, enzymes and other reagents**		
NheI-HF enzyme	NEB	Cat# R3131L
AgeI-HF enzyme	NEB	Cat# R3552L
Luria Broth (LB)	MERCK	Cat# L3022-1kg
Ampicillin	MERCK	Cat# A9518
Polyethyleneglycol 8000	MERCK	Cat# P2139
OptiPrep™ (Iodixanol commercial solution)	MERCK	Cat# D1556
Polyethylenimine 25 K (PEI-25K)	Polyscience	Cat# 23966
BamHI-HF	NEB	Cat#R3136T
NotI-HF	NEB	Cat#R3189L
EcoRI-HF	NEB	Cat#R3101T
XbaI	NEB	Cat# R0145S
Dulbecco Modified Eagle Medium high glucose	ThermoFisher	Cat#D5796
fetal bovine serum	MERK	Cat#F7524
MEM Non-Essential Amino Acids Solution	ThermoFisher	Cat#11140050
Sodium Pyruvate	Gibco	Cat#11360070
L-Glutamine	MERK	Cat#G5792
Penicillin/streptomycin	MERK	Cat#P4333
Trypsin 0.25%	MERK	Cat#T4049
Poly(ethilene Glycol) 8000 (PEG)	MERK	Cat#89510
Trizma base	MERK	Cat# T1503
Sodium chloride	MERK	Cat# S7653
Magnesium chloride	MERK	Cat#M8266
Salt Active Nuclease (SAN)	Arcticzymes	Cat#70900-202
PBS (Phosphate Buffer Saline)	MERK	Cat# P4474
Pro293TMa Serum-free Medium – Chemically Defined for 293 Adherent Cells	Lonza	Cat#BEBP12-764Q
Amicon® Ultra, MWCO 10 kDa	MERK	Cat#UFC9010
Sodium phosphate dibasic, anhydrous	MERK	Cat#S9763
Ni Sepharose™ 6 Fast Flow	MERK	Cat#GE17-5318-06
Imidazole	MERK	Cat#I202
4× Laemmli Sample Buffer	Bio-Rad	Cat#1610747
SDS Running Buffer	Bio-Rad	Cat#1610732
Amersham™ ECL™ Rainbow™ Marker – Full range	MERK	Cat#RPN800E
Ponceau S Staining Solution	Bio-Rad	Cat#1610430
Paraformaldehyde	MERK	Cat# 158127-500 G
SDS (sodium dodecyl sulfate)	MERK	Cat#L3771
β-mercaptoethanol	MERK	Cat#M3148
DTT(Dithiothreitol)	MERK	Cat#D9163
30% Acrylamide/Bis Solution	Bio-Rad	Cat#1610158
Ammonium Persulfate (APS)	Bio-Rad	Cat#1610700
N,N,N′,N′-Tetramethylethylenediamine (TEMED)	MERK	Cat#T9281
Glycine	MERK	Cat#G8898
Trans-Blot® Turbo™ 5× Transfer Buffer, 1 L	Bio-Rad	Cat#10026938
TWEEN® 20	MERK	Cat#P1379
SuperBlot™ ECL Western Blotting Substrate Kit – Standard	Hello Bio	Cat#HB7090
Calcium chloride	MERK	Cat#C5670
RPMI 1640 Medium	Gibco	Cat#11875093
Puromycin Dihydrochloride	MERK	Cat#P8833
DAPI (4′,6-diamidino-2-phenylindole), dilactate	Thermo Fisher Scientific	Cat#D3571
Donkey serum	MERK	Cat#D9663
Dako Fluorescence Mounting Medium	Dako	Cat#S3023
Target Retrieval Solution, Citrate pH 6.0 (10×)	Dako	Cat#S2369
Hematoxylin solution	MERK	Cat# MHS16
Eosin Y alcoholic solution	MERK	Cat# HT110116
3,3′-Diaminobenzidine tetrahydrochloride (DAB)	MERK	Cat# D5637
Neurobasal™ Medium	Gibco	Cat# 21103049
B-27™ Supplement (50×)	Gibco	Cat# 17504044
D-(+)-Glucose	MERK	Cat# G7021
Tissue-Tek® O.C.T. Compound	Sakura Finetek	Cat# 4585
NP-40 AlterN/Ative	MERK	Cat# 492016
Ganciclovir	MERK	Cat#G2536
D-Luciferin, potassium salt	MERK	Cat#L9504
Lipofectamine™ LTX Reagent with PLUS™ Reagent	Thermo Fisher Scientific	Cat#A12621
NucleoZOL	MACHEREY-N/AGEL	Cat# 740404.200
Matrigel® Growth Factor Reduced (GFR)	Corning	Cat#354230
Sucrose	MERK	Cat#107687
Triton™ X-100	MERK	Cat#X100
TOpotecan + TEMozolomide (TOTEM)	Capasso et al, 2024, Giannina Gaslini Hospital	N/A
**Software**		
MIPAV (Medical Image Processing, Analysis, and Visualization)	National Institutes of Health (NIH), Center for Information Technology (CIT)	https://mipav.cit.nih.gov
AURA Image Analysis Software	Revvity	https://spectralinvivo.com/software/
Prism version 10.5.0	GraphPad Software	https://www.graphpad.com/
FlowJo version 10.10.0	BD Biosciences	https://www.flowjo.com/
**Other**		
Amicon Ultra15	MERK	Cat# UFC910024
AAVpro© Titration Kit Ver2	TaKaRa	Cat# 6233
Trans-Blot® Turbo™ Transfer System	Bio-Rad	N/A
Trans-Blot® Turbo™ Mini 0.2 µm Nitrocellulose Transfer Packs	Bio-Rad	Cat#1704158
Coomassie Brilliant Blue R-250 Staining Solution	Bio-Rad	Cat#1610436
Ultraclear tubes (13.2 mL, 14 mm X 89 mm)	Beckmann Coulter	Cat# 344059
CytoFLEX S flow cytometer	Beckmann Coulter	N/A
Protein Deglycosylation Mix II	NEB	Cat#P6044S
ChemiDoc imaging system	Bio-Rad	N/A
Corning® bottle-top vacuum filter system	Merk	Cat#CLS431155
Optima XE ultracentrifuge	Beckmann Coulter	N/A
Tumor Dissociation Kit	Miltenyi Biotec	Cat#130-095-929
Nikon Eclipse 600 fluorescence microscope	Nikon	N/A
Mouse and Rabbit Specific HRP (ABC) Detection IHC Kit	Abcam	Cat# ab93697
Porous membrane inserts, 6-well	Merck	Cat# PICMORG50
RS-G4 Resonant-Scanning Confocal Microscope	MAVIG Gmbh	N/A
QIAamp DNA Micro Kit	QIAGEN	Cat# 56304
LIVE/DEAD™ Viability/Cytotoxicity Kit, for mammalian cells	Thermo Fisher Scientific	Cat# L3224
LIVE/DEAD™ Fixable Near-IR Dead Cell Stain Kit	Thermo Fisher Scientific	Cat#L34975
IVIS Spectrum In Vivo Imaging System	Revvity	N/A
MRI 7tesla	N/A	N/A
IDEXX ProCyte analyzer	IDEXX Laboratories	N/A
RotaRod	Ugo Basile	Cat#47650

### Study design

The main objective of this study was to develop a new method for covalently coupling antibodies to the AAV capsid in order to establish an optimized therapeutic strategy for high-risk NB. This approach was based on the use of the SpyTag/SpyCatcher peptide system to achieve covalent fusion of the viral capsid with a single-chain variable fragment (scFv) directed against the GD2 antigen, one of the best-characterized NB antigen. We selected the AAV9 serotype because a well-characterized single point mutation can completely abolish its natural tropism, allowing its redirection toward tumor cells through the scFv. We termed this new technology AAV-STITCH.

Initially, we optimized the production strategy by comparing two different coupling protocols—post-production coupling and co-expression coupling—and determined through biochemical analyses that the co-expression approach ensured higher conjugation efficiency and purity. We then validated the transduction efficiency and specificity of AAV-STITCH using various cellular models, including engineered cell lines to force GD2 expression, and assessed transduction and targeting through immunofluorescence and flow cytometry analyses.

After these in vitro validations, we assessed the in vivo targeting specificity and efficiency of AAV-STITCHαGD2, which selectively transduced tumor cells. For all in vivo experiments, a minimum of three mice per group was used to ensure data reliability and reproducibility. During these analyses, we observed that dorsal root ganglia (DRG) neurons were also transduced, prompting us to evaluate the safety profile of our approach. Specifically, we confirmed the absence of peripheral nerve toxicity following injection of the suicide gene HSV-TK, which was used in the final therapeutic experiments.

Once both in vitro and in vivo validations were completed, we generated three independent mouse models of NB to characterize the therapeutic efficacy of AAV-STITCHαGD2 delivering HSV-TK. All experiments were designed to ensure robust statistical reproducibility, including the use of ≥5 mice per group, random allocation of animals to experimental groups, and blinded analysis by operators unaware of treatment conditions (e.g., during MRI imaging). Finally, we compared the effect of our therapeutic platform with the current standard-of-care treatment (Topotecan and Temozolomide), evaluating the potential for additive effects with chemotherapeutic agents currently used in NB treatment. We also demonstrated the versatility of the AAV-STITCH system by testing its applicability to another tumor-associated receptor (HER2). Ethical considerations were fundamental for this study. All animal experiments were reviewed and approved by the Animal Care and Use Committee (IACUC) of the San Raffaele Scientific Institute, in compliance with institutional and national ethical regulations.

### Plasmids

The SpyTag peptide sequence was inserted into the AAV9-A503W plasmid between amino acids 436 and 475 using overlap extension PCR (OEP), exploiting the NheI and AgeI restriction sites. The SpyCatcher peptide sequence, fused to the scFv-αGD2 (a kind gift from M. Brenner), to scFv αHER2 (Addgene #183249) or to scFv-αGFP (Addgene #199429), was synthesized by Azenta Genewiz and subcloned into pcDNA3.1 plasmid (Addgene #2023). scAAV-CMV vectors carrying the ZsGreen, Luciferase, and HSV-TK A168H (22). transgenes were generated by amplifying the respective coding sequences (CDS) and cloning them into the scAAV-CMV-GFP backbone (Addgene #32396) using BamHI and NotI restriction sites. Bicistronic lentiviral vector (Luciferase-IRES-Puro) was generated by amplifying the Luciferase CDS and cloning it into the Lv-Ef1a-IRES-Puro vector (Addgene #85132) using BamHI and EcoRI restriction sites. Finally, tricistronic lentiviral plasmid encoding for GD2 synthase (GD2S), GD3 synthase (GD3S), and a puromycin resistance gene was clone starting from TetO-*Ngn2*-P2A-EGFP-T2A-Puro vector (Rubio et al, 2016). In brief, the cassette containing the constitutive promoter Ef1α together with GDS2-P2A-GD3S was synthetized (Genewiz) and cloned with BamHI and XbaI.

### Post-production coupling protocol

#### AAV production and purification

AAV replication-incompetent, recombinant viral particles were produced in 293 T cells, cultured in Dulbecco Modified Eagle Medium high glucose (Merck) containing 10% fetal bovine serum (Merck), 1% non-essential amino acids (Gibco), 1% sodium pyruvate (Merck), 1% glutamine (Merck) and 1% penicillin/streptomycin (Merck). Cells were split every 3–4 days using Trypsin 0.25% (Merck). Recombinant viral particles were produced in 293T cells by polyethylenimine (PEI, Polyscience) co-transfection of three different plasmids: transgene-containing plasmid (scAAV), packaging plasmid for rep and cap (AAV9-W503A-SpyTag) genes, pHelper (Agilent). The cells and supernatant were harvested at 120 h. Cells were lysed in hypertonic buffer (40 mM Tris, 500 mM NaCl, 2 mM MgCl_2_, pH=8) containing 100 U/ml Salt Active Nuclease (SAN, Arcticzymes) for 1 h at 37 °C, whereas the viral particles present in the supernatant were concentrated by precipitation with 8% PEG8000 (Polyethylene glycol 8000, Merck) and then added to supernatant for an additional incubation of 30 min at 37 °C. In order to clarify the lysate cellular debris were separated by centrifugation (4000×* g*, 30 min). The viral phase was isolated by iodixanol step gradient (15%, 25%, 40%, 60% Optiprep, Merck) in the 40% fraction and concentrated in PBS (Phosphate Buffer Saline) with 100 K cut-off concentrator (Amicon Ultra15, Merck). Virus titers were determined using AAVpro© Titration Kit Ver2 (TaKaRa).

#### αGD2-SC protein purification

HEK293T cells were cultured in DMEM (Merck) supplemented with 10% Fbs (Gibco) and 4 mM L-Gln (Merck). To express αGD2-SpyCatcher (αGD2-SC) fusion protein, HEK293 cells were transiently transfected with the plasmid encoding histidine-tagged αGD2-SC, using polyethyleneimine (PEI, Merck). After 24 h transfected cells were washed with phosphate buffered saline (PBS) and cultured in the serum-free Pro293a-CDM media (Lonza) supplemented with 4 mM L-Gln for an additional 72 h. Conditioned media was harvested, centrifuged to remove cell debris, and concentrated ten times using centrifugal filter units with a 10 kDa cutoff (Amicon, Merk Millipore). Concentrated conditioned media was dialyzed in 20 mM sodium phosphate, 0.5 M NaCl, pH 7.4. His-tagged αGD2-SC was purified from the conditioned media by affinity chromatography on Ni Sepharose 6 Fast Flow (Merck). Ni Sepharose-adsorbed αGD2-SC was eluted in 20 mM sodium phosphate, 0.5 M NaCl, pH 7.4 containing 500 mM imidazole and dialyzed in PBS. Purified αGD2-SC was analyzed by 10% SDS-PAGE following Coomassie staining and by immunoblot. In immunoblot experiments αGD2-SC was detected using an anti-V5 antibody (cat #R960-25, Thermo Scientific).

#### AAV–αGD2-SC incubation for coupling

For coupling experiments, purified AAV9m-ST and αGD2-SC protein were incubated at different ratios (1:200, 1:400, 1:600, 1:800) in cold room conditions (4 °C) for 24 h prior to downstream analyses. Following incubation, samples were analyzed by Western blot to assess conjugation efficiency.

### Co-expression coupling protocol

AAV replication-incompetent, recombinant viral particles were produced as described before. For targeted capsids, 1.5 μg of the SpyCatcher-αGD2 plasmid in 10-cm dish was added together with the other three plasmids. Mosaic capsids were generated by co-transfecting rep/cap plasmids (AAV9-W503A) containing or lacking the SpyTag insertion at defined ratios (1:3 and 1:7).

All Cultures were fixed for immunofluorescence staining (4% PFA, 4 °C, 30′) or for FACS analysis (4% PFA, 4 °C, 10′). In the former case cell were washed, resuspended (PBS, 2% FBS) and filtered (70 mm), and ZsGreen fluorescence was analyzed by CytoflexS.

### Deglycosylation of αGD2-SC

To remove N- and O-linked glycans from the αGD2-SC protein, the Protein Deglycosylation Mix II (NEB) was used, which contains a combination of enzymes capable of cleaving a broad range of glycan structures. Deglycosylation was performed following the manufacturer’s instructions. Briefly, αGD2-SC was incubated with the enzyme mix in the provided reaction buffer for 1 h and 16 h at 37 °C. Treated protein samples were subsequently analyzed by western blot to assess the efficiency of glycan removal and to compare the electrophoretic mobility to that of untreated protein.

### Western blot

Western Blots analysis was performed on purified AAV vector preparations using 1 × 10^9^ vg AAV proteins were denaturated at 95 °C in Laemmli buffer containing SDS, β-mercaptoethanol and dithiothreitol (DTT). Proteins were then separated via SDS-PAGE using 8% polyacrylamide gel and then transferred to nitrocellulose membranes. Membranes were incubated overnight at 4 °C with the following primary antibodies in 1× PBST with 5% w/v nonfat dry milk: mouse anti-AAV VPs (1:250; Arp1), mouse anti-V5 (1:1000; ThermoFisher Scientific). Subsequently, membranes were incubated with the corresponding horseradish peroxidase (HRP)-conjugated secondary antibodies (1:10,000; Dako). The signal was then revealed with a chemiluminescence solution (ECL reagent, RPN2232; GE Healthcare) and detected with the ChemiDoc imaging system (Bio-Rad).

### Lentiviral vector production

Lentiviral replication-incompetent, VSVg-coated lentiviral particles were packaged in HEK293T cells (Rubio et al, 2016). Cells were transfected with 30 μg of vector and packaging constructs, according to a conventional CaCl_2_ transfection protocol. After 30 h, medium was collected, filtered through 0.44 μm cellulose acetate and centrifuged at 20,000 rpm for 2 h at 20 °C in order to concentrate the virus.

### Cell cultures

Human NB cell lines were cultured in plastic-adherence condition in RPMI 1640 medium (Roswell Park Memorial Institute 1640 Medium, Gibco) containing 10% fetal bovine serum (FBS, Merck), 1% Pen/Strept (Merck), 2 mM Glutamine (Merck), 1% non-essential amino acids (MEM NEAA, ThermoFisher Scientific), 1% sodium pyruvate solution (Merck) and passaged twice a week using Trypsin-EDTA solution (Merck-Aldrich). All cell cultures were kept in humidified atmosphere of 5% CO2 at 37 °C under atmospheric oxygen conditions and regularly checked for mycoplasma. Cell lines were authenticated using Short Tandem Repeat (STR) fingerprint (CD-Genomics).

For the generation of HEK293T-GD2⁺ cells, HEK293T cells were maintained in DMEM (Thermo Fisher Scientific) supplemented with 10% fetal bovine serum (FBS; Merck), 1% penicillin–streptomycin (Merck), 2 mM L-glutamine (Merck), 1% non-essential amino acids (MEM NEAA; Thermo Fisher Scientific), and 1% sodium pyruvate (Merck). Cells were passaged twice weekly using Trypsin-EDTA (Merck). Briefly, HEK293T cells were plated and transduced with a tricistronic lentiviral vector encoding GD2 synthase (GD2S), GD3 synthase (GD3S), and a puromycin resistance gene. Transduced cells were maintained under puromycin treatment to obtain a stable GD2⁺ cell population.

For GD2 expression evaluation, cells were fixed in 4% PFA for 10 min at 4 °C, washed, resuspended in PBS containing 2% FBS, and incubated with anti-GD2 antibody (BD Biosciences) and filtered through a 70 µm cell strainer. Finally, GD2 expression was analyzed using a CytoFLEX S flow cytometer (Beckman Coulter).

### In vitro AAV transduction assays

Cells were seeded in 24-well plates at a density of 2.5 × 10^4^ cells/well in 500 μL of complete medium. Depending on the experiment, cells were plated either directly onto tissue culture plates or onto glass coverslips for immunofluorescence analyses. Twenty-four hours later, cells were transduced with AAV-ST, AAV-STITCHαGD2, AAV-STITCHαHER2, or AAV-STITCHαGFP vectors at a multiplicity of infection (MOI) of 400,000 vg/cell. Twenty-four hours post-transduction, the medium was replaced with fresh medium. For patient-derived samples, tumor explants were dissociated using the Tumor Dissociation Kit (Miltenyi Biotec). Resulting single-cell suspensions were cultured in complete RPMI-1640 medium, whereas HEK293 cells were maintained in DMEM (see “Cell culture” section), and subsequently transduced as described above. Seventy-two hours post-transduction, cells were fixed with 4% paraformaldehyde (PFA) either for immunofluorescence (30 min at 4 °C) or flow cytometry analysis (10 min at 4 °C). For immunofluorescence analyses, cells were washed three times with PBS and incubated overnight at 4 °C with anti-GD2 primary antibodies (Abcam). The following day, cells were washed with PBS and incubated for 1 h at room temperature with Alexa Fluor-conjugated secondary antibodies and DAPI-containing blocking solution. After additional washes, samples were mounted using fluorescence mounting medium (Dako), and images were acquired using a Nikon Eclipse 600 fluorescence microscope. ZsGreen reporter expression was detected through its endogenous fluorescence without antibody amplification.

For flow cytometry analyses, cells were washed, resuspended in PBS containing 2% FBS, filtered through a 70-μm cell strainer, and analyzed using a CytoFLEX S cytometer (Beckman Coulter). Depending on the experiment, transduction efficiency was evaluated either by direct detection of endogenous ZsGreen fluorescence alone or in combination with GD2 immunostaining (BD Bioscience).

### Immunohistochemistry

Subcutaneous tumor and metastatic liver tissues were harvested, fixed in 4% PFA for 24–48 h at 4 °C, and subsequently processed for paraffin embedding. Formalin-fixed paraffin-embedded (FFPE) tissues were sectioned at 5 μm using a microtome and mounted onto glass slides. For histological evaluation, sections were deparaffinized in xylene, rehydrated through a graded ethanol series, and stained with haematoxylin and eosin (H&E) following standard protocols. For immunohistochemical analysis, antigen retrieval was performed by heating the slides in citrate buffer (pH 6.0), followed by blocking in appropriate serum. Sections were incubated overnight at 4 °C with an anti-Ki67 primary antibody (Abcam), then with a biotinylated secondary antibody and an avidin-biotin complex (ABC, Abcam) detection system. Signal was visualized using diaminobenzidine (DAB, Merck). After staining, slides were dehydrated, cleared in xylene, and coverslipped using a permanent mounting medium. Images were acquired using a brightfield microscope.

### Organotypic brain cultures

Organotypic brain slice cultures were prepared from adult C57BL/6 wild-type mice. Brains were extracted and sectioned into 300 μm-thick coronal slices using a vibratome. Slices were placed onto porous membrane inserts within 6-well culture plates and maintained in culture medium composed of Neurobasal (Thermo Fisher), 1× B27 supplement (Thermo Fisher), 1× 30% glucose (Merck), and 1× penicillin/streptomycin and glutamine (Merck). Slices were then transduced overnight with AAV-STITCHαGD2 at a dose of 2 × 10e10 vg/well. The following day, slices were fixed in 4% paraformaldehyde (PFA), embedded in OCT compound, sectioned at 30 μm, and stained with an anti-ZsGreen antibody. Images were captured with Maving RS-G4 Confocal and Nikon Eclipse 600 fluorescent microscope.

### Virus binding assays

Virus binding assays were performed as described in Nonnenmacher and Weber (2011). In brief, AAVs were added to SH-SY5Y at 40,000 MOI for 1 h at 4 °C. After, 3× PBS washes, cells were lysed in 1% NP-40 buffer, and viral DNA was extracted and quantified using AAVpro© Titration Kit Ver2 (TaKaRa).

### Total DNA isolation for qRT-PCR

Total DNA was extracted from primary neurons, animal tissues and dissected subcutaneous xenograft tumor using the QIAamp DNA Micro Kit (QIAGEN). Quantification of vector-derived transgene copies was performed by qRT-PCR using primers targeting the ZsGreen sequence, as described in Giannelli et al (2024). DNA levels were normalized to a single-copy reference gene, *LMNB2*, amplified from genomic DNA. Data were expressed either as fold change relative to the AAV9m-ST control group (Fig. 1G–H) or as 2^ΔCt values for the comparison between AAV9 and AAV-STITCHαGD2 (Fig. 2B).

### In vitro cytotoxicity assay

Citotoxicity was assessed via LIVE/DEAD™ Viability/Cytotoxicity Kit (ThermoFisher). Briefly, 25,000 SH-SY5Y cells were plated on glass coverslips in 500 μL of media in a 24-well plate. The following day, cells were transduced with the viral vector (AAV-STITCHαGD2) followed by a daily exposition to the prodrug 8ug/ml Ganciclovir (Merck) for 72 h or to DMSO as negative control. Then staining solution was prepared by adding 5 µL of 4 mM Calcein AM and 20 µL of 2 mM Ethidium Homodimer III (EthD-III) to 10 mL of PBS. Cell media was removed before addition of 200 µl/well of staining solution, incubated for 30 min at RT. Then, cells were mounted with fluorescent mounting medium (Dako). Images were captured with a Nikon Eclipse 600 fluorescent microscope.

Alternatively, cells were resuspended in PBS containing 2% FBS, stained with the Live/Dead Fixable Near-IR Dead Cell Stain Kit (Thermo Fisher Scientific) and fixed in 4% PFA for 10 min at 4 °C, according to the manufacturer’s instructions. Samples were then filtered through a 70 µm cell strainer, and live and dead cells were analyzed using a CytoFLEX S flow cytometer (Beckman Coulter).

### Antibody competition assay

HEK293-GD2+ cells were seeded at a density of 1 × 10^4^ cells/well in 96-well plates. Twenty-four hours later, cells were incubated for 1 h with serial dilutions (1:3) of anti-GD2 antibody in DMEM medium. Antibody amounts per well were 500 ng, 167 ng, and 55.5 ng, respectively. Following incubation, cells were washed once with DMEM and subsequently transduced overnight with AAV-STITCHαGD2 carrying a CMV-Luc reporter cassette at a multiplicity of infection (MOI) of 40,000 vg/cell. Twenty-four hours post-transduction, luciferase activity was assessed using the IVIS imaging system following the addition of 20 µl D-luciferin solution (15 mg/ml, Merck).

### AAVR silencing and evaluation of AAV-STITCHαGD2 transduction

HEK293-GD2+ cells were seeded either at a density of 1 × 10^4^ cells/well in 96-well plates or 5 × 10^4^ cells/well in 24-well plates. Twenty-four hours later, cells were transfected using Lipofectamine LTX (Thermo Fisher Scientific) with the pSUPER plasmid expressing an shRNA targeting AAVR (sequence: 5′-GCTGAGATGGTAGCATAGAAA-3′; Merck) or with an scramble (sequence: 5’-CCTAAGGTTAAGTCGCCCTCG-3’ control plasmid). Twenty-four hours post-transfection, cells plated in 96-well plates were transduced overnight with AAV-STITCHαGD2 carrying a CMV-Luc reporter cassette at a multiplicity of infection (MOI) of 40,000 vg/cell.

The following day, luciferase activity was quantified using the IVIS imaging system following the addition of 20 μL D-luciferin solution (15 mg/mL, Merck).

In parallel, total RNA was extracted from cells plated in 24-well plates using NucleoZOL (Macherey Nagel) and analyzed by qRT-PCR to assess AAVR knockdown efficiency. AAVR expression was quantified using the following primers: forward 5′-TGGCTCCCTCCTACAGTTATG-3′ and reverse 5′-GGCAGGGTTATCTGGACACT-3′. Expression levels were normalized to 18S rRNA using the following primers: forward 5′-GTAACCCGTTGAACCCCATT-3′ and reverse 5′-CCATCCAATCGGTAGTAGCG-3′.

### Animals

C57BL/6 N and immunodeficient NSG mice were employed at age between 2 and 4 months of both sexes. Animals were maintained at San Raffaele Scientific Institute Institutional mouse facility (Milan, Italy) in micro-isolators under sterile conditions under a 12 h dark/light cycle, with controlled temperature (25 °C) and relative humidity (50–60%) and with free access to food and water. All experiments were performed in accordance with protocols approved by the internal Institutional Animal Care and Use Committee (IACUC #1455, Ospedale San Raffaele, Milan, Italy) and reported to the Italian Ministry of Health according to the European Commission Council Directive 2010/63/EU.

### In vivo NB models generation

#### Pseudometastatic model

SH-SY5Y cells were resuspended in sterile PBS at a concentration of 1 × 10e6 cells/ml and then 100 µl (100.000 cells) were injected into the tail vein of NSG mice (NOD.Cg-*Prkdc*^*scid*^ *Il2rg*^*tm1Wjl*^/SzJ) using 0.3 ml syringes. The extent of liver metastasis was then assessed through MRI imaging at 2 and 3 weeks post-injection to evaluate metastatic burden, using tumor size as the primary endpoint rather than lesion number, as advanced-stage metastatic foci frequently merge into larger masses, making accurate quantification of individual lesions unreliable.

#### Subcutaneous model

In total, 3 × 10^5^ SH-SY5Y cells were seeded in a well of a six multi-well plate and transduced with LV-Luc-ires-Puro (10^8^ IFU/ml). After 72 h of puromycin selection, we expanded the cells in a T25 flask. SH-SY5Y-Luc+ cells were then resuspended in a 50% Matrigel (Matrigel growth factor reduced, Corning) solution in sterile PBS at a concentration of 2 × 10e7 cell/ml. Then 100 µl per mouse of solution were subcutaneously injected in the right flank of NSG mice (NOD.Cg-*Prkdc*^*scid*^ *Il2rg*^*tm1Wjl*^/SzJ) using 0.3 ml syringes. Evaluation of tumor burden progression occurred through BLI imaging (IVIS spectrum) at multiple timepoints from the 2nd week post injection onward trough intraperitoneal luciferin injection.

#### Patient-derived xenograft (PDX) model

Patient-derived cells were kindly donated by Childhood Cancer Repository (https://www.cccells.org/). Informed consent was obtained from all subjects and experiments conformed to the principles set out in the WMA Declaration of Helsinki and the Department of Health and Human Services Belmont Report. Childhood Cancer Repository explicitly states that all distributed samples are de-identified and covered by its own ethics approval and consent procedures.

In total, 50 × 10e6 were injected subcutaneously in NSG mice (NOD.Cg-*Prkdc*^*scid*^ *Il2rg*^*tm1Wjl*^/SzJ) using 0.3 ml syringes. After 2 weeks, mice were injected intravenously with AAV-STITCHαGD2 expressing Luciferase to evaluate tumor burden progression through BLI imaging (IVIS spectrum) at multiple timepoints onward through intraperitoneal luciferin injection.

### In vivo AAV-STITCHαGD2 transduction

C57BL/6 wild-type mice and pseudometastatic mouse models (generated as described in the previous section) were used for systemic AAV administration. In pseudometastatic mice, AAV injection was performed 2 weeks after SH-SY5Y cell inoculation. Mice were injected systemically via the tail vein with the ZsGreen-encoding AAV-STITCHαGD2 vector (1 × 10e11 vg/mouse).

Seventy-two hours after tail vein injection, mice were sacrificed and transcardially perfused with saline solution. The following organs were collected and post-fixed in 4% PFA in PBS for 48 h: liver (including both stromal and metastatic regions), heart, kidney, skeletal muscle, lung, cortex, cerebellum, and dorsal root ganglia (DRGs).

After fixation, tissues were immersed in a cryoprotective solution (30% sucrose in PBS) and processed for cryostat sectioning and immunofluorescence analysis. Alternatively, dissected tissues were used for DNA extraction as described in the “Total DNA isolation for qRT-PCR” section.

### Tissue immunofluorescence staining

Tissues were embedded in optimal cutting temperature (OCT) compound and frozen on dry ice, then sectioned using a cryostat. Fifty-micrometer-thick sections of each collected organ were mounted on glass slides, rinsed in PBS, and incubated for 1 h at room temperature in blocking solution containing 10% donkey serum (Merck) and 3% Triton X-100 (Merck) to block nonspecific binding sites. Sections were then incubated overnight at 4 °C with the primary antibody diluted in the same blocking solution. After three washes with PBS, sections were incubated for 1 h at room temperature in blocking solution containing DAPI (1:1000; Merck) and Alexa Fluor 488-conjugated anti-rabbit secondary antibody (1:1000; Thermo Fisher Scientific). Following three additional PBS washes, sections were mounted using fluorescent mounting medium (DAKO). Images were captured using a Nikon Eclipse 600 fluorescent microscope. Primary antibodies used included rabbit Living Colors® Full-Length ZsGreen Polyclonal Antibody (1:500; Takara Bio), or, for DRGs, MAP2 (1:1000; Thermo Fisher Scientific) and ATF3 (1:500; Abcam).

### In vivo cytotoxicity essays in pseudometastatic mouse model

Pseudometastatic mouse models were injected systemically (tail vein) with 1 × 10e10 vg/mouse of AAV-STITCHαGD2 or AAV-ST (control) expressing HVS-TK 2 weeks after SH-SY5Y inoculation. Then all mice were intraperitoneally injected with 30 mg/kg of Ganciclovir (Merck) for 7 consecutive days. At the end of the treatment, tumor burden evaluation was performed through MRI (pseudometastatic model) to assess changes in tumor growth between experimental groups. MRI imaging analysis were performed using “MIPAV” software to quantify both metastasis number and dimension (mm^3^).

Subsequently, a subset of animals was used as organ donors for histological and immunohistochemical analyses of liver metastases.

### In vivo cytotoxicity essays in subcutaneous xenograft mouse model

Subcutaneous xenograft mouse models were generated by inoculation of SH-SY5Y cells. Two weeks later, mice were systemically injected with 1 × 10e10 vg/mouse of AAV-STITCHαGD2 expressing HVS-TK, treated with TOpotecan + TEMozolomide (TOTEM) as described in Capasso et al (2024), or received the combination of both treatments. Control animals were injected with 1 × 10e10 vg/mouse of AAV-ST expressing HVS-TK. All AAV-treated mice were intraperitoneally injected with 30 mg/kg of Ganciclovir for 7 consecutive days. Tumor burden was evaluated by IVIS bioluminescence imaging (BLI), and quantitative analyses were performed using *Aura* software to measure photon intensity from the tumor mass.

Results are reported either as fold change in tumor growth relative to baseline, comparing treated mice (AAV-STITCHαGD2 expressing HVS-TK) to controls (AAV9m-ST expressing HVS-TK) (Fig. 4C), or as tumor growth at each time point (baseline, day 4, 7, 14) normalized to the corresponding control group to illustrate relative tumor reduction (Fig. 7B).

Subsequently, tumor diameters were measured upon sacrifice, when subcutaneous tumors reached ~1 cm in diameter. At the same time point, treated mice were also sacrificed for immunohistochemical analyses.

### In vivo cytotoxicity essays in patient-derived xenograft (PDX) mouse model

Patient-derived xenograft (PDX) mouse models were injected with 1 × 10e10 vg/mouse of AAV-STITCHαGD2 or AAV-ST (control), both expressing HVS-TK, 1 week after luciferase inoculation. Mice were then intraperitoneally injected with 30 mg/kg of Ganciclovir for 7 consecutive days. Tumor burden was assessed by IVIS BLI as described above.

### Hematology and liver biochemistry profile

Blood samples were collected from AAV-STITCHαGD2-treated and control mice by retro-orbital venipuncture under isoflurane anesthesia, 1 week (acute) and two months (chronic) after viral inoculation. Approximately 100 μL of blood was collected using EDTA-coated capillary tubes, and complete blood counts were determined using an IDEXX ProCyte analyzer.

For serum collection, blood samples were allowed to clot for 30 min at room temperature and then centrifuged at 13,000×*g* for 10 min at 4 °C. The supernatant (serum) was collected and stored at −80 °C until analysis. Serum levels of alanine aminotransferase (ALT), aspartate aminotransferase (AST), and alkaline phosphatase (ALP) were measured using an ILab Aries automated clinical chemistry analyzer.

### Evaluation of peripheral neurotoxicity in dorsal root ganglia

Adult C57BL/6 wild-type mice were systemically injected via tail vein with TK-encoding AAV-STITCHαGD2 vectors (1 × 10^11^ vg/mouse). Following vector administration, mice received ganciclovir (GCV) treatment for 2 weeks using the same regimen adopted in tumor-bearing models.

To assess potential peripheral neurotoxicity, mice underwent behavioral and functional analyses including Rotarod and Hot Plate tests, as well as sensory nerve conduction studies of the tail nerve. At endpoint, dorsal root ganglia (DRG) were collected and processed for immunofluorescence analyses to evaluate neuronal integrity and viability.

### Unilateral sciatic nerve injury

Adult C57/B6 mice were anesthetized with isoflurane (2–3% in oxygen) and placed on a heating pad to maintain body temperature. The left sciatic nerve was exposed through a small incision in the mid-thigh region, carefully separated from surrounding tissues, and crushed using fine forceps for 30 s to induce a standardized injury. The crush site was marked with a 9-0 nylon suture for later identification. The incision was then closed in layers using absorbable sutures for the muscle and wound clips for the skin. Postoperative care included daily monitoring of animal welfare. The contralateral sciatic nerve was left intact and served as an internal control. Animals were sacrificed at defined time points post-injury for immunohistochemistry.

### Rotarod

Mice were assessed on an accelerating rotarod (Ugo-Basile, Stoelting). Revolutions per minute (rpm) were set at an initial value of 5 with a progressive increase to 40 rpm across the 5 min test session. Animals underwent 2 days of training followed by 3 consecutive days of testing, with each day consisting of three trials. Latency to fall was automatically recorded by the rotarod timer.

### Hot plate test

Hot plate test was performed as described in Di Pizio et al (2023). In brief, mice were tested for heat sensitivity at temperatures of 52 °C. Each mouse was placed in a Plexiglas box on the heated metal surface and the latency to initiate a nociceptive response (licking, paws shaking, jumping) was recorded.

### Sensory nerve conduction studies

Sensory nerve conduction studies were performed to evaluate potential peripheral neurotoxicity following systemic administration of AAV-STITCHαGD2-TK + GCV. Untreated control animals were analyzed in parallel. Briefly, mice were anesthetized and sensory nerve action potentials (SNAPs) were recorded from the tail nerve using a bipolar electrode configuration as described in PMID: 20305879.

Electrophysiological recordings were obtained before treatment initiation (baseline) and repeated after treatment completion. The following parameters were analyzed: SNAP amplitude (µV) and sensory nerve conduction velocity (m/s).

### Graphics

Figures 2A, 3A, 4A, 6A, 7A, EV3A, EV5A, EV6A, EV7A and EV8D were created with BioRender.com.

### Statistics

Values are expressed as mean ± standard deviation as indicated. All statistical analysis was carried out in GraphPad Prism 8.0, using *t* Test, one-way ANOVA with Tukey’s post hoc test, two-way ANOVA with Sidak’s multiple comparisons correction and Mantel–Cox test for survival curves. Number of replicates for statistical testing were indicated in corresponding figure legends.

Animals were randomly assigned to the different experimental groups after tumor establishment. Regarding experimental blinding, MRI data were analyzed by an independent operator not involved in the study execution. For IVIS analyses, all experimental groups were always included in the same imaging sessions and processed in parallel under identical conditions, including controls when possible, to minimize batch effects and operator bias.

Electrophysiological data were analyzed using a two-way repeated-measures design with treatment and time as variables. All experimental samples and biological replicates were included in the analyses. No animals, samples, experimental groups or data points were excluded after data collection. Technical measurements were excluded only when a predefined technical failure occurred, such as unsuccessful sample processing, inadequate assay quality or failure to meet the established quality-control criteria. No data were excluded on the basis of the magnitude or statistical significance of the results.

## Supplementary information

Peer Review File

Source data Fig. 1

Source data Fig. 2

Source data Fig. 4

Source data Fig. 5

Source data Fig. 6

Source data Fig. 7

Expanded View Figures

## Data Availability

The raw image files associated with this study have been deposited in the BioImage Archive repository under accession number S-BIAD3835. The source data of this paper are collected in the following database record: biostudies:S-SCDT-10_1038-S44321-026-00520-7.
