# Supplementary material for: Targeted AAV gene therapy for neuroblastoma via direct capsid-antibody coupling
Source: EMBO Mol Med. 2026 Sep 12;18(10):4117–52. doi: 10.1038/s44321-026-00520-7 (PMC13658805; doi:10.1038/s44321-026-00520-7)
Supplement: Supplementary file 1 [file 44321_2026_520_MOESM2_ESM.pdf]

EMBO Molecular Medicine

Targeted AAV gene therapy for neuroblastoma via direct capsid-antibody coupling

Mirko Luoni, Stefano Parracino, Serena Giannelli, Francesca Morosi, Elisa Ventura, Agostina Di Pizio, Sharon Muggeo, Angelo Iannielli, Silvia Sicardi, Tamara Canu, Tommaso Russo, Antonio Esposito, Ubaldo Del Carro, Fabio Pastorino, and Vania Broccoli

Corresponding author: Vania Broccoli (broccoli.vania@hsr.it)

Review Timeline:

Submission Date:	11th Dec 25
Editorial Decision:	27th Jan 26
Revision Received:	27th May 26
Editorial Decision:	30th Jun 26
Revision Received:	24th Jul 26
Editorial Decision:	29th Jul 26
Revision Received:	6th Aug 26
Accepted:	28th Aug 26

Editor: Lise Roth

Transaction Report:

(Note: With the exception of the correction of typographical or spelling errors that could be a source of ambiguity, letters and reports are not edited. Depending on transfer agreements, referee reports obtained elsewhere may or may not be included in this compilation. Referee reports are anonymous unless the Referee chooses to sign their reports.)

27th Jan 2026

Dear Dr. Broccoli,

Thank you for submitting your manuscript to EMBO Molecular Medicine. Please accept my apologies for the delay in responding, which is due to the time it took to find referees during the holiday season. We have now received the reports from the two referees who evaluated your manuscript. As you will see from the enclosed reports, while the referees acknowledge the potential interest of the findings, they also raise concerns relating to the timepoint chosen for analysis, potential off-target effects, the choice of therapeutic regimen and transduction efficiency.

Based on the nature of the concerns and the amount of time and work that would be needed to address them, considering also that at EMBO Press we encourage a single round of revisions in a limited time frame, we prefer to return the manuscript to you at this point with the decision that we cannot offer to publish it.

However, given the potential interest of the findings, we would consider a new manuscript on the same topic if you could provide data that fully addresses the referees' concerns.

To be completely clear, however, I would like to stress that if you were to send a new manuscript, this would be treated as a new submission rather than a revision and would be reviewed afresh, in particular with respect to the literature and the novelty of your findings at the time of resubmission. If you decide to follow this route, please make sure you nevertheless upload a letter of response to the referees' comments.

I am sorry that I could not bring better news this time and hope that the referees' comments are helpful in your continued work in this area.

With kind regards,

Lise Roth

Lise Roth, Ph.D
Senior Editor
EMBO Molecular Medicine

***** Reviewer's comments *****

Referee #1 (Comments on Novelty/Model System for Author):

I wanted to stress that the use of a non-integrative vector for a suicide strategy may result in loss of genomes due to tumoral cell replication. Efficacy assessment is mainly done at 1 or 2 weeks after vector infusion. When survival was evaluated, there was only a delayed tumoral development, indicating that the vector was indeed diluted. This short-term follow-up represents a major limitation of the model used and undermines the translational potential of the approach.

Referee #1 (Remarks for Author):

The manuscript from Luoni and colleagues reports a rational engineering approach of an AAV vector to redirect the targeting to neuroblastoma cells and to avoid tissue off targeting. They demonstrate the specificity of the approach in vivo by showing targeting of tumors derived from grafting of cultured cells and of human explants. The major criticism is related to the choice of the timepoint of analysis. AAV vectors are non-integrative and in rapidly dividing cells transgene expression is rapidly loss. The authors show mainly efficacy data at one or two weeks, a period of time where AAV is at the maximum of efficacy. When survival was evaluated, treatment with the rational engineered AAV resulted in an increase in survival of 3-6 weeks. This is consistent with a dilution of the vector genome and a loss of efficacy. As correctly stated by the authors, a second administration of the vector may be possible in the next future. However, this is not very easy in the short-term and repeated administration of AAVs is certainly out of the reach of current technologies. As such, the translational potential of the presented approach remains limited for tumors.
I also have few comments that need attention:
1. In the abstract, the authors mentioned targeted tumor cell elimination. However, in the only study they performed in the long-term, treated mice only showed a delayed death, indicating that tumors were not eliminated but just slowed-down. The general conclusions on the translational potential of the approach must be tuned-down.
2. While the authors are showing no off-target in most of the peripheral tissues, DRG are targeted by the vectors. DRG are a

known off-target tissue associated with toxicities likely due to transgene expression and/or capsid overloading. In mice this toxicity can hardly be seen and the authors cannot claim specificity and safety only by using a mouse model.
3. When the rational engineered AAV is combined with TOTEM, the authors claim an additive effect of the two treatments. However, there is no statistical analysis supporting this claim.
4. In figure 6B and C the authors evaluate the size of the tumors showing a decreased size. Is there also a decrease in the number of tumors? This is important for the interpretation of the survival data. Also in the survival graph statistics are not indicated although it looks like the analyses were performed.
5. The authors claim that by their approach they do not have a decrease in production titers. However, it looks from the data showed in figure S1G that there is a decrease that is hard to evaluate due to the lack of individual datapoints. It is also difficult to see differences due to the choice to represent the data with a log scale. I suggest to show individual values and report the data as vg/plate instead of the concentration of the final vector production (because the final volume may change).

Referee #2 (Comments on Novelty/Model System for Author):

In this manuscript, Luoni et al. describe a novel strategy (AAV-STITCH) to retarget adeno-associated viral vectors by covalently coupling antibody fragments to the capsid using the SpyTag/SpyCatcher system. By introducing SpyTag into an infectivity-deficient AAV9 capsid and co-expressing SpyCatcher-fused scFvs, the authors generate antibody-decorated AAV particles. Using an anti-GD2 scFv, they demonstrate selective transduction of GD2⁺ neuroblastoma cells in vitro and in vivo, with minimal off-target transduction. Therapeutic relevance is shown by delivery of HSVtk in multiple neuroblastoma xenograft models, including a patient-derived xenograft, where significant tumor suppression and improved survival are observed.
The study addresses an important limitation of AAV gene therapy, lack of cell-type specificity, and problems with systemic administration, and proposes a potentially versatile solution. The data are generally convincing, and the manuscript is clearly written. However, several conceptual, mechanistic, and translational issues need to be addressed.

Major Comments:
1. The authors show that AAV-STITCHαGD2 selectively transduces GD2⁺ neuroblastoma cells in xenograft models and largely spares major peripheral normal organs. However, the vector clearly transduces dorsal root ganglion (DRG) neurons in vivo, consistent with known GD2 expression in this population. While the authors argue that the HSVtk/GCV system prevents toxicity in non-proliferating neurons, this safety feature does not address the broader issue of vector biodistribution. Given that AAV-STITCH vector particles should still be present in and transduce the DRGs, the presence of viral genomes and transgene expression in DRGs represents an off-target effect.
The authors should provide a more comprehensive quantitative biodistribution analysis (qPCR and/or reporter expression) across additional tissues, including CNS regions.
The authors should clarify whether low-level transduction in other GD2⁺ or GD2-low tissues might have been missed.
The Discussion should more critically address how off-target binding to normal GD2-expressing tissues might affect future therapeutic payloads that are not suicide genes.
AAV capsid modifications, including AAV9, can result in varied transduction efficiencies between species. Why didn't the authors compare with the original, unmodified AAV9 vector, for in vitro and in vivo transductions?

2. Initial transduction efficiency of non-mosaic AAV-STITCHαGD2 vectors is relatively low, and substantial optimization through mosaic capsid assembly is required to achieve functional infectivity. While the authors identify a 1:3 SpyTag-positive to SpyTag-negative ratio as optimal, it remains unclear how robust or generalisable this optimization is. Have the authors tested whether similar coupling densities are optimal for other scFvs, or is extensive re-optimisation required for each new targeting moiety? How reproducible is the coupling efficiency across independent vector preparations?
A comparison of transduction efficiency with other reported targeting strategies (e.g., peptide insertion, or chemistry approaches), or a discussion at least, would strengthen the manuscript.

3. Did the authors check HSVtk expression in the tumors before GCV initiation, how did they decide to give GCV one week after AAV9 IV administration?
GCV is typically administered at dosages ranging from 25 mg/kg to 100 mg/kg per day. Why did they use 1 mg/kg/day of GCV? This is too low! Did they use mutant HSVtk, such as HSVtk mutant SR39, that exhibits significantly higher affinity for the pro-drug GCV compared to the wild-type enzyme.
No rationale for the choice of the chemo drugs TMZ + Topotecan. Why was the combination with gene therapy done concomitantly? Some studies showed that staring with HSVtk/GCV Cancer Gene therapy then following with chemo TMZ, a few days later generated better outcome. Did the authors check if chemo drugs activate gene expression as it was previously reported that TMZ can activate promoters? No indication whether it is a CMV promoter-driven transgene cassette.
Figure 3: Why was the vector administered just two weeks after iv injection of MB metastatic cells? Did they check that the metastases were established before vector delivery?
Control with a unrelated ligand is missing.
Targeting tumor cells in the liver is quite obvious after IV delivery, but can the vector target the tumor cells somewhere else in the body?
In Figure 3, how does ZsGreen gene delivery compare to the original AAV9? Did the authors confirm that this is transduction of

tumor cells?

4. Inhibition experiments could be included to further show selectivity, such as combination with free antibody.

Mechanistic insight into viral entry and intracellular trafficking

While the binding assays support reduced membrane association in mosaic capsids, the downstream mechanisms remain speculative.
Additional experiments addressing viral internalization, endosomal escape, or intracellular trafficking would substantially strengthen the mechanistic foundation of the study.
Do AAV-STITCH vectors rely solely on antigen binding for entry, or do they still require canonical AAV co-receptors?

At minimum, the limitations of the current mechanistic understanding should be more clearly acknowledged in the Discussion.

5. The authors briefly discuss pre-existing AAV immunity and re-dosing limitations, but the potential immunogenicity of the SpyTag/SpyCatcher system and antibody fragments is not experimentally addressed.
Repeated treatment is crucial in cancer therapy, in particular in metastases to sustain a therapeutic response. Given the translational focus of EMBO Molecular Medicine, a discussion of immune responses against the modified capsid and scFv components is warranted.
If feasible, preliminary in vivo data assessing humoral or innate immune activation following AAV-STITCH administration would strengthen the translational relevance.

6. While the in vivo data are generally convincing, some experiments rely on relatively small cohort sizes. Please provide information on randomization and blinding.

Minor Comments
1- The terminology "infectivity-deficient" AAV9 should be more precisely defined early in the manuscript.

2- Consistent nomenclature should be used throughout (e.g., AAV-STITCHαGD2).

3- Some critical quantitative data are relegated to supplementary figures and could be moved to the main figures.

4- Minor grammatical and stylistic edits are needed throughout.

Referee #2 (Remarks for Author):

This manuscript presents an innovative and potentially impactful approach to targeted AAV cancer gene therapy, with proof-of-concept data in neuroblastoma models. However, additional work is required to address concerns related to targeting specificity, mechanistic understanding, immunogenicity, and translational strength. Addressing the points outlined above would significantly strengthen the manuscript and clarify its suitability for publication in EMBO Molecular Medicine.

As a service to authors, EMBO provides authors with the possibility to transfer a manuscript that one journal cannot offer to publish to another EMBO publication. The full manuscript and if applicable, reviewers reports are automatically sent to the receiving journal to allow for fast handling and a prompt decision on your manuscript. For more details of this service, and to transfer your manuscript to another EMBO title please click on Link Not Available

Please do not share this URL as it will give anyone who clicks it access to your account.

Referee #1 (Comments on Novelty/Model System for Author):
I wanted to stress that the use of a non-integrative vector for a suicide strategy may result in loss of genomes due to tumoral cell replication. Efficacy assessment is mainly done at 1 or 2 weeks after vector infusion. When survival was evaluated, there was only a delayed tumoral development, indicating that the vector was indeed diluted. This short-term follow-up represents a major limitation of the model used and undermines the translational potential of the approach.

Answer:
We agree with the reviewer that the use of a non-integrating AAV vector in a suicide-gene strategy results in progressive genome dilution during tumor cell proliferation, with consequently its long-term loss. However, we showed that a single treatment with the AAV-STITCHαGD2 is sufficient to sustain approximately a 60% reduction of tumor burden at 2 weeks after treatment, which resulted in a 30% increase in overall survival (median survival: 11 weeks in control mice vs 14 weeks in AAV-STITCHαGD2-TK+GCV–treated mice). These results provide evidence that this therapy can achieve a significant tumor targeting sufficient to curb the malignant tumor evolution. Thus, although this therapy is not resolutive, it exerts significant and long-term anti-tumoral effects. Moreover, other considerations support the translational potential of this approach. First, this viral therapy can be easily envisioned as an adjuvant therapy since it is highly complementary to current anti-tumor treatments such as chemotherapy, radiotherapy, immunotherapy or CAR-T cells. Second, this viral approach is independent by the proliferative stage of the tumor cells, being able to potentially infect quiescent cancer stem cells. These tumor cells have been described in NB to promote resistance to standard treatments and tumor recurrence (PMID: 31803140, 15720811). Our approach might provide a significant new way to intercept this highly malignant population of tumor cells that are hardly targeted by other current medications. Third, several strategy have been developed for repeated AAV administration with therapeutic potential: i) using capsids with limited antibody cross-reactivity, such as AAV9 and AAV5; ii) implementing immune-evasion platforms such as plasmapheresis (PMID: 38327805) or the use of engineered IgM and IgG cleaving enzymes (PMID: 38715362); iii) exploiting next generation engineered or non-mammalian AAV capsids with minimal immune response (PMID: 29055620, 41308640, 40253479). Finally, together or beyond the HSV-TK suicide system, AAV can deliver other potent therapeutic payloads, such as cytokines or immune-stimulatory factors (which are encoded by very small genes compatible with AAV packaging). In this context, transduced cells could exert a paracrine anti-tumor effect on neighboring non-transduced cells, potentially overcoming the issue of vector dilution and extending therapeutic benefit even after partial loss of the AAV genome. All together, these strategies may help mitigating anti-capsid immunity and support iterative vector administration in near future translational applications.
We have highlighted some of these important points in the Discussion section (page 13 of the new manuscript).

Referee #1 (Remarks for Author):
The manuscript from Luoni and colleagues reports a rational engineering approach of an AAV vector to redirect the targeting to neuroblastoma cells and to avoid tissue off targeting. They demonstrate the specificity of the approach in vivo by showing targeting of tumors derived from grafting of cultured cells and of human explants. The major criticism is related to the choice of the timepoint of analysis. AAV vectors are non-integrative and in rapidly dividing cells transgene expression is rapidly loss. The authors show mainly efficacy data at one or two weeks, a period of time where AAV is at the maximum of efficacy. When survival was evaluated, treatment with the rational engineered AAV resulted in an increase in survival of 3-6 weeks. This is consistent with a dilution of the vector genome and a loss of efficacy. As correctly stated by the authors, a second administration of the vector may be possible in the next future. However, this is not very easy in the short-term and repeated administration of AAVs is certainly out of the reach of current technologies. As such, the translational

potential of the presented approach remains limited for tumors. I also have few comments that need attention:

1. In the abstract, the authors mentioned targeted tumor cell elimination. However, in the only study they performed in the long-term, treated mice only showed a delayed death, indicating that tumors were not eliminated but just slowed-down. The general conclusions on the translational potential of the approach must be tuned-down.

Answer:

We thank the reviewer for this important comment and agree that the current study has limitations regarding long-term efficacy in rapidly proliferating tumors. We have therefore revised the abstract and discussion to better reflect these limitations and to tone-down statements regarding the translational potential of the current proof-of-concept study. At the same time, we would like to emphasize that the primary objective of this work was to establish a rationally engineered and tumor-selective AAV platform capable of efficiently targeting neuroblastoma cells while minimizing off-target transduction. In this context, the observed therapeutic delay still supports the biological activity and specificity of the approach.

Importantly, as discussed in the previous response, several strategies could further improve the durability and translational applicability of this platform. We envision this approach as a complementary therapeutic modality, and the modular nature of the system could allow the delivery of immune-stimulatory factors with paracrine anti-tumor effects. In addition, although repeated AAV administration remains challenging, emerging and highly promising redosing strategies and immune-evasion technologies indicate that iterative vector delivery may become reality in a very near future.

2. While the authors are showing no off-target in most of the peripheral tissues, DRG are targeted by the vectors. DRG are a known off-target tissue associated with toxicities likely due to transgene expression and/or capsid overloading. In mice this toxicity can hardly be seen and the authors cannot claim specificity and safety only by using a mouse model.

Answer:

We thank the reviewer for this important comment and agree that DRG toxicity represents a relevant concern for systemic AAV delivery and cannot be fully addressed in mouse models alone. To strengthen this aspect of the study, we have now integrated a functional assessment by analyzing sensory nerve conduction in the tail, providing a sensitive and functional readout for the stringent evaluation of potential DRG neurotoxicity. We realized that the immunofluorescent images of the DRG viral transduction with AAV-STITCH and AAV9 were erroneously inverted in Fig. 2A. We apologize for this mistake. A similar difference in transduction between the two vectors is also confirmed by qPCR quantifications of AAV-STITCH and AAV9 vector copy numbers in DRGs from treated animals (Fig. 2C). DRG cell transduction was quantified in approximately 5% and 16% for AAV-STITCH and AAV9, respectively (Fig. 2D). Considering that DRG toxicity in humans treated with AAV9 has been rarely reported, we do not expect that this eventuality can restrain the translational potential of AAV-STITCH. Moreover, several studies have demonstrated that improvements in vector design can further mitigate DRG-associated toxicity, including the use of epitope-dependent transgene suppression or degradation strategies mediated by miRNA regulation, as previously reported for the reduction of AAV-induced DRG toxicity in non-human primates (PMID: 33177842).

3. When the rational engineered AAV is combined with TOTEM, the authors claim an additive effect of the two treatments. However, there is no statistical analysis supporting this claim.

Answer:

We have rephrased the sentence to highlight that the combination group shows a statistically significant stronger reduction in tumor burden compared to each monotherapy groups as presented in Fig. 5B.

4. In figure 6B and C the authors evaluate the size of the tumors showing a decreased size. Is there also a decrease in the number of tumors? This is important for the interpretation of the survival data. Also in the survival graph statistics are not indicated although it looks like the analyses were performed.

Answer:
We thank the reviewer for this important point. We did not include the number of tumors as a quantitative endpoint for two main reasons. First, at later time points (e.g., 3 weeks post-engraftment), tumor burden in some animals becomes extremely high, making reliable and reproducible counting of individual metastatic lesions challenging. Second, as tumors progress, metastatic foci frequently merge into larger masses, leading to an apparent reduction in lesion number that does not reflect true tumor regression. For these reasons, we considered tumor size a more robust and biologically meaningful parameter to assess treatment efficacy in this setting. We have now clarified this point in the revised manuscript.
Regarding the survival analysis, we agree with the reviewer that statistical information was not properly indicated. This was an oversight on our part, and we have now corrected the figure by adding the appropriate statistical annotations and tests in the revised version.

5. The authors claim that by their approach they do not have a decrease in production titers. However, it looks from the data showed in figure S1G that there is a decrease that is hard to evaluate due to the lack of individual datapoints. It is also difficult to see differences due to the choice to represent the data with a log scale. I suggest to show individual values and report the data as vg/plate instead of the concentration of the final vector production (because the final volume may change).

Answer:
We agree with the reviewer that the original representation may have limited accurate interpretation of the production yields due to the use of normalized concentrations and the absence of individual data points. In the revised figure, we have expressed the data as vg/plate, confirming that this approach does not affect viral production yield (New Fig. S1G).

Referee #2 (Comments on Novelty/Model System for Author):
In this manuscript, Luoni et al. describe a novel strategy (AAV-STITCH) to retarget adeno-associated viral vectors by covalently coupling antibody fragments to the capsid using the SpyTag/SpyCatcher system. By introducing SpyTag into an infectivity-deficient AAV9 capsid and co-expressing SpyCatcher-fused scFvs, the authors generate antibody-decorated AAV particles. Using an anti-GD2 scFv, they demonstrate selective transduction of $GD2^+$ neuroblastoma cells in vitro and in vivo, with minimal off-target transduction. Therapeutic relevance is shown by delivery of HSVtk in multiple neuroblastoma xenograft models, including a patient-derived xenograft, where significant tumor suppression and improved survival are observed.
The study addresses an important limitation of AAV gene therapy, lack of cell-type specificity, and problems with systemic administration, and proposes a potentially versatile solution. The data are generally convincing, and the manuscript is clearly written. However, several conceptual, mechanistic, and translational issues need to be addressed.

Answer:

We thank the reviewer for the positive assessment of our work and for recognizing both the conceptual relevance and the quality of the data presented.

Major Comments:

1. The authors show that AAV-STITCHαGD2 selectively transduces $GD2^+$ neuroblastoma cells in xenograft models and largely spares major peripheral normal organs. However, the vector clearly transduces dorsal root ganglion (DRG) neurons in vivo, consistent with known GD2 expression in this population. While the authors argue that the HSVtk/GCV system prevents toxicity in non-proliferating neurons, this safety feature does not address the broader issue of vector biodistribution. Given that AAV-STITCH vector particles should still be present in and transduce the DRGs, the presence of viral genomes and transgene expression in DRGs represents an off-target effect. The authors should provide a more comprehensive quantitative biodistribution analysis (qPCR and/or reporter expression) across additional tissues, including CNS regions. The authors should clarify whether low-level transduction in other $GD2^+$ or GD2-low tissues might have been missed.
The Discussion should more critically address how off-target binding to normal GD2-expressing tissues might affect future therapeutic payloads that are not suicide genes. AAV capsid modifications, including AAV9, can result in varied transduction efficiencies between species. Why didn't the authors compare with the original, unmodified AAV9 vector, for in vitro and in vivo transductions?

Answer:

We thank the reviewer for this thoughtful and highly relevant set of comments. We address each specific point as follows:

<u>1. DRG transduction and safety considerations</u>

We agree that DRG transduction represents an off-target biodistribution event, as GD2 is physiologically expressed in this neuronal population. However, we would like to emphasize three important considerations. First, HSV-TK-mediated killing is restricted to proliferating cells, whereas DRG neurons are post-mitotic, substantially limiting the risk of functional toxicity. Second, the degree of DRG transduction observed in our study is very low, corresponding to about 5% and three fold lower than standard AAV9 (new quantification in Fig. 2D). On this particular issue, we realized that the immunofluorescence images were inadvertently exchange between AAV-STITCH and AAV9. We apologize for this mistake. The cell quantification and the qRT-PCR data nicely align to confirm the reduced viral transduction in DRGs with AAV-STITCH respect to the standard AAV9. Third, we have now strengthened this point experimentally by adding functional assessment of sensory pathways, including tail sensory conduction analysis, which showed no detectable alterations in DRG neuronal excitability in treated animals. In addition, we have expanded the Discussion to more critically address this issue and to outline potential future detargeting strategies, including the use of proliferation-dependent promoters and miRNA-based detargeting approaches to further minimize DRG expression as described in PMID: 33177182.

<u>2. Biodistribution analysis</u>

In response to the reviewer's suggestion, we have extended our biodistribution analysis by performing additional qRT-PCR measurements in extra CNS regions, striatum and hippocampus, which further support the specificity of the vector distribution (New Fig. 2C).

<u>3. Potential off-target effects in $GD2^+$ tissues</u>

We acknowledge that DRG targeting reflects physiological GD2 expression and may be relevant for future therapeutic payloads beyond suicide genes. We have therefore expanded the Discussion to more explicitly address this limitation and to highlight that future applications will require careful payload selection and/or detargeting strategies to mitigate potential toxicity in GD2-expressing normal tissues. Notably, the observed DRG tropism is consistent with previous observations in anti-GD2 immunotherapy, where this tissue is also a known site of on-target/off-tumor effects.

4. Comparison with parental AAV9

We agree with the reviewer on the importance of including an appropriate comparator. The AAV9-W503A backbone used in this study has been previously characterized in the literature as strongly impaired in native AAV9 tropism (PMID: 22514350, 38711458). To further address this point, we have now included in vivo experiments with wild-type AAV9 (Fig. 3B), which demonstrate its inability to efficiently target metastatic lesions in our model, thereby reinforcing the added value of the retargeting strategy.

Overall, we believe these additional data and clarifications substantially strengthen the manuscript and address the raised concerns.

2. Initial transduction efficiency of non-mosaic AAV-STITCHαGD2 vectors is relatively low, and substantial optimization through mosaic capsid assembly is required to achieve functional infectivity. While the authors identify a 1:3 SpyTag-positive to SpyTag-negative ratio as optimal, it remains unclear how robust or generalisable this optimization is. Have the authors tested whether similar coupling densities are optimal for other scFvs, or is extensive re-optimisation required for each new targeting moiety?

How reproducible is the coupling efficiency across independent vector preparations?

A comparison of transduction efficiency with other reported targeting strategies (e.g., peptide insertion, or chemistry approaches), or a discussion at least, would strengthen the manuscript.

Answer

We thank the reviewer for this important point regarding robustness, generalizability, and reproducibility of the mosaic optimization strategy.

To better generalize this method, we have tested an additional independent scFv (anti-HER2), and we observed that the 1:3 SpyTag-positive to SpyTag-negative ratio also provides the best transduction performance compared to non-mosaic formulations (new Fig. S5B-C). These new data have been added to the manuscript and support the notion that a defined intermediate coupling density (approximately 3–4 scFvs per capsid) represents a broadly effective compromise between infectivity and targeting efficiency. This, in turn, suggests that only limited and rapid re-optimization may be required when switching targeting ligands.

Regarding reproducibility, we confirm that multiple independent vector preparations of AAV-STITCHαGD2 consistently showed highly comparable coupling efficiency and transduction performance, supporting the robustness of the production pipeline. We have now explicitly included this information in the Discussion.

Finally, concerning the reviewer's suggestion to compare with other targeting strategies (e.g., peptide insertion or chemical conjugation), we respectfully feel that a direct quantitative comparison would not be appropriate given the heterogeneity of platforms, capsid backgrounds, and experimental systems used across studies. Instead, we have expanded the Discussion to clarify that the goal of our approach is not to outperform all existing systems in a head-to-head manner, but rather to provide a modular, rapid, and broadly applicable platform for efficient and flexible scFv coupling to AAV capsids.

3. Did the authors check HSVtk expression in the tumors before GCV initiation, how did they decide to give GCV one week after AAV9 IV administration?

Answer:

We would like to clarify that there is a misunderstanding regarding the timing of treatment.

GCV administration was not initiated one week after AAV9 intravenous delivery, but rather started the day after vector injection, according to our experimental design. We have clarified this in the manuscript to avoid any potential confusion.

GCV is typically administered at dosages ranging from 25 mg/kg to 100 mg/kg per day. Why did they use 1 mg/kg/day of GCV? This is too low! Did they use mutant HSVtk, such as HSVtk mutant SR39, that exhibits significantly higher affinity for the pro-drug GCV compared to the wild-type enzyme.

Answer:
We thank the reviewer for raising this important point and we apologize for the confusion in the originally reported dosage.
The correct regimen used in our study is 30 mg/kg/day of GCV, not 1 mg/kg/day as inadvertently stated in the manuscript. This error likely originated from the fact that the administered amount corresponded to approximately 1 mg of GCV per mouse per day. We have now corrected this information throughout the text. Importantly, we used the TK A168H (HSV thymidine kinase mutant) variant in our system (PMID: 35892087). This mutant has been reported to exhibit improved catalytic efficiency toward ganciclovir compared to wild-type HSV-TK, which contributes to efficient prodrug activation at lower effective intracellular enzyme levels. We have clarified this point in the manuscript.

No rationale for the choice of the chemo drugs TMZ + Topotecan. Why was the combination with gene therapy done concomitantly? Some studies showed that staring with HSVtk/GCV Cancer Gene therapy then following with chemo TMZ, a few days later generated better outcome. Did the authors check if chemo drugs activate gene expression as it was previously reported that TMZ can activate promoters? No indication whether it is a CMV promoter-driven transgene cassette.

Answer:
Temozolomide and topotecan were used according to their established role as part of the standard-of-care regimens for relapsed/refractory neuroblastoma as reported in PMID: 37775116; PMID: 24021349. The concomitant administration with AAV-STITCH was chosen to maximize therapeutic pressure in a highly aggressive xenograft setting, although we agree that alternative sequential schedules may also be considered and will be discussed in the revised manuscript.
Regarding the potential interaction with vector-driven expression, we acknowledge that DNA-damaging agents such as temozolomide may, in principle, influence the activity of viral promoters such as CMV through stress- and DNA damage–related pathways. We have now clarified in the manuscript that our transgene cassette is CMV promoter-driven. However, we did not directly assess whether TMZ modulates CMV-driven expression in our system. While a potential influence cannot be excluded, the observed additive therapeutic effect of the combined treatment suggests that any such interaction, if present, is not detrimental in our experimental setting.

Figure 3: Why was the vector administered just two weeks after iv injection of MB metastatic cells? Did they check that the metastases were established before vector delivery?

Answer:
The timing of vector administration (two weeks after intravenous injection of metastatic cells) was selected based on an initial optimization of the metastatic model.
In particular, we tested different cell doses and longitudinally monitored disease progression by MRI at 2 and 3 weeks post-injection to identify a condition that provided a robust and reproducible metastatic burden suitable for therapeutic intervention.
Importantly, MRI was also used prior to vector administration to confirm the establishment of metastatic lesions under the selected experimental conditions. We have clarified this point in the revised manuscript.

Control with an unrelated ligand is missing.

Answer:
We have included in the revised manuscript a relevant control using an unrelated scFv directed against GFP. In these experiments, no appreciable increase in transduction compared to the control without scFv was observed in GD2-negative SH-SY5Y cells, as shown in the corresponding figure.

Targeting tumor cells in the liver is quite obvious after IV delivery, but can the vector target the tumor cells somewhere else in the body?

Answer:
We also have evidence that the vector specifically targets tumors outside the liver, as shown in the subcutaneous PDX model, where we observed efficient and selective transduction of tumor cells. To assess additional metastatic sites in a more comprehensive manner, an orthotopic model would be required. However, we feel that this is beyond the scope of the present study since would require the establishment of an additional and entirely new mouse model.

In Figure 3, how does ZsGreen gene delivery compare to the original AAV9? Did the authors confirm that this is transduction of tumor cells?

Answer:
We have performed additional experiments in NSG pseudometastatic models showing that wild-type AAV9 does not transduce tumor cells (Fig. 3B), in contrast to the AAV-STITCH construct. This result adds further convincing evidence on the added value of our targeting strategy.

4. Inhibition experiments could be included to further show selectivity, such as combination with free antibody.

Answer:
We have performed additional inhibition experiments using increasing concentrations of free anti-GD2 antibody. These experiments show that AAV-STITCH-mediated transduction of GD2+ HEK293 cells is significantly reduced in a dose-dependent manner, confirming that binding is antigen-specific, as now included in the revised figure S3D .

Mechanistic insight into viral entry and intracellular trafficking
While the binding assays support reduced membrane association in mosaic capsids, the downstream mechanisms remain speculative.
Additional experiments addressing viral internalization, endosomal escape, or intracellular trafficking would substantially strengthen the mechanistic foundation of the study.
Do AAV-STITCH vectors rely solely on antigen binding for entry, or do they still require canonical AAV co-receptors?

Answer:
We agree that a full dissection of intracellular trafficking mechanisms would be highly informative and valuable for further optimizing the system. As suggested by the reviewer, we provide in figure S3E-F functional evidence that AAV-STITCHαGD2 entry is not exclusively mediated by antigen binding: downregulation of AAVR significantly reduces transduction efficiency, indicating that, in addition to GD2-mediated membrane targeting, canonical AAV entry pathways are still required for productive infection.

We have also added in the Discussion that further studies will be needed to fully dissect the intracellular entry and trafficking mechanisms of this platform.

At minimum, the limitations of the current mechanistic understanding should be more clearly acknowledged in the Discussion.

Answer:
As stated above, we have included in the Discussion that a comprehensive characterization of viral entry and intracellular trafficking mechanisms will be required to fully understand the biology of the AAV-STITCH platform and to further optimize its performance.

5. The authors briefly discuss pre-existing AAV immunity and re-dosing limitations, but the potential immunogenicity of the SpyTag/SpyCatcher system and antibody fragments is not experimentally addressed.
Repeated treatment is crucial in cancer therapy, in particular in metastases to sustain a therapeutic response. Given the translational focus of EMBO Molecular Medicine, a discussion of immune responses against the modified capsid and scFv components is warranted.
If feasible, preliminary in vivo data assessing humoral or innate immune activation following AAV-STITCH administration would strengthen the translational relevance.

Answer:
We thank the reviewer for this important point regarding the immunogenicity of the AAV-STITCH system and agree that it is highly relevant for translational development.
While we did not directly assess humoral or innate immune responses against the engineered capsid or scFv components in this study, several considerations support the feasibility of repeated administration and clinical translation.
Regarding AAV immunity, multiple strategies are already being developed to enable redosing, including the use of capsids with reduced antibody cross-reactivity such as AAV9 and AAV5 (PMID: 40879499), as well as emerging immune-modulation approaches such as plasmapheresis (PMID: 38327805) and enzymatic IgG/IgM cleavage strategies (PMID: 38715362).
With respect to the SpyTag/SpyCatcher system, we note that orthogonal systems such as SnoopTag/SnoopCatcher (PMID: 26787909) show minimal sequence similarity (<16% identity), as confirmed by sequence alignment, supporting the modularity and potential immunological tunability of the platform. Finally, for the scFv component, alternative tumor-associated antigens beyond GD2, such as B7-H3 (PMID: 36327699) or GPC2 (PMID: 35852863), may further expand targeting options and facilitate clinical translation with different antibody scaffolds.

6. While the in vivo data are generally convincing, some experiments rely on relatively small cohort sizes. Please provide information on randomization and blinding.

Answer:
Animals were randomly assigned to the different experimental groups after tumor establishment.
Regarding experimental blinding, MRI data were analyzed by an independent operator, not involved in the study execution. For IVIS analyses, all experimental groups were always included in the same imaging sessions and processed in parallel under identical conditions, including controls, when possible, to minimize batch effects and operator bias.
We will clarify these procedures in the Methods section of the revised manuscript to ensure full transparency.

Minor Comments

1- The terminology "infectivity-deficient" AAV9 should be more precisely defined early in the manuscript.
The term "infectivity-deficient" AAV9 has been more precisely defined at its first occurrence in the manuscript

2- Consistent nomenclature should be used throughout (e.g., AAV-STITCHαGD2).
Nomenclature has been harmonized throughout the text.

3- Some critical quantitative data are relegated to supplementary figures and could be moved to the main figures.
Expression pattern of G2S and GD3S in various organs has been moved to the principal Figure 2B to highlight that only DRGs have sufficient expression of both GD2 producing enzymes.

4- Minor grammatical and stylistic edits are needed throughout.
We have performed a thorough revision of the manuscript to correct minor grammatical and stylistic issues throughout the text.

30th Jun 2026

Dear Dr. Broccoli,

Thank you for submitting your revised manuscript to EMBO Molecular Medicine. We have now received the reports from the original referees. As you will see below, while Referee #2 is supportive of publication, Referee #1 remains unconvinced by the translational relevance of the study in the absence of long-term in vivo experiments.

We have discussed the manuscript further with the referees and within the editorial team and have concluded that such additional long-term experiments are not essential for publication. We agree that the principal strength of the study lies in the development of the AAV-STITCH platform for the selective targeting of tumor cells.

We would therefore like to invite you to submit a further revised version of the manuscript. In particular, we ask that you more clearly acknowledge the remaining translational limitations of the current study and avoid overstating the clinical implications of the findings.

When preparing your revised manuscript, please refer to our guidelines: https://link.springer.com/journal/44321/submission-guidelines#cms-Revised-submissions. We perform an initial quality control of all revised manuscripts before re-review; failure to include requested items will delay the evaluation of your revision.

We require:

1) A .docx formatted version of the manuscript text (including legends for main figures, EV figures and tables). Please make sure that the changes are highlighted to be clearly visible.

2) Individual production quality figure files as .eps, .tif, .jpg (one file per figure). For guidance, download the 'Figure Guide PDF': https://media.springernature.com/original/springer-cms/rest/v1/content/27825798/data/v1.

3) A .docx formatted letter INCLUDING the reviewers' reports and your detailed point-by-point responses to their comments. As part of the EMBO Press transparent editorial process, the point-by-point response is part of the Review Process File (RPF), which will be published alongside your paper.

4) A complete author checklist, which you can download from our author guidelines. Please insert information in the checklist that is also reflected in the manuscript. The completed author checklist will also be part of the RPF.

5) Please note that all corresponding authors are required to supply an ORCID ID for their name upon submission of a revised manuscript.

6) It is mandatory to include a 'Data Availability' section after the Materials and Methods. Before submitting your revision, primary datasets produced in this study need to be deposited in an appropriate public database, and the accession numbers and database listed under 'Data Availability'. Please remember to provide a reviewer password if the datasets are not yet public.

In case you have no data that requires deposition in a public database, please state so in this section. Note that the Data Availability Section is restricted to new primary data that are part of this study.

7) For data quantification: please specify the name of the statistical test used to generate error bars and P values, the number (n) of independent experiments (specify technical or biological replicates) underlying each data point and the test used to calculate p-values in each figure legend. The figure legends should contain a basic description of n, P and the test applied. Graphs must include a description of the bars and the error bars (s.d., s.e.m.).

8) At EMBO Press we ask authors to provide source data for the main manuscript figures. You will receive a separate email with instructions for providing source data with your revised manuscript, including how to upload and organize the files.

9) Our journal encourages inclusion of *data citations in the reference list* to directly cite datasets that were re-used and obtained from public databases. Data citations in the article text are distinct from normal bibliographical citations and should directly link to the database records from which the data can be accessed. In the main text, data citations are formatted as follows: "Data ref: Smith et al, 2001" or "Data ref: NCBI Sequence Read Archive PRJNA342805, 2017". In the Reference list, data citations must be labeled with "[DATASET]". A data reference must provide the database name, accession number/identifiers and a resolvable link to the landing page from which the data can be accessed at the end of the reference.

10) We replaced Supplementary Information with Expanded View (EV) Figures and Tables that are collapsible/expandable

online. A maximum of 5 EV Figures can be typeset. EV Figures should be cited as 'Figure EV1, Figure EV2" etc... in the text and their respective legends should be included in the main text after the legends of regular figures.

- For the figures that you do NOT wish to display as Expanded View figures, they should be bundled together with their legends in a single PDF file called *Appendix*, which should start with a short Table of Content. Appendix figures should be referred to in the main text as: "Appendix Figure S1, Appendix Figure S2" etc.

- Additional Tables/Datasets should be labeled and referred to as Table EV1, Dataset EV1, etc. Legends have to be provided in a separate tab in case of .xls files. Alternatively, the legend can be supplied as a separate text file (README) and zipped together with the Table/Dataset file.

11) The paper explained: EMBO Molecular Medicine articles are accompanied by a summary of the articles to emphasize the major findings in the paper and their medical implications for the non-specialist reader. Please provide a draft summary of your article highlighting
- the medical issue you are addressing,
- the results obtained and
- their clinical impact.

This may be edited to ensure that readers understand the significance and context of the research. Please refer to any of our published articles for an example.

12) Author contributions: You will be asked to provide CRediT (Contributor Role Taxonomy) terms in the submission system. These replace a narrative author contribution section in the manuscript.

13) A "Disclosure and Competing Interests Statement" should be provided in the main text.

14) Every published paper includes a 'Synopsis' to further enhance discoverability. Synopses are displayed on the journal webpage and are freely accessible to all readers. They include a short stand first (maximum of 300 characters, including space) as well as 2-5 one-sentences bullet points that summarizes the paper. Please write the bullet points to summarize the key NEW findings. They should be designed to be complementary to the abstract - i.e. not repeat the same text. We encourage inclusion of key acronyms and quantitative information (maximum of 30 words / bullet point). Please use the passive voice. Please attach these in a separate file or send them by email, we will incorporate them accordingly.

Please also suggest a striking image or visual abstract to illustrate your article as a PNG file 550 px wide x 300 px high.

15) Include a Reagents and Tools Table as part of the Methods section, which can be downloaded from our author guidelines.

16) As part of the EMBO Publications transparent editorial process initiative (see our Editorial at http://embomolmed.embopress.org/content/2/9/329), EMBO Molecular Medicine will publish online a Review Process File (RPF) to accompany accepted manuscripts.
In the event of acceptance, this file will be published in conjunction with your paper and will include the anonymous referee reports, your point-by-point response and all pertinent correspondence relating to the manuscript. Let us know whether you agree with the publication of the RPF and as here, if you want to remove or not any figures from it prior to publication.
Please note that the Authors checklist will be published at the end of the RPF.

I look forward to seeing a revised form of your manuscript as soon as possible.

With kind regards,

Lise Roth

Lise Roth, Ph.D
Senior Editor
EMBO Molecular Medicine

***** Reviewer's comments *****

Referee #1 (Comments on Novelty/Model System for Author):

The authors have just made some comments on my remarks on the translational value of their work. In the present form, the manuscript has a very low translational impact.

Referee #1 (Remarks for Author):

I feel like my previous comments to the author were addressed only by slightly adapting the text or claiming inversion in figures. I remain convinced that the translational potential of the approach is low without a better characterization of the long-term impact of AAV expression in tumors.

Referee #2 (Comments on Novelty/Model System for Author):

The manuscript is technically sound and uses appropriate experimental models to support the main conclusions. The AAV-STITCH platform is innovative and provides a potentially versatile strategy for cell-specific AAV targeting. The work addresses an important limitation of current AAV-based gene therapy approaches and will be of broad interest to the gene therapy and cancer research communities.

Referee #2 (Remarks for Author):

The authors have satisfactorily addressed the major points raised in my initial review. The additional biodistribution and safety analyses strengthen the manuscript. The revised Discussion appropriately acknowledges the current limitations and translational challenges. While some mechanistic questions remain, they do not affect the main conclusions of the study. I therefore support publication of this manuscript.

Referee #1 (Comments on Novelty/Model System for Author):
The authors have just made some comments on my remarks on the translational value of their work. In the present form, the manuscript has a very low translational impact.

Answer:
We respectfully disagree with this reviewer's assessment and have expanded our response in the discussion at page. 13 of the manuscript to more clearly explain the rationale, potential applications, and therapeutic value of our approach. In particular, we have highlighted both its translational potential and its compatibility with clinical development in the discussion together with the remaining challenges for its exploitation.

Referee #1 (Remarks for Author):
I feel like my previous comments to the author were addressed only by slightly adapting the text or claiming inversion in figures. I remain convinced that the translational potential of the approach is low without a better characterization of the long-term impact of AAV expression in tumors.

Answer:
We agree with the reviewer that the use of a non-integrating AAV vector in a suicide gene strategy leads to progressive dilution of the vector genomes as tumor cells proliferate, ultimately limiting long-term transgene persistence. Nevertheless, our data demonstrate that a single administration of AAV-STITCHαGD2 is sufficient to achieve a robust therapeutic effect. Specifically, two weeks after treatment, tumor burden was reduced by approximately 60%, and this early antitumor activity translated into a significant survival benefit, with a 30% increase in median overall survival. These findings indicate that, despite the transient nature of AAV-mediated transgene expression in proliferating tumor cells, the initial level of tumor targeting and cytotoxic activity is sufficient to substantially delay tumor progression and confer a meaningful survival advantage.
Moreover, because its mechanism of action is independent of tumor cell proliferation, this approach is complementary to standard anticancer therapies. It therefore represents a readily combinable therapeutic strategy with the potential to further enhance treatment efficacy against this highly aggressive and difficult-to-treat metastatic tumor.

Referee #2 (Comments on Novelty/Model System for Author):
The manuscript is technically sound and uses appropriate experimental models to support the main conclusions. The AAV-STITCH platform is innovative and provides a potentially versatile strategy for cell-specific AAV targeting. The work addresses an important limitation of current AAV-based gene therapy approaches and will be of broad interest to the gene therapy and cancer research communities.

Answer:
We thank the reviewer for the positive assessment of our work and for recognizing both the conceptual relevance and the therapeutic potential of this approach.

Referee #2 (Remarks for Author):
The authors have satisfactorily addressed the major points raised in my initial review. The additional biodistribution and safety analyses strengthen the manuscript. The revised Discussion appropriately acknowledges the current limitations and translational challenges. While some mechanistic questions remain, they do not affect the main conclusions of the study. I therefore support publication of this manuscript.

Answer:

We are sincerely grateful to the reviewer for the positive assessment of our manuscript and for recommending its publication.

29th Jul 2026

Dear Dr. Broccoli,

Thank you for submitting your revised files. I will be pleased to accept your manuscript once the following editorial matters are addressed:

1/ Manuscript text
- We appreciate that you have expanded the discussion of the potential translational limitations in response to the previous referees' comments. Please also review the concluding paragraph to ensure that the translational implications are stated in appropriately cautious terms.
- Please note that the changes in the submitted revised manuscript were not highlighted. For your next submission, please use track changes to indicate all modifications.
- I suggest changing the title to: Targeted AAV gene therapy for neuroblastoma via direct capsid-antibody coupling, let me know if you agree.
- Remove the One Sentence Summary.
- Please update the order and the headings of the manuscript sections to: Abstract / The Paper Explained / Introduction / Results / Discussion / Methods / Data Availability / Acknowledgements / Disclosure and Competing Interests Statement / References / Figure Legends / Expanded View Figure Legends
- Please remove data not shown (p.8). As per journal policy, all data discussed in the manuscript must be shown in the main or expanded view figures.
- Methods
o Mice: please provide the reference number for ethics approval. Indicate the origine, age, and sex of the mice, and detail the housing and husbandry conditions.
o Cells: please indicate if the cells were authenticated and tested for mycoplasma contamination.
o Patient-derived cells: State details of authority granting ethics approval (IRB or equivalent committee(s), provide reference number for approval. Please also include a statement confirming that informed consent was obtained from all subjects and that the experiments conformed to the principles set out in the WMA Declaration of Helsinki and the Department of Health and Human Services Belmont Report. If the Childhood Cancer Repository explicitly states that all distributed samples are de-identified and covered by its own ethics approval and consent procedures, please simply cite those approvals.
o Statistics: please provide information on exclusion/inclusion criteria.
o BIORENDER: remove from Acknowledgments and add to the Methods as a dedicated section following this example:
Graphics:
(some of the... OR Figure #... OR synopsis) Graphics were created with BioRender.com.

2/ Figures
- You are welcome to change your Appendix figures to Expanded View figures for increased accessibility.
- Please ensure all figures/figures panels are correctly referenced in the manuscript text. Currently, there are callouts for Fig S1H, S1I, EV58A-C and EV58C in the manuscript, please correct.
- Please address the queries from our data editors in the figure legends:
1. Please note that the exact p values are not provided in the legends of figures 1E, G, H; 2D, 4C, D; 5B, 6E, 7C, EV1 I, EV2 B, EV3 A-C; EV5 A, C; S1 B; S3B, C, S4 B
2. Please note that the box plots need to be defined in terms of minima, maxima, centre, bounds of box and whiskers, and percentile in the legends of figures EV1 G
3. Please note that information related to n is missing in the legends of figures 4D, 5B, EV3 A-C
4. Please note that the measure of center for the error bars needs to be defined in the legends of figures 7C, EV1 H, I; EV2 B, EV3 A-C; EV5 A-D. S1B, S3B, C; S4B
5. Please note that the scale bar is missing for figure 3B
6. Please note that the dashed lines are not defined in the legend of figure EV4 B. This needs to be rectified.
7. Please note that the black arrows are not defined in the legend of figure 6D. This needs to be rectified.

3/ Author Checklist:
Please fill in the following:
- Experimental study design and statistics; inclusion/exclusion criteria
- Ethics; authority granting ethics approval, informed consent and Helsinki Declaration (if applicable)
- Data availability; deposition of primary datasets

4/ Synopsis:
- I introduced minor changes in your text, please let me know if you agree or amend as you see fit:
A modular AAV platform enables antibody-directed viral tropism through covalent capsid engineering. Selective systemic targeting of neuroblastoma was achieved, allowing efficient suicide-gene delivery with potent antitumor activity and minimal off-target transduction.

- A modular AAV-STITCH platform enables covalent coupling of scFvs to an infectivity-deficient AAV9 capsid through SpyTag/SpyCatcher chemistry.
- Systemic administration selectively transduced $GD2^{+}$ neuroblastoma cells while largely abolishing transduction of healthy tissues.
- AAV-STITCHαGD2-TK produced potent antitumor activity, reducing metastatic tumor burden and prolonging survival after a single treatment.
- Combining AAV-STITCHαGD2-TK with TOTEM chemotherapy resulted in near-complete suppression of tumor growth, supporting complementary therapeutic mechanisms.
- The platform was successfully retargeted to $HER2^{+}$ cancer cells, demonstrating its adaptability for precision gene delivery.

- The visual abstract should be uploaded as a png/tiff/jpeg file, with the dimensions 550 pixels wide x 300 pixels high. Please also ensure that the text remains legible.

5/ As part of the EMBO Publications transparent editorial process initiative (see our Editorial at http://embomolmed.embopress.org/content/2/9/329), EMBO Molecular Medicine will publish online a Review Process File (RPF) to accompany accepted manuscripts.
This file will be published in conjunction with your paper and will include the anonymous referee reports, your point-by-point response and all pertinent correspondence relating to the manuscript. Let us know whether you agree with the publication of the RPF.
Please note that the Authors checklist will be published at the end of the RPF.

With kind regards,

Lise Roth

Lise Roth, Ph.D
Senior Editor
EMBO Molecular Medicine

The authors addressed the remaining editorial changes.

28th Aug 2026

Dear Dr. Broccoli, Dear Vania,

Thank you for submitting your revised files. I am pleased to inform you that your manuscript has been accepted for publication!

Please note that we still require Figure 3 with a scale bar (defined in the legend but absent from the figure) before we can send your manuscript to our publisher for inclusion in the next available issue of EMBO Molecular Medicine. Please provide the revised figure at your earliest convenience.

Your manuscript will be processed for publication by EMBO Press. It will be copy edited and you will receive page proofs prior to publication. Please note that you will be contacted by Springer Nature Author Services to complete licensing and payment information.

You may qualify for financial assistance for your publication charges - either via a Springer Nature fully open access agreement or an EMBO initiative. Check your eligibility: https://link.springer.com/journal/44321/how-to-publish-with-us

Should you be planning a Press Release on your article, please notify the production team via this form in order to coordinate publication and release dates: https://forms.cloud.microsoft/e/eH4A8JVP6g

If you have any questions, please do not hesitate to contact the Editorial Office.

Thank you for your contribution to EMBO Molecular Medicine.

With kind regards,

Lise

Lise Roth, Ph.D
Senior Editor
EMBO Molecular Medicine

>>> Please note that it is EMBO Molecular Medicine policy for the transcript of the editorial process (containing referee reports and your response letter) to be published as an online supplement to each paper. If you do NOT want this, you will need to inform the Editorial Office via email immediately. More information is available here: https://link.springer.com/partners/embo-press/editorial-policies#Peer%20review

EMBO Press Author Checklist

Corresponding Author Name: Vania Broccoli	
Journal Submitted to: EMBO Molecular Medicine	
Manuscript Number: EMM-2025-23115	

USEFUL LINKS FOR COMPLETING THIS FORM
The EMBO Journal - Author Guidelines
EMBO Reports - Author Guidelines
Molecular Systems Biology - Author Guidelines
EMBO Molecular Medicine - Author Guidelines

Reporting Checklist for Life Science Articles (updated January
This checklist is adapted from Materials Design Analysis Reporting (MDAR) Checklist for Authors. MDAR establishes a minimum set of requirements in transparent reporting in the life sciences (see Statement of Task: 10.31222/osf.io/9sm4x). Please follow the journal's guidelines in preparing your
Please note that a copy of this checklist will be published alongside your article.

Abridged guidelines for figures
1. Data
The data shown in figures should satisfy the following conditions:
- the data were obtained and processed according to the field's best practice and are presented to reflect the results of the experiments in an accurate and unbiased manner.
- ideally, figure panels should include only measurements that are directly comparable to each other and obtained with the same assay.
- plots include clearly labeled error bars for independent experiments and sample sizes. Unless justified, error bars should not be shown for technical
- if n<5, the individual data points from each experiment should be plotted. Any statistical test employed should be justified.
- Source Data should be included to report the data underlying figures according to the guidelines set out in the authorship guidelines on Data

2. Captions
Each figure caption should contain the following information, for each panel where they are relevant:
- a specification of the experimental system investigated (eg cell line, species name).
- the assay(s) and method(s) used to carry out the reported observations and measurements.
- an explicit mention of the biological and chemical entity(ies) that are being measured.
- an explicit mention of the biological and chemical entity(ies) that are altered/varied/perturbed in a controlled manner.
- the exact sample size (n) for each experimental group/condition, given as a number, not a range;
- a description of the sample collection allowing the reader to understand whether the samples represent technical or biological replicates (including how many animals, litters, cultures, etc.).
- a statement of how many times the experiment shown was independently replicated in the laboratory.
- definitions of statistical methods and measures:
  - common tests, such as t-test (please specify whether paired vs. unpaired), simple χ2 tests, Wilcoxon and Mann-Whitney tests, can be unambiguously identified by name only, but more complex techniques should be described in the methods section;
  - are tests one-sided or two-sided?
  - are there adjustments for multiple comparisons?
  - exact statistical test results, e.g., P values = x but not P values < x;
  - definition of 'center values' as median or average;
  - definition of error bars as s.d. or s.e.m.

Please complete ALL of the questions below.
Select "Not Applicable" only when the requested information is not relevant for your study.

Materials

**Newly Created Materials**	**Information included in the manuscript?**	**In which section is the information available?** (Reagents and Tools Table, Materials and Methods, Figures, Data Availability Section)
New materials and reagents need to be available; do any restrictions apply?	Yes	

**Antibodies**	**Information included in the manuscript?**	**In which section is the information available?** (Reagents and Tools Table, Materials and Methods, Figures, Data Availability Section)
For **antibodies** provide the following information:<br>- Commercial antibodies: RRID (if possible) or supplier name, catalogue number and or/clone number<br>- Non-commercial: RRID or citation	Yes	Reagents and Tools Table

**DNA and RNA sequences**	**Information included in the manuscript?**	**In which section is the information available?** (Reagents and Tools Table, Materials and Methods, Figures, Data Availability Section)
**Short novel DNA or RNA including primers, probes**: provide the sequences.	Yes	Reagents and Tools Table

**Cell materials**	**Information included in the manuscript?**	**In which section is the information available?** (Reagents and Tools Table, Materials and Methods, Figures, Data Availability Section)
**Cell lines:** Provide species information, strain. Provide accession number in repository **OR** supplier name, catalog number, clone number, and/**OR** RRID.	Yes	Methods
**Primary cultures:** Provide species, strain, sex of origin, genetic modification status.	Yes	Methods
Report if the cell lines were recently **authenticated** (e.g., by STR profiling) and tested for mycoplasma contamination.	Yes	Methods

**Experimental animals**	**Information included in the manuscript?**	**In which section is the information available?** (Reagents and Tools Table, Materials and Methods, Figures, Data Availability Section)
**Laboratory animals or Model organisms:** Provide species, strain, sex, age, genetic modification status. Provide accession number in repository **OR** supplier name, catalog number, clone number, **OR** RRID.	Yes	Methods
**Animal observed in or captured from the field:** Provide species, sex, and age where possible.	Not Applicable	
Please detail **housing and husbandry conditions**.	Yes	Methods

**Plants and microbes**	**Information included in the manuscript?**	**In which section is the information available?** (Reagents and Tools Table, Materials and Methods, Figures, Data Availability Section)
**Plants:** provide species and strain, ecotype and cultivar where relevant, unique accession number if available, and source (including location for collected wild specimens).	Not Applicable	
**Microbes:** provide species and strain, unique accession number if available, and source.	Not Applicable	

**Human research participants**	**Information included in the manuscript?**	**In which section is the information available?** (Reagents and Tools Table, Materials and Methods, Figures, Data Availability Section)
If collected and within the bounds of privacy constraints report on age, sex and gender or ethnicity for all study participants.	Not Applicable	

**Core facilities**	**Information included in the manuscript?**	**In which section is the information available?** (Reagents and Tools Table, Materials and Methods, Figures, Data Availability Section)
If your work benefited from core facilities, was their service mentioned in the acknowledgments section?	Yes	Acknowledgments

Design

Study protocol	Information included in the manuscript?	In which section is the information available? (Reagents and Tools Table, Materials and Methods, Figures, Data Availability Section)
If study protocol has been **pre-registered, provide DOI in the manuscript**. For clinical trials, provide the trial registration number **OR** cite DOI.	Not Applicable	
Report the **clinical trial registration number** (at ClinicalTrials.gov or equivalent), where applicable.	Not Applicable	

Laboratory protocol	Information included in the manuscript?	In which section is the information available? (Reagents and Tools Table, Materials and Methods, Figures, Data Availability Section)
Provide DOI OR other citation details if **external detailed step-by-step protocols** are available.	Not Applicable	

Experimental study design and statistics	Information included in the manuscript?	In which section is the information available? (Reagents and Tools Table, Materials and Methods, Figures, Data Availability Section)
Include a statement about **sample size** estimate even if no statistical methods were used.	Yes	Methods
Were any steps taken to minimize the effects of subjective bias when allocating animals/samples to treatment (e.g. **randomization procedure**)? If yes, have they been described?	Yes	Methods
Include a statement about **blinding** even if no blinding was done.	Yes	Methods
Describe **inclusion/exclusion criteria** if samples or animals were excluded from the analysis. Were the criteria pre-established? If sample or data points were omitted from analysis, report if this was due to attrition or intentional exclusion and provide justification.	Yes	Methods
For every figure, are **statistical tests** justified as appropriate? Do the data meet the assumptions of the tests (e.g., normal distribution)? Describe any methods used to assess it. Is there an estimate of variation within each group of data? Is the variance similar between the groups that are being statistically compared?	Yes	Methods and Figure Legends

Sample definition and in-laboratory replication	Information included in the manuscript?	In which section is the information available? (Reagents and Tools Table, Materials and Methods, Figures, Data Availability Section)
In the figure legends: state number of times the experiment was **replicated** in laboratory.	Yes	Figure Legends
In the figure legends: define whether data describe **technical or biological replicates**.	Yes	Figure Legends

Ethics

Ethics	Information included in the manuscript?	In which section is the information available? (Reagents and Tools Table, Materials and Methods, Figures, Data Availability Section)
Studies involving **human participants**: State details of **authority granting ethics approval** (IRB or equivalent committee(s), provide reference number for approval.	Not Applicable	
Studies involving **human participants**: Include a statement confirming that **informed consent** was obtained from all subjects and that the experiments conformed to the principles set out in the WMA Declaration of Helsinki and the Department of Health and Human Services Belmont Report.	Yes	methods
Studies involving **human participants: ** For publication of **patient photos**, include a statement confirming that consent to publish was obtained.	Not Applicable	
Studies involving experimental **animals**: State details of **authority granting ethics approval** (IRB or equivalent committee(s), provide reference number for approval. Include a statement of compliance with ethical regulations.	Yes	Methods
Studies involving **specimen and field samples:** State if relevant **permits** obtained, provide details of authority approving study; if none were required, explain why.	Not Applicable	

Dual Use Research of Concern (DURC)	Information included in the manuscript?	In which section is the information available? (Reagents and Tools Table, Materials and Methods, Figures, Data Availability Section)
Could your study fall under dual use research restrictions? Please check biosecurity documents and list of **select agents and toxins** (CDC): https://www.selectagents.gov/sat/list.htm	Not Applicable	
If you used a select agent, is the security level of the lab appropriate and reported in the manuscript?	Not Applicable	
If a study is subject to dual use research of concern regulations, is the name of the **authority granting approval and reference number** for the regulatory approval provided in the manuscript?	Not Applicable	

Reporting

The MDAR framework recommends adoption of discipline-specific guidelines, established and endorsed through community initiatives. Journals have their own policy about requiring specific guidelines and recommendations to complement MDAR.

Adherence to community standards	Information included in the manuscript?	In which section is the information available? (Reagents and Tools Table, Materials and Methods, Figures, Data Availability Section)
State if relevant guidelines or checklists (e.g., **ICMJE, MIBBI, ARRIVE, PRISMA**) have been followed or provided.	Not Applicable	
For **tumor marker prognostic studies,** we recommend that you follow the **REMARK** reporting guidelines (see link list at top right). See author guidelines, under 'Reporting Guidelines'. Please confirm you have followed these guidelines.	Not Applicable	
For **phase II and III randomized controlled trials**, please refer to the **CONSORT** flow diagram (see link list at top right) and submit the CONSORT checklist (see link list at top right) with your submission. See author guidelines, under 'Reporting Guidelines'. Please confirm you have submitted this list.	Not Applicable	

Data Availability

Data availability	Information included in the manuscript?	In which section is the information available? (Reagents and Tools Table, Materials and Methods, Figures, Data Availability Section)
Have **primary datasets** been deposited according to the journal's guidelines (see 'Data Deposition' section) and the respective accession numbers provided in the Data Availability Section?	Yes	Data availability
Were **human clinical and genomic datasets** deposited in a public access-controlled repository in accordance to ethical obligations to the patients and to the applicable consent agreement?	Not Applicable	
Are **computational models** that are central and integral to a study available without restrictions in a machine-readable form? Were the relevant accession numbers or links provided?	Not Applicable	
If publicly available data were reused, provide the respective **data citations in the reference list**.	Not Applicable	
