# Supplementary material for: Targeted AAV gene therapy for neuroblastoma via direct capsid-antibody coupling
Source: EMBO Mol Med. 2026 Sep 12;18(10):4117–52. doi: 10.1038/s44321-026-00520-7 (PMC13658805; doi:10.1038/s44321-026-00520-7)
Supplement: Supplementary file 2 [file 44321_2026_520_MOESM8_ESM.pdf]

Expanded View Figures

Figure EV1. Optimization of AAV-STITCH production comparing post-production and co-expression protocols.

(**A**) Representative Coomassie staining and western blot with anti-V5 antibody showing the purification of the anti-GD2 scFv protein (αGD2-SC) via His-tag affinity isolation. (**B**) Western blot with anti-V5 of purified αGD2-SC protein treated with deglycosylation enzymes for 1 and 16 h, demonstrating the effect of enzymatic deglycosylation. (**C**) Representative Western blot with anti-AAV showing the post-production coupling protocol, in which purified αGD2-SC was incubated with AAV9m-ST at different ratios (1:200, 1:400, 1:600, and 1:800). (**D**) Representative anti-V5 Western blot showing the detection of αGD2-SC fused to AAV9m-ST after post-production coupling protocol. (**E**) Western blot anti-V5 showing the detection of αGD2-SC fused to AAV9m-ST after co-expression coupling protocol. (**F**) Western blot with anti-V5 antibody showing the intracellular αGD2-SC band. (**G**) Box plot showing the viral genome titers (vg/plate), assessed by RT-qPCR, from multiple independent productions of AAV9, AAV9m, AAV9m-ST, and 1:3 and 1:7 mosaic preparations. AAV9: $n = 12$; minimum $= 2.40 \times 10^{10}$; Q1 (25%) $= 4.12 \times 10^{10}$; Median $= 1.11 \times 10^{11}$; Q3 (75%) $= 1.64 \times 10^{11}$; maximum $= 3.91 \times 10^{12}$. AAV9m: $n = 4$; minimum $= 2.89 \times 10^{10}$; Q1 (25%) $= 3.23 \times 10^{10}$; Median $= 4.71 \times 10^{10}$; Q3 (75%) $= 1.03 \times 10^{11}$; maximum $= 2.30 \times 10^{11}$. AAV9m-ST: $n = 4$; minimum $= 5.69 \times 10^{10}$; Q1 (25%) $= 7.33 \times 10^{10}$; Median $= 1.24 \times 10^{11}$; Q3 (75%) $= 2.97 \times 10^{11}$; maximum $= 6.82 \times 10^{11}$. AAV9m-STITCH: $n = 6$; minimum $= 1.89 \times 10^{10}$; Q1 (25%) $= 2.60 \times 10^{10}$; Median $= 5.89 \times 10^{10}$; Q3 (75%) $= 1.97 \times 10^{11}$; maximum $= 3.75 \times 10^{11}$. AAV9-STITCH 1:3: $n = 18$; minimum $= 2.36 \times 10^{10}$; Q1 (25%) $= 4.08 \times 10^{10}$; Median $= 9.60 \times 10^{10}$; Q3 (75%) $= 2.39 \times 10^{11}$; maximum $= 4.07 \times 10^{12}$. AAV9-STITCH 1:7: $n = 7$; minimum $= 1.47 \times 10^{10}$; Q1 (25%) $= 2.69 \times 10^{10}$; Median $= 3.34 \times 10^{10}$; Q3 (75%) $= 6.08 \times 10^{10}$; maximum $= 2.30 \times 10^{11}$. (**H**) Left: Western blot anti-AAV showing the detection of αGD2-SC fused to AAV9m-ST after co-expression coupling protocol. Right: Densitometric quantification of the proportion of unconjugated VP3 (gray) and VP3 conjugated with αGD2-SC (orange). Data are presented as mean ± SD. (**I**) Left: immunofluorescence using anti-ZsGreen antibody on SH-SY5Y cells transduced with control AAV9m-ST alone or couple with αGD2-SC (co-expression coupling protocol). Scale bar:100um. Right: bar graph showing the quantification of ZsGreen+ cells over total cells (DAPI +) in the different experimental conditions. Data are presented as mean ± SD, with individual data points shown ($n = 3$ independent biological replicates). $^{**}P = 0.0031$ AAV9m-ST vs AAV9m-ST-αGD2-SC; *t* Test.

© The Author(s)

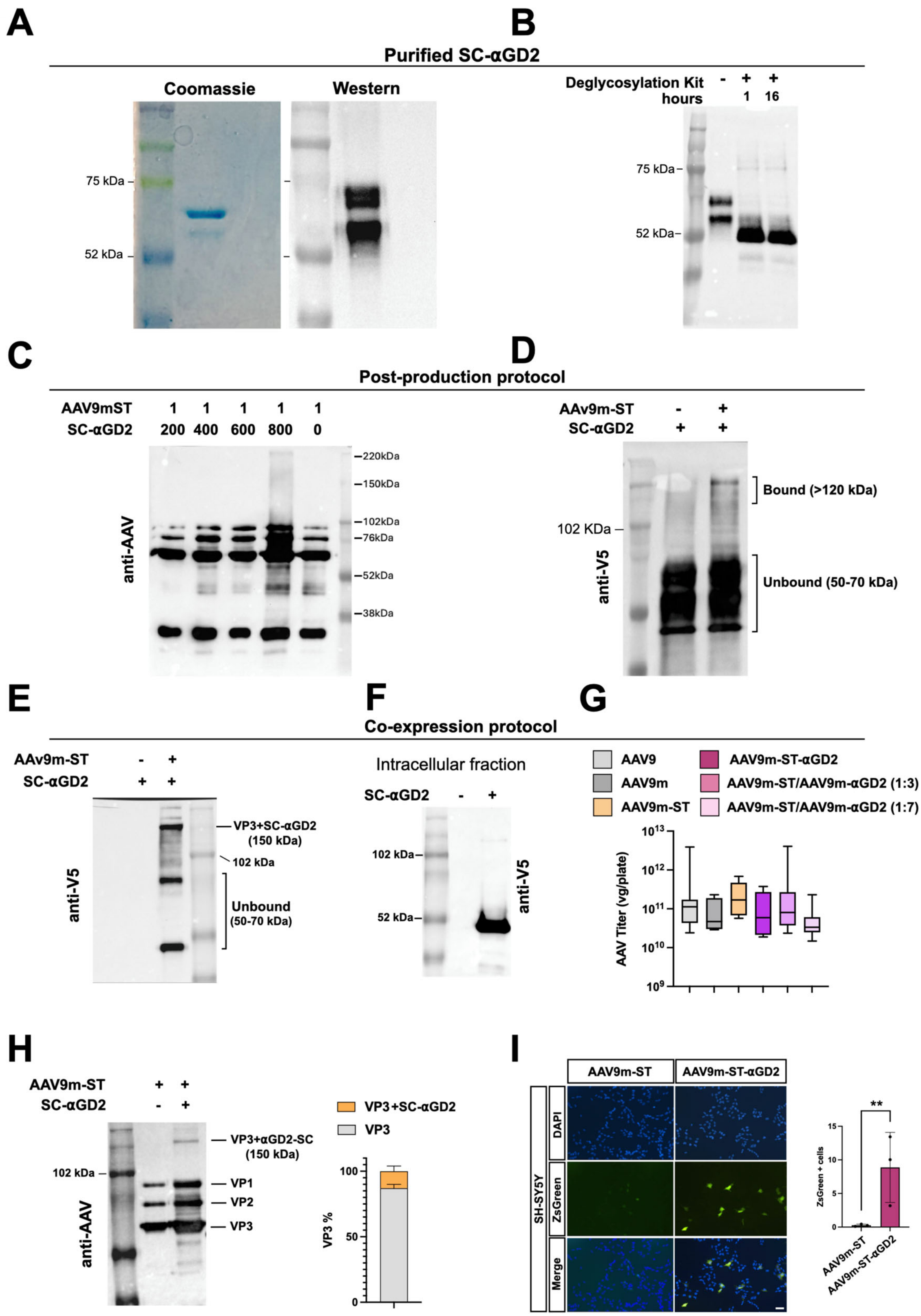
A
B
Purified SC-αGD2
Coomassie
Western
75 kDa
52 kDa
Deglycosylation Kit - + +
hours 1 16
75 kDa
52 kDa
C
D
Post-production protocol
AAV9mST 1 1 1 1 1
SC-αGD2 200 400 600 800 0
anti-AAV
220kDa
150kDa
102kDa
76kDa
52kDa
38kDa
AAv9m-ST - +
SC-αGD2 + +
102 KDa
anti-V5
Bound (>120 kDa)
Unbound (50-70 kDa)
E
F
G
Co-expression protocol
AAv9m-ST - +
SC-αGD2 + +
anti-V5
VP3+SC-αGD2 (150 kDa)
102 kDa
Unbound (50-70 kDa)
Intracellular fraction
SC-αGD2 - +
102 kDa
52 kDa
anti-V5
AAV9
AAV9m
AAV9m-ST
AAV9m-ST-αGD2
AAV9m-ST/AAV9m-αGD2 (1:3)
AAV9m-ST/AAV9m-αGD2 (1:7)
AAV Titer (vg/plate)
H
I
AAV9m-ST + +
SC-αGD2 - +
102 kDa
anti-AAV
VP3+αGD2-SC (150 kDa)
VP1
VP2
VP3
VP3+SC-αGD2
VP3
VP3 %
100
50
0
AAV9m-ST
AAV9m-ST-αGD2
SH-SY5Y
DAPI
ZsGreen
Merge
**
ZsGreen + cells
15
10
5
0
AAV9m-ST
AAV9m-ST-αGD2

 © The Author(s)

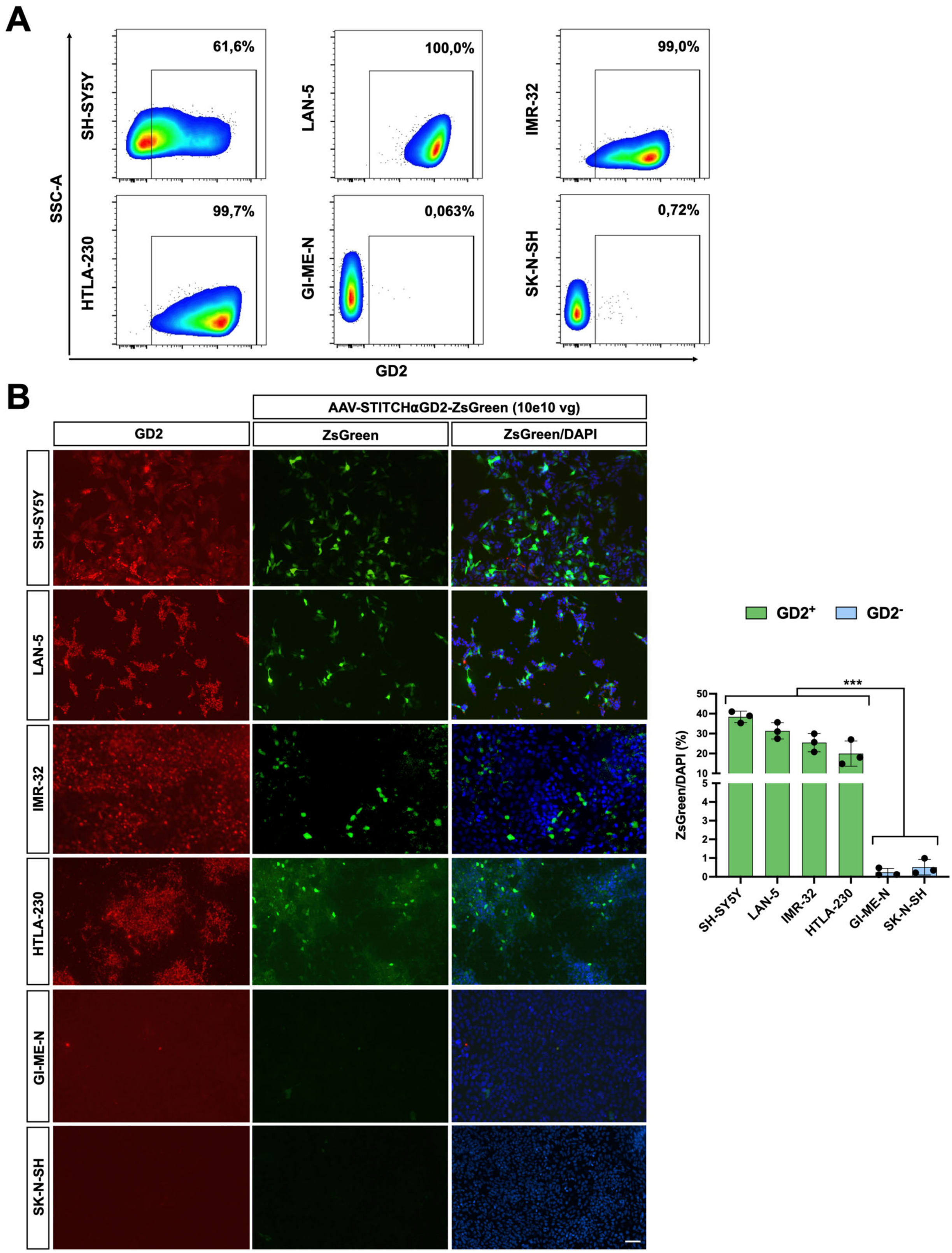
A
SSC-A
GD2
SH-SY5Y
61,6%
LAN-5
100,0%
IMR-32
99,0%
HTLA-230
99,7%
GI-ME-N
0,063%
SK-N-SH
0,72%
B
AAV-STITCHαGD2-ZsGreen (10e10 vg)
GD2
ZsGreen
ZsGreen/DAPI
SH-SY5Y
LAN-5
IMR-32
HTLA-230
GI-ME-N
SK-N-SH
GD2+
GD2-
ZsGreen/DAPI (%)
***
SH-SY5Y
LAN-5
IMR-32
HTLA-230
GI-ME-N
SK-N-SH

◀ **Figure EV2. Specificity and efficiency of AAV-STITCHαGD2 transduction in neuroblastoma cell lines.**

(**A**) Quantification of GD2 expression in the various NB cell lines analyzed in this study by flow cytometry. (**B**) Representative immunofluorescence for anti-GD2 and anti-ZsGreen in GD2+ and GD2− NB cell lines and bar graph showing the quantification of ZsGreen+ cells over total cells in the different NB cell lines transduced with AAV-STITCHαGD2 carrying the ZsGreen transgene. Scale bar: 100 μm. Data are presented as mean ± SD, with individual data points shown ($n = 3$ independent biological replicates). ***$P = 0.0001$ SH-SY5Y vs GI-ME-N. ***$P = 0.0001$ SH-SY5Y vs SK-N-SH. ***$P = 0.0001$ LAN5 vs GI-ME-N. ***$P = 0.0001$ LAN5 vs SK-N-SH. ***$P = 0.0001$ IMR-32 vs GI-ME-N. ***$P = 0.0001$ IMR-32 vs SK-N-SH. ***$P = 0.0004$ HTLA-230 vs GI-ME-N. ***$P = 0.0004$ HTLA-230 vs SK-N-SH. One-way ANOVA with Tukey's multiple comparisons correction.

 © The Author(s)

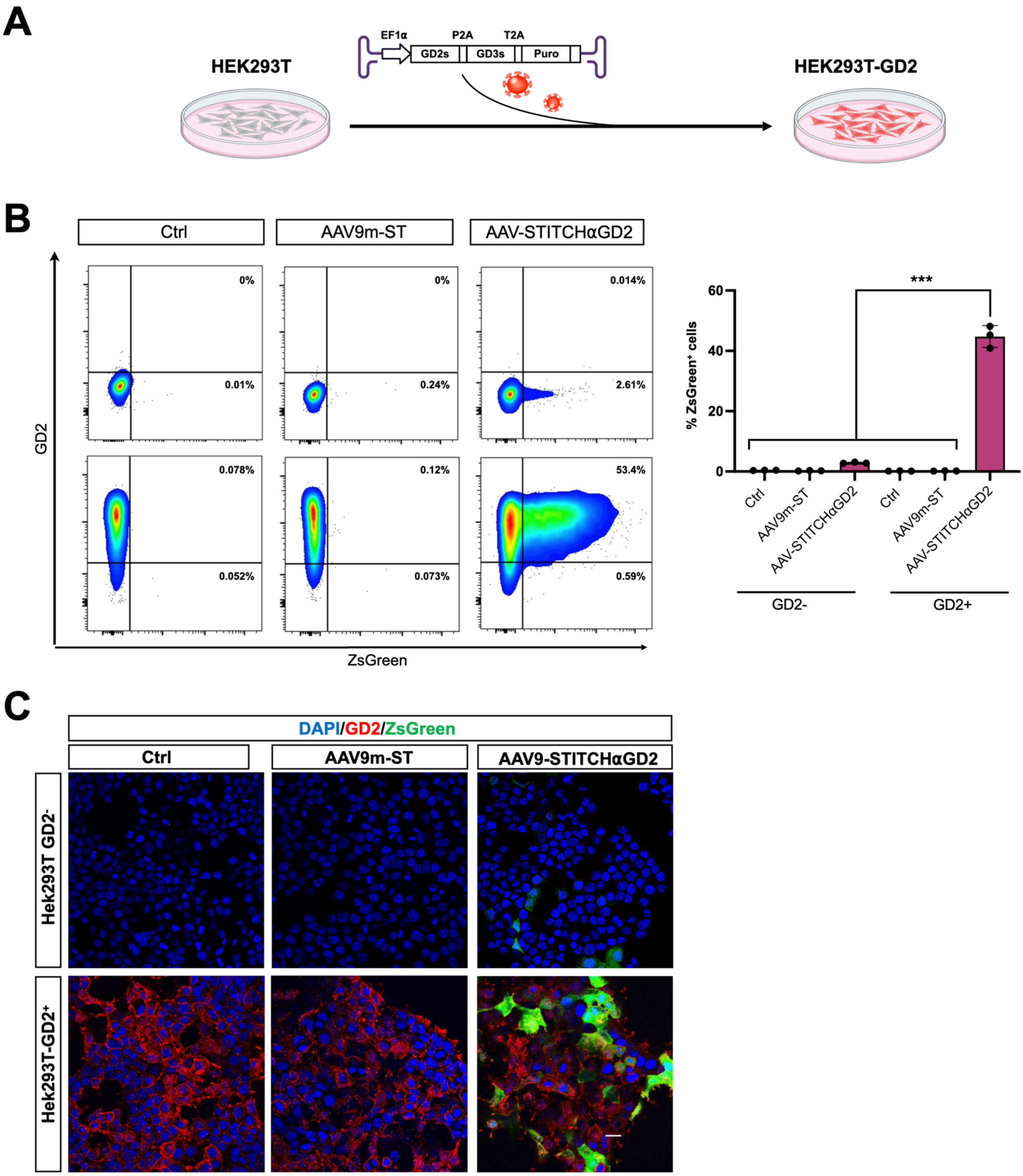
A
HEK293T
EF1α
P2A
T2A
GD2s
GD3s
Puro
HEK293T-GD2
B
Ctrl
AAV9m-ST
AAV-STITCHαGD2
GD2
ZsGreen
0%
0.01%
0.24%
0.014%
2.61%
0.078%
0.052%
0.12%
0.073%
53.4%
0.59%
% ZsGreen⁺ cells
***
GD2-
GD2+
C
DAPI/GD2/ZsGreen
Ctrl
AAV9m-ST
AAV9-STITCHαGD2
Hek293T GD2⁻
Hek293T-GD2⁺

◀ **Figure EV3. Specificity and efficiency of AAV-STITCHαGD2 transduction in HEK293T cells engineered to express GD2.**

(**A**) Illustration of the experimental protocol. HEK293T cells were transduced with a multicistronic lentiviral vector containing the coding sequences (CDS) of GD2 synthase (GD2S), GD3 synthase (GD3S), and a puromycin resistance gene for selection, for generating HEK293-GD2+ cells. (**B**) Left: Flow cytometry analysis of ZsGreen+ (*X* axis) and GD2+ (*Y* axis) cells in HEK293-GD2− and HEK293-GD2+ cells following transduction with AAV-STITCHαGD2 or AAV9m-ST carrying the ZsGreen transgene. Right: Bar graph showing the percentage of ZsGreen+ cells over total cells in HEK293-GD2− and HEK293-GD2+ after transduction. Data are presented as mean ± SD, with individual data points shown ($n = 3$ independent biological replicates). ***$P = 0.0001$ GD2 + AAV-STITCHαGD2 vs GD2- Ctrl; ***$P = 0.0001$ GD2 + AAV-STITCHαGD2 vs GD2- AAV9m-ST; ***$P = 0.0001$ GD2 + AAV-STITCHαGD2 vs GD2- AAV-STITCHαGD2; ***$P = 0.0001$ GD2 + AAV-STITCHαGD2 vs GD2+ Ctrl; ***$P = 0.0001$ GD2 + AAV-STITCHαGD2 vs GD2 + AAV9m-ST. One-way ANOVA with Tukey's multiple comparisons correction. (**C**) Representative immunofluorescence images using anti-ZsGreen and anti-GD2 antibodies in HEK293-GD2− and HEK293-GD2+ cells transduced with AAV-STITCHαGD2 or AAV9m-ST. Scale bar: 100 µm.

© The Author(s)

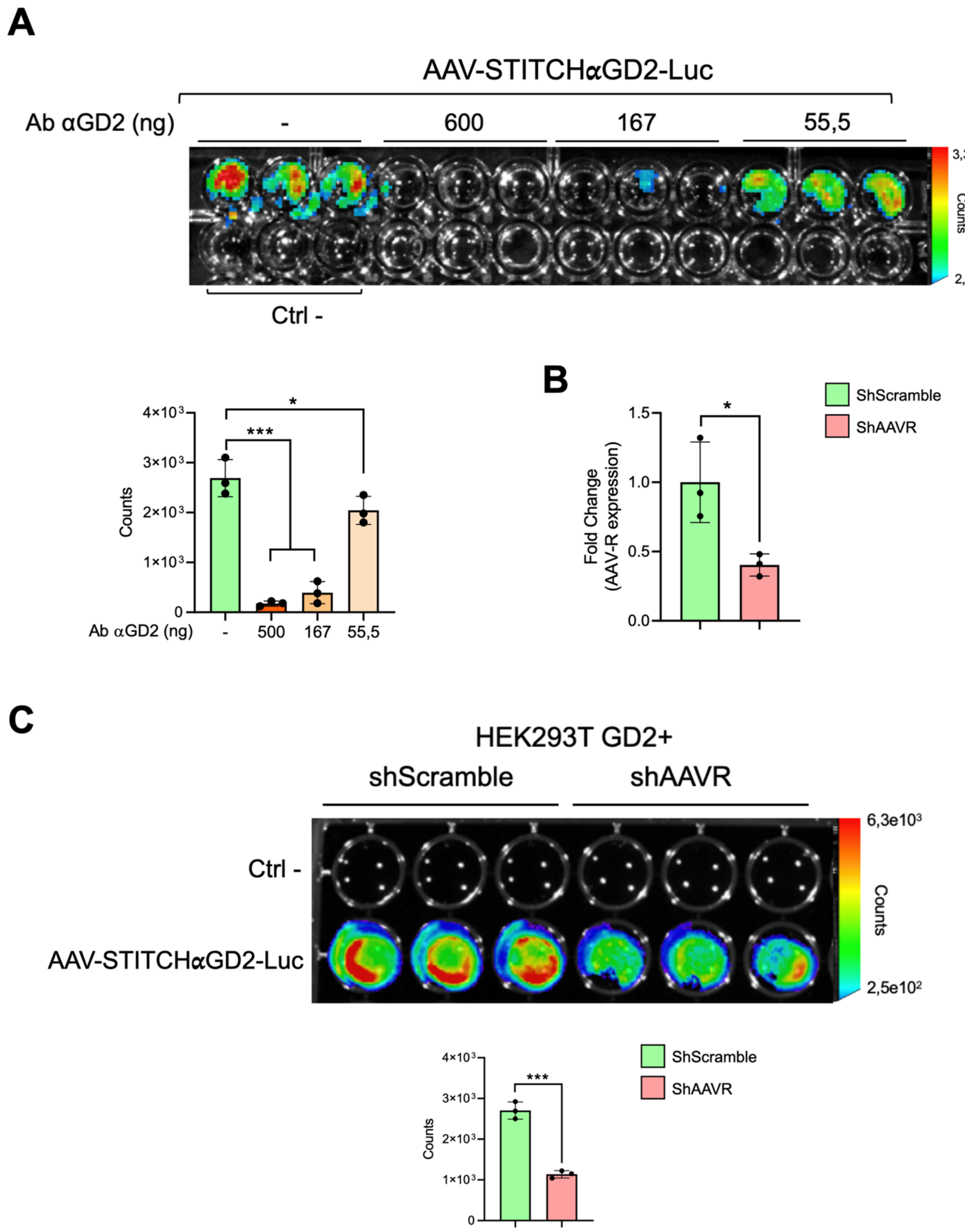

Figure EV4. Anti-GD2 competition assay and AAVR knockdown analysis.

(**A**) Representative IVIS images of HEK293-GD2+ cells transduced with AAV-STITCHαGD2 carrying a CMV-Luc reporter cassette following pre-incubation with anti-GD2 antibody at 5 µg/mL, 1.67 µg/mL, or 0.56 µg/mL. The lower row shows non-transduced control cells. Left, bar graph showing quantification of luminescence signal (counts) in cells pre-incubated or not with anti-GD2 antibody. Data are presented as mean ± SD, with individual data points shown ($n = 3$ independent biological replicates). ****P* = 0.0001 – vs 500; ****P* = 0.0001 – vs 167; **P* = 0.0379 – vs 55.5. One-way ANOVA with Tukey's multiple comparisons correction. (**B**) qRT-PCR analysis showing AAVR silencing in HEK293-GD2+ cells transfected with an shRNA targeting AAVR compared with scramble shRNA control. Gene expression levels were normalized to 18S rRNA. Data are presented as mean ± SD, with individual data points shown ($n = 3$ independent biological replicates). **P* = 0.0265 ShScramble vs ShAAVR; *t* Test. (**C**) Representative IVIS images of HEK293-GD2+ cells transduced with AAV-STITCHαGD2 carrying a CMV-Luc reporter cassette following transfection with either anti-AAVR shRNA or scramble control. The upper row shows non-transduced control cells. Right, bar graph showing quantification of luminescence signal (counts) in cells treated with anti-AAVR shRNA compared with scramble control. Data are presented as mean ± SD, with individual data points shown ($n = 3$ independent biological replicates). ****P* = 0.0003 ShScramble vs ShAAVR; *t* Test.

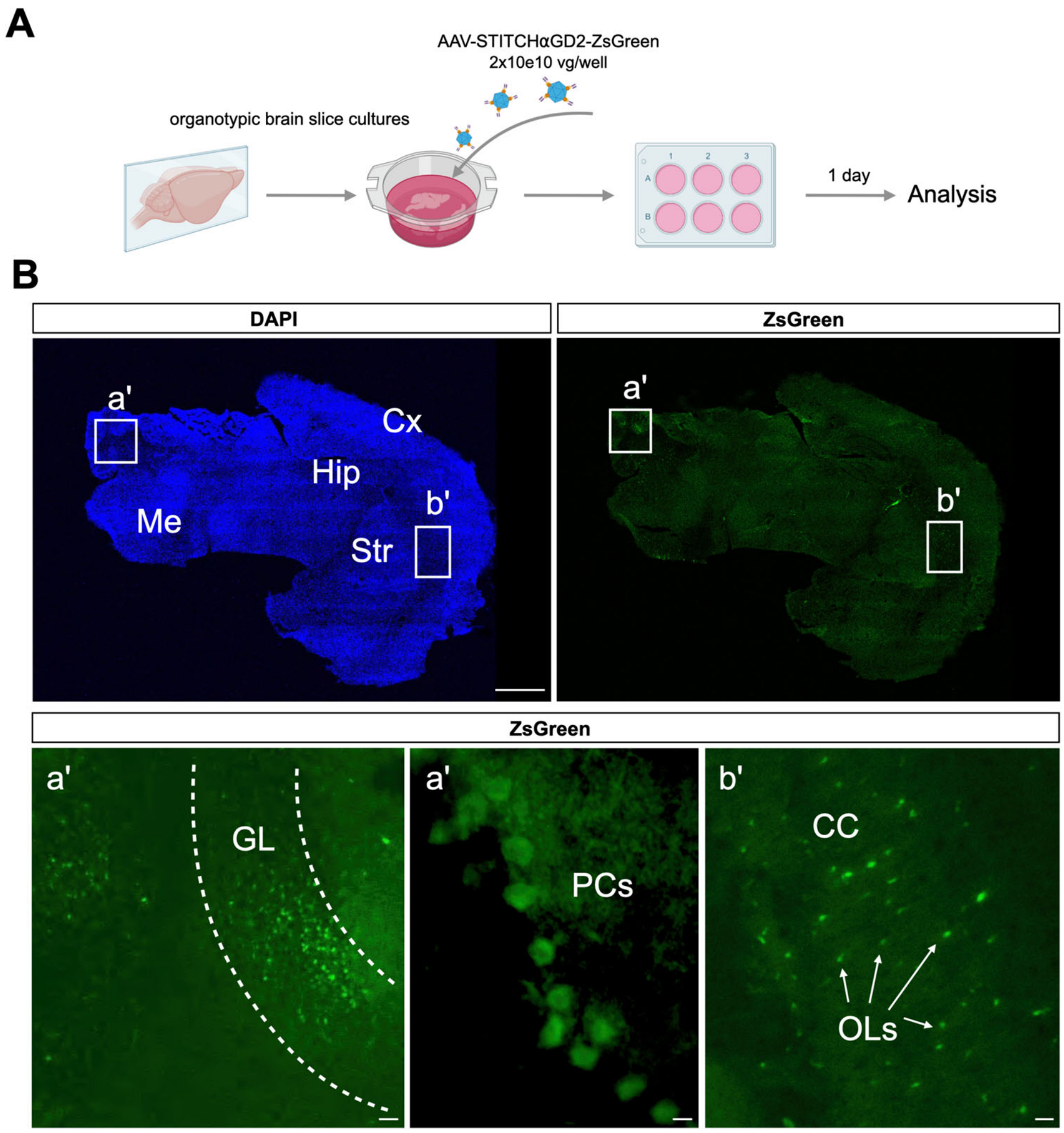

Figure EV5. Transduction profile of AAV-STITCHαGD2 in organotypic murine brain slices.

(**A**) Illustration of the experimental protocol. Murine organotypic brain slices were infected with 2×10^{10} vg/well of AAV-STITCHαGD2 carrying the ZsGreen transgene. The following day, slices were processed and the transduction profile was analyzed by immunofluorescence staining. (**B**) Representative immunofluorescence images of a sagittal brain section transduced with AAV-STITCHαGD2, showing DAPI staining on the left and anti-ZsGreen staining on the right. Scale bar: 1000 µm. (**a**′) Left: Representative higher magnification image of the cerebellum, showing transduced cells in the granular layer (GL). Scale bar: 100 µm. Right: Higher magnification image of cerebellar Purkinje cells (PCs). Scale bar: 100 µm. (**b**′) Representative higher magnification image showing transduced oligodendrocytes (OLs) in the corpus callosum (CC). Scale bar: 100 µm.

 © The Author(s)

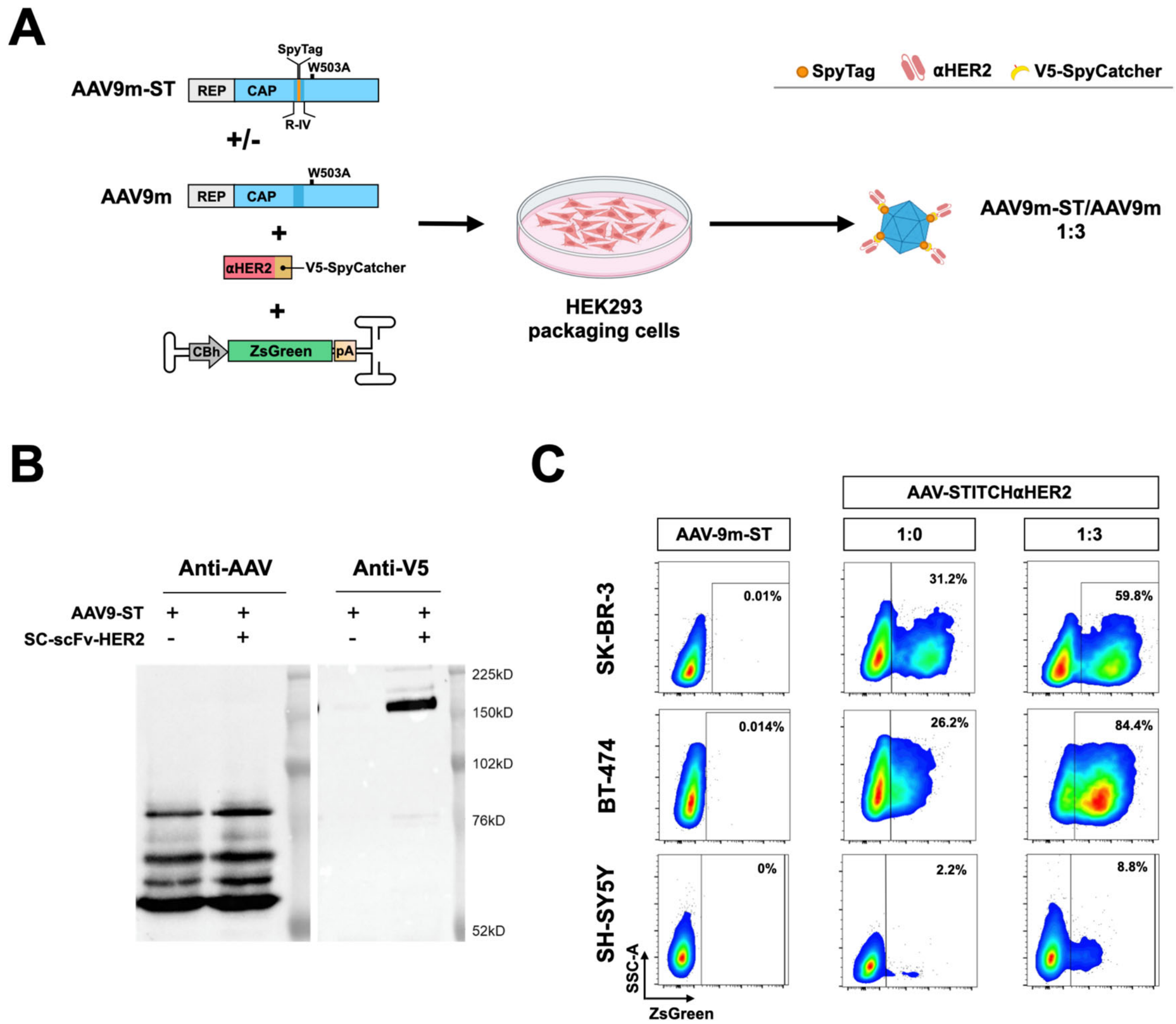

Figure EV6. Production and functional validation of AAV-STITCHαHER2 vectors.

(**A**) Schematic of the experimental protocol used for AAV-STITCHαHER2 capsid production. Plasmids encoding the AAV components were co-expressed together with a construct encoding the SpyCatcher fused to the anti-HER2 scFv tagged with V5 (αHER2-V5-SpyCatcher). In brief, AAV particles were produced in HEK293 cells by packaging the CBh-ZsGreen transgene and by mixing the AAV9m-ST and AAV9m with ratio: 1:3. (**B**) Western blot with anti-AAV and anti-V5 antibodies showing the capsid composition of AAV-STITCHαHER2 comparing to AAV9m-ST mosaic capsid. (**C**) Quantification by flow cytometry of ZsGreen positive SH-SY5Y (HER2-), SK-BR-3 (HER2 +) and BT-474 (HER2 +) cell lines after transduction with AAV-STITCHαHER2 1:0 and 1:3 with respect the untreated control.

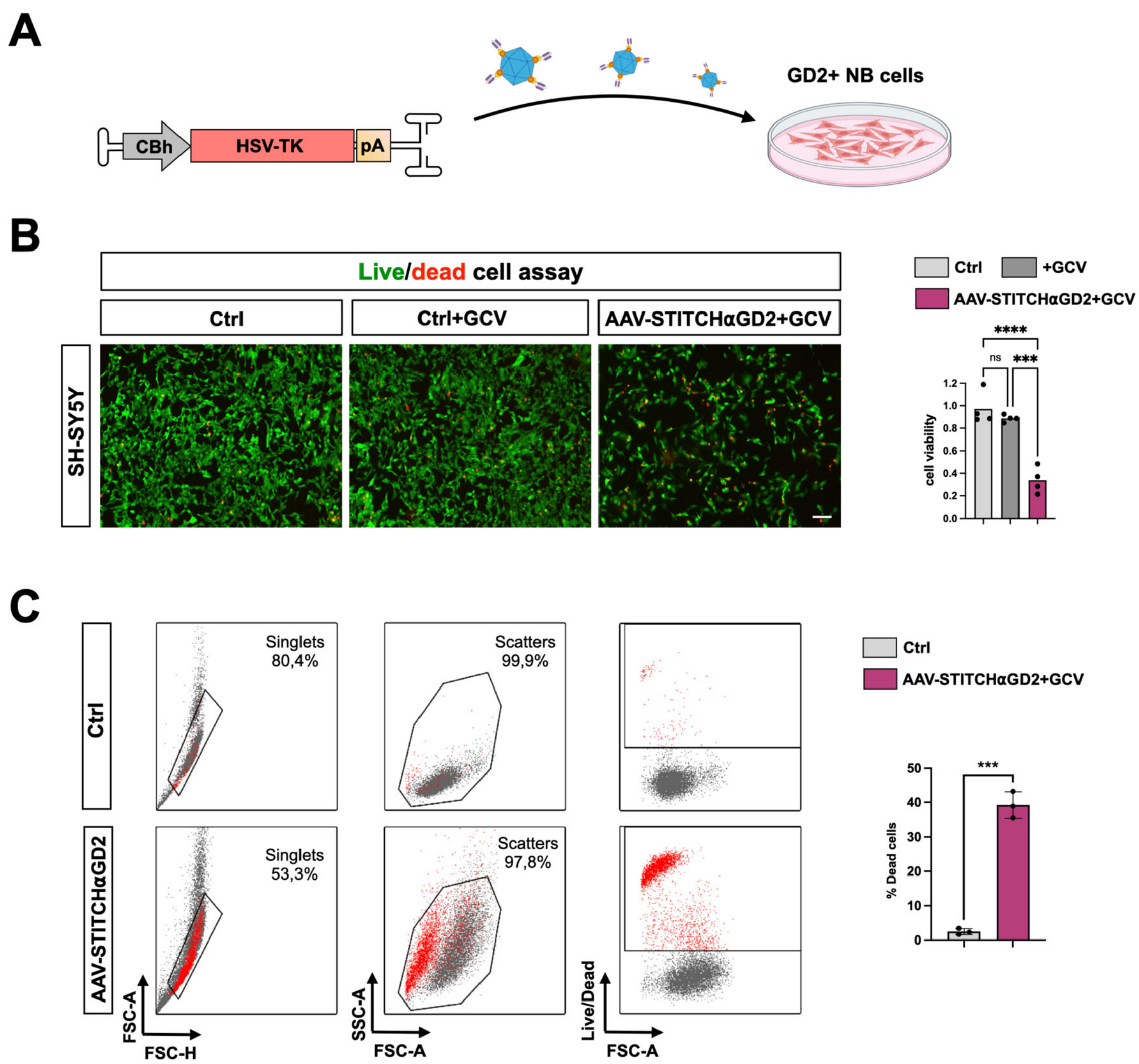

Figure EV7. Evaluation of HSV-TK/GCV-mediated cytotoxicity in GD2+ neuroblastoma cells.

(**A**) Schematic of the experimental protocol used to assess HSV-TK activity in GD2 + SH-SY5Y NB cells. (**B**) Left: Immunofluorescence images using the Live/Dead Assay Kit to evaluate cell death in SH-SY5Y cells transduced with AAV-STITCHαGD2 and treated with Ganciclovir (GCV), compared to non-infected controls with or without GCV treatment. Scale bar: 100 µm. Right: Bar graph showing quantification of cell viability across the three experimental conditions. Data are presented as mean ± SD, with individual data points shown ($n = 4$ independent biological replicates). ***$P = 0.0001$ Ctrl vs AAV-STITCHαGD2 + GCV; ***$P = 0.0008$ + GCV vs AAV-STITCHαGD2 + GCV. One-way ANOVA with Tukey's multiple comparisons correction. (**C**) Left: Flow cytometry analysis of cell populations in control (Ctrl) and AAV-STITCHαGD2-treated samples. Each row represents a different condition: Ctrl (top) and AAV-STITCHαGD2 (bottom). Right: Bar graph showing the percentage of dead cells over total after transduction with AAV-STITCHαGD2 + GCV with respect the untreated control. Data are presented as mean ± SD, with individual data points shown ($n = 3$ independent biological replicates). ***$P = 0.0001$ Ctrl vs AAV-STITCHαGD2 + GCV. One-way ANOVA with Tukey's multiple comparisons correction.

 © The Author(s)

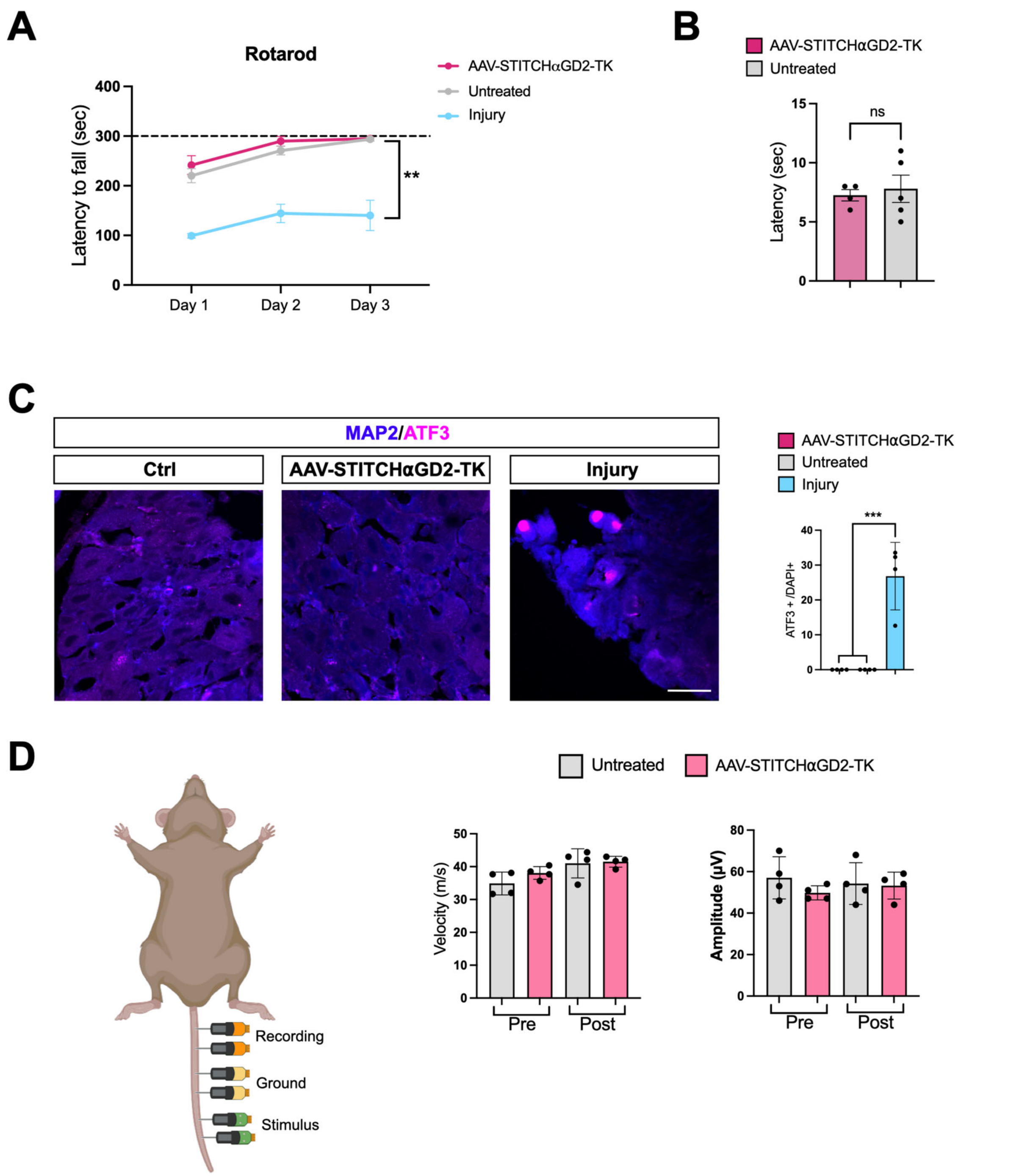

Figure EV8. Toxicity assessment in dorsal root ganglia and peripheral nerves following AAV-STITCHαGD2 injection.

(**A**) Evaluation of the latency to fall from the Rotarod analysis comparing WT untreated mice (gray line), sciatic nerve injured mice (Injury, light blue line) and AAV-STITCHαGD2 carrying HSV-TK (Purple line). $n = 4$ mice per group. Error bars represent SD. **$P = 0.0083$ AAV-STITCHαGD2-TK vs Injury; **$P = 0.0069$ Untreated vs Injury. Two-way ANOVA with Sidak's multiple comparisons correction. (**B**) Evaluation of tactile sensitivity through hot plate test at 52 °C comparing WT untreated mice and treated with AAV-STITCHαGD2 carrying HSV-TK. Data are presented as mean ± SD, with individual data points shown ($n = 4$ mice per group). ns $P > 0.05$. *T* Test. (**C**) Left: immunofluorescence using anti-MAP2 and anti-ATF3 antibodies on DRGs derived from WT untreated mice (ctrl), injury (injured sciatic-nerve) and AAV-STITCHαGD2 carrying HSV-TK. Scale bar: 50 μm. Right: bar graph showing the quantification of ATF3+ cells over total cells (DAPI +) in the three groups of DRGs. Data are presented as mean ± SD, with individual data points shown ($n = 4$ mice per group). ***$P = 0.0001$ AAV-STITCHαGD2-TK vs Injury; ***$P = 0.0001$ Untreated vs Injury. One-way ANOVA with Tukey's multiple comparisons correction. (**D**) Left, schematic representation of tail sensory nerve conduction recordings. Sensory nerve action potentials (SNAPs) were recorded using stimulating and recording electrodes positioned along the tail nerve. Right, quantification of sensory nerve conduction velocity (m/s) and SNAP amplitude (μV) before (Pre) and after (Post) treatment in untreated and AAV-STITCHαGD2-TK + GCV treated mice. Data are presented as mean ± SD, with individual data points shown ($n = 4$ mice per group). Two-way repeated-measures ANOVA with Sidak's multiple comparisons.

© The Author(s)

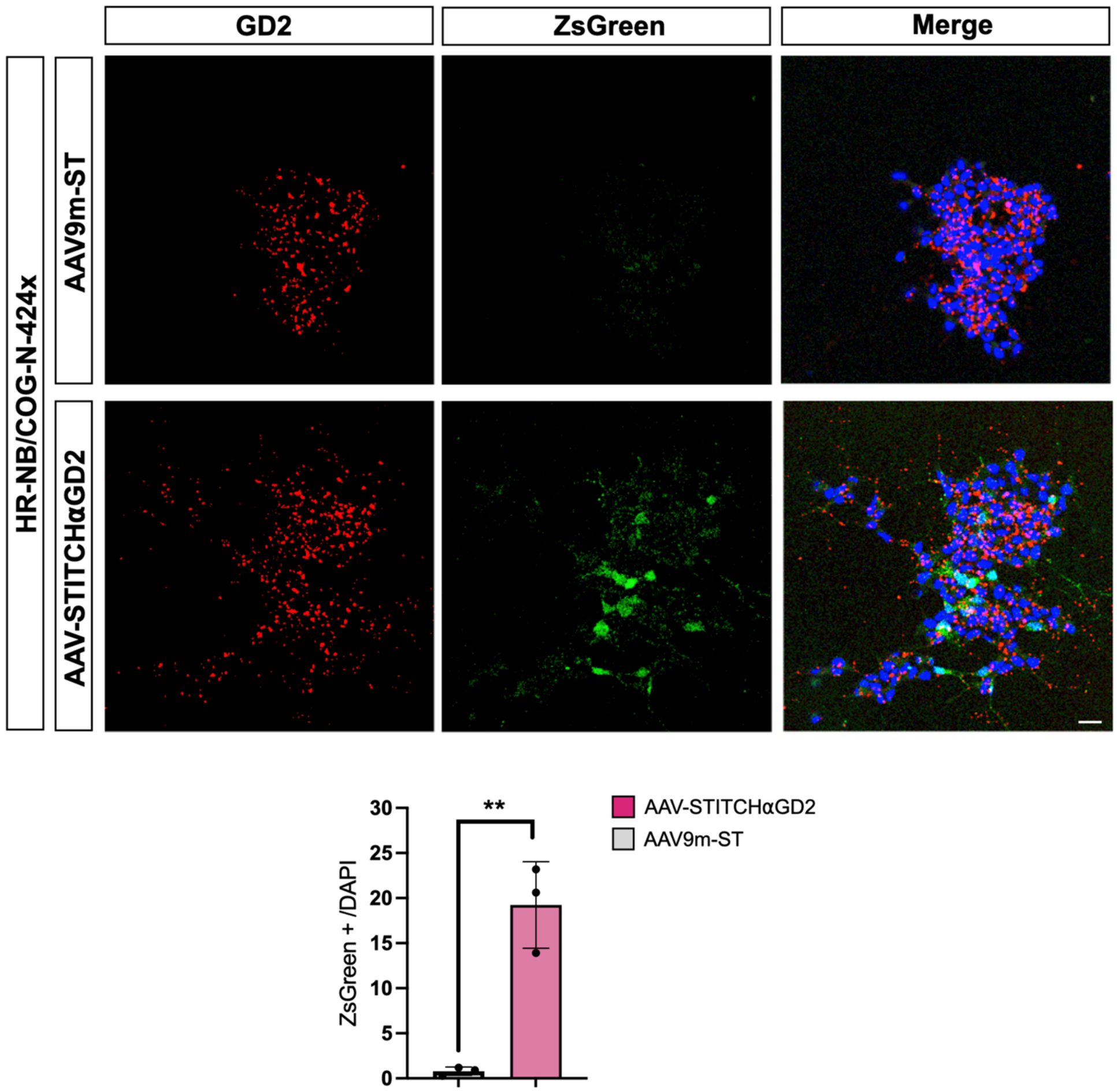

Figure EV9. Evaluation of AAV-STITCHαGD2 transduction efficiency in patient-derived cancer cells.

Representative immunostaining using anti-ZsGreen and anti-GD2 antibodies on patient-derived cancer cells transduced with AAV-STITCHαGD2 or AAV9m-ST as control to vehicle ZsGreen transgene and bar graph showing the percentage of ZsGreen+ cells over total (DAPI+) after transduction with AAV-STITCHαGD2 with respect the control AAV9m-ST. Scale bar: 100 µm. Data are presented as mean ± SD, with individual data points shown ($n = 3$ independent biological replicates). ***$P = 0.0009$, *t* Test.

© The Author(s)
